# A Heterogenized Molecular Catalyst for the Gas-Phase Cyclotrimerization of Acetylene to Benzene

**DOI:** 10.1021/jacs.5c16274

**Published:** 2025-10-30

**Authors:** Jonathan M. Mauß, Sebastian Leiting, Christophe Farès, Anna G. Scott, Sergey Peredkov, Serena DeBeer, Claudia Weidenthaler, Ferdi Schüth

**Affiliations:** † Department of Heterogeneous Catalysis, 28314 Max-Planck-Institut für Kohlenforschung, Kaiser-Wilhelm-Platz 1, 45470 Mülheim an der Ruhr, Germany; ‡ Department of Inorganic Spectroscopy, 28313 Max-Planck-Institut für Chemische Energiekonversion, Stiftstraße 34-36, 45470 Mülheim an der Ruhr, Germany

## Abstract

Facilitating the cyclotrimerization of acetylene to benzene in the gas-phase on the surface of solid catalysts has captivated researchers, both theoretically and experimentally, for several decades. Coupled with acetylene production from renewable feedstocks, this reaction offers a promising pathway for the direct, low-temperature synthesis of renewable benzene. Recognizing the physical limitations of typical solid catalysts, which adsorb benzene too strongly, this study investigates various high-valent early transition metal chlorides—potent molecular cyclotrimerization catalysts—as solid catalysts in this gas-phase conversion. Alongside catalytic assessment under industrially relevant conditions, various analytical techniques such as ^93^Nb *solid-state* NMR spectroscopy, X-ray emission spectroscopy, and quasi in situ X-ray photoelectron spectroscopy were applied to reveal that the catalytic functioning proceeds through the in situ formation of catalytically active reduced species as reported for a homogeneous reaction environment. Investigating deactivation phenomena, various catalyst precursors and reaction conditions led to the design of an immobilized molecular catalyst via chemical grafting, dispersion, and spatial isolation of active catalytic species on a mesoporous silica gel support. The resulting NbCl_*x*_–silica gel catalyst exhibits an average benzene selectivity of up to 70% at an acetylene conversion level between 30% and 70% and a lifetime at ≥90% acetylene conversion of up to 8.5 h under industrially relevant conditions (10 vol % C_2_H_2_, 3 bar, 180 °C), outperforming previously reported solid catalysts by orders of magnitude.

## Graphical abstract

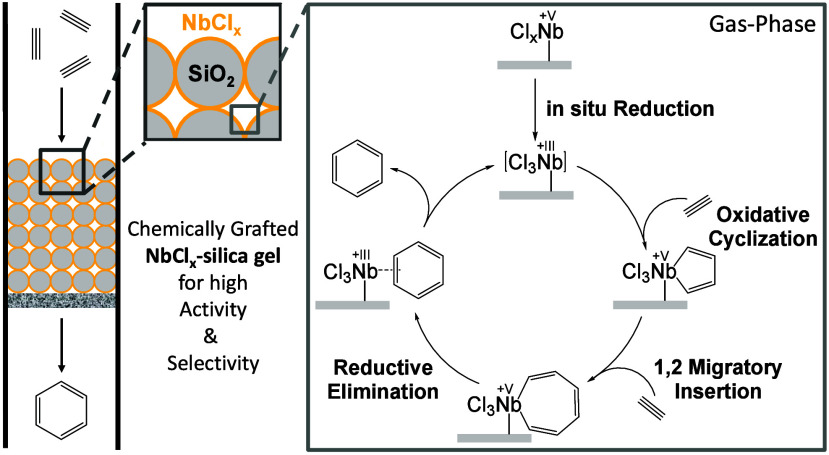

## Introduction

Benzene is an indispensable base chemical in the petrochemical industry, with a predicted global production volume of 64.4 Mt and market value of 66.2 Bn USD in 2024.^1^ It is a key intermediate in the production of various everyday products including plastics (e.g., polystyrene), synthetic fibers (e.g., polyamide) and fine chemicals (e.g., detergents).^2–5^ The increasing usage of polystyrene in the packaging, automotive, and electronics sectors is driving a significant growth in benzene demand, with a predicted CAGR of 6.1% until 2030.^6^ Currently, about 60% of the global benzene production takes place in refineries, primarily as a byproduct of gasoline manufacture via catalytic reforming.^3,7^ However, due to its high carcinogenicity, the benzene content in gasoline is strictly limited by law, leading refineries to reduce benzene levels, which directly affects the product mixtures obtained in steam crackers in the petrochemical industry.^2,3^ This development, combined with concerns about the limited availability of fossil feedstocks and their detrimental environmental impact, has sparked interest in the direct benzene synthesis from renewable resources.^5,8–10^

Multiple pathways are currently being investigated for their economic viability and feasibility in existing infrastructure. These include the pyrolysis or hydrodeoxygenation of vegetable oils, conversion of lignin and polysaccharides to gasoline and aromatics, transesterification or fermentation of vegetable oils and polysaccharides to biodiesel and bioethanol as feedstocks for syngas production via steam reforming, syngas conversion to methanol for methanol-to-aromatics (MTA) or for synthesis and dehydroaromatization of Fischer–Tropsch hydrocarbons (STA), and the dehydroaromatization of methane from biogas or CO_2_ methanation (MDA).^2,5,9^ However, these processes face significant challenges due to high process temperatures, severe deactivation, and overall low yields for benzene, being moreover difficult to extract from the complex product mixtures obtained.^2,5,9,10^

The possibility of converting acetylene into benzene in a single step via [2 + 2+2] cyclotrimerization at low temperatures (<200 °C) represents a promising and unique pathway for direct benzene synthesis. This is particularly interesting because acetylene can be obtained from (bio)­methane produced through biomass fermentation or CO_2_ methanation, using an electric nonthermal plasma-assisted pyrolysis technology.^11–13^ Despite the high thermodynamic driving force for acetylene cyclotrimerization (ΔG_r_^0^ = −503.4 kJ/mol), the reaction is hindered by substantial entropic and kinetic hurdles (250 < *E*_a_ < 335 kJ/mol) due to the energetically intensive breaking of π-bonds, which prevents the reaction from occurring noncatalytically at temperatures below 400 °C.^12,14–16^ In the 1940s, Reppe and Schweckendiek made significant contributions to the development of catalysts in liquid media for this transformation, including nickel(0)–carbonyl-phosphine complexes in aromatic solvents at moderate temperatures (ca. 70 °C) and pressures (ca. 10 bar).^12,14,17^ These complexes, starting from a metallic Ni(0) center, undergo an oxidative cyclization with two coordinated acetylene molecules to form a metallacyclopentadiene intermediate. This intermediate then reacts via a 1,2-migratory insertion or [4 + 2] Diels–Alder type reaction with a third acetylene molecule, followed by a reductive elimination and the liberation of the benzene molecule.^14,18^ The challenge of transferring this trimolecular reactivity to a continuous gas-phase reaction with solid catalysts has captivated researchers for decades, as it offers a more appealing and scalable route for producing a base chemical like benzene.^19^

Theoretical and experimental studies, mostly conducted under ultrahigh vacuum conditions, demonstrate the general feasibility of low-temperature benzene formation from acetylene in the absence of solvents on various metal surfaces (e.g., Pd,^20,21^ Pt,^19,22^ Sn/Pt,^19,23^ Ag,^24^ Cu^25^), transition metal complex fragments or clusters (e.g., Ni,^26,27^ Co,^16,27^ Ru,^28^ Fe,^27^ V^29^), the carbene-like zigzag edges of graphene^30^ and transition metal-ion exchanged zeolites.^26,31^ Benzene formation on (noble) metal surfaces in the absence of solvents has been shown to proceed via a similar C_4_ intermediate, with benzene desorption rate being the limiting factor rather than the rate of surface reaction.^20,32^ Flat-lying, too strongly adsorbed benzene on extended (noble) metal surfaces is a critical precursor for coke (via dehydrogenation), which is detrimental to the selectivity and stability of typical solid catalysts. Lowering surface coverage and weakening benzene adsorption by surface poisoning (e.g., S, Cl, P) is therefore pivotal for substantial benzene desorption and selectivity but not attractive for technical application.^20,32,33^ Investigations into the gas-phase cyclotrimerization of acetylene to benzene in fixed-bed flow reactors under conditions relevant to industry are scarce. Boudjahem and co-workers reported the highest benzene selectivity to date of 52% (without details on the catalytic stability) using hydrazine-reduced nickel nanoparticles supported on silica (C_2_H_2_/H_2_/H_2_O/He 1:4:0.1:15, 40 °C, WHSV 30 000 cm^3^ g_cat_^–1^ h^–1^, 1 atm). However, the acetylene conversion level and productivity reached under these conditions are very low, at 10% and 0.08 g_C6H6_ g_cat_^–1^ h^–1^, respectively.^34^

Given the high activity of molecular catalysts in liquid media and the physical limitations of typical solid catalysts (adsorbing benzene too strongly) in the gas-phase cyclotrimerization of acetylene, the aim of this work is to transfer the redox-based reactivity of molecular catalysts to a heterogeneous gas-phase reaction environment. NbCl_5_ and TaCl_5_, potent molecular alkyne cyclotrimerization catalysts, have been shown to react with acetylene in the absence of solvents.^35,36^ Inspired by this observation, we investigated the potential of various high-valent early transition metal chlorides as solid catalysts for the gas-phase cyclotrimerization of acetylene to benzene in a fixed-bed flow reactor, with the goal of developing a heterogenized molecular catalyst for this chemical transformation.

## Experimental Section

### Materials

ZrCl_4_ (99.5% trace metal basis), HfCl_4_ (98%), NbF_5_ (98%) NbCl_5_ (99%), NbCl_4_(THF)_2_ (≥98.5%), NbCl_3_(DME) (≥95%), MoCl_5_ (99.95% trace metal basis), WCl_6_ (95%) and ReCl_5_ (50.0–53.0 Re metal basis) were purchased from Sigma-Aldrich. NbBr_5_ (≥99.9%) and NbI_5_ (≥99.9%) were acquired from BOC Sciences. Quartz wool (Roth, chemically pure) and silica gel (Supelco, high-purity grade, Davisil grade 646, pore size 150 Å) were used as obtained.

### Catalyst Synthesis

Most catalysts were used as neat compounds as received. For chemically grafting NbCl_*x*_ moieties on a silica surface (see Figure S88), mesoporous silica gel (sieved fraction of 300–400 μm) was first dried in vacuum. After transfer to an Ar filled glovebox, NbCl_5_ was added in the amount corresponding to the targeted loading, light vacuum was applied to the flask and dry toluene was added at room temperature under stirring. The addition of toluene was continued until the complete dissolution of NbCl_5_.^37^ Toluene was removed very slowly by applying lower vacuum. The dark red functionalized silica gel material was subsequently further dried in vacuum until it turned yellow. Commercial and synthesized catalytic materials were stored carefully sealed in an Ar filled glovebox (under cooling if necessary).

### Catalyst Characterization

If not indicated differently all characterization techniques in this study were performed under strict dry and inert conditions. X-ray photoelectron spectroscopy (XPS) was measured on a customized spectrometer from SPECS GmbH with a Phoibos 150 hemispherical energy analyzer, a 1D-DLD detector and a nonmonochromatic Mg radiation source. For the quasi in situ experiment, pristine NbCl_5_ was transferred within the apparatus to a flow-type reaction chamber. The gas chromatography with flame ionization detection or mass spectrometer as detector (GC-FID/-MS) for byproduct identification was performed on a GC 7890B (Agilent) equipped with an FID detector or on a Trace GC Ultra (Thermo Fischer Scientific) directly coupled with a MS detector (Thermo Fischer Scientific). Thermogravimetric analysis coupled with mass spectrometry (TGA-MS) was done on a Netzsch Jupiter STA 449F3 instrument connected to a Netzsch Aëolos QMS 403D mass spectrometer. Nitrogen physisorption (N_2_ physisorption) was conducted at −196 °C on a Micromeritics 3Flex. Inductively coupled plasma optical emission spectroscopy (ICP-OES), ion chromatography (IC) and combustion analysis were performed by Mikroanalytisches Labor Kolbe at Fraunhofer Umsicht in Oberhausen, Germany. ICP-OES measurements were also performed in-house under ambient conditions on a Spectro Green FMX 46 Type 76004566 DSOI equipped with UVplus optics, ORCA, Crossflow and a Scotts chamber. Powder X-ray diffraction (PXRD) measurements for qualitative phase identification were conducted on a Stoe STADI P transmission diffractometer equipped with a Mo radiation source (λ = 0.7093 Å), a primary Ge(111) monochromator (Mo Kα_1_) and a position-sensitive Mythen1K detector. Raman spectroscopy was measured on a Renishaw inVia confocal Raman Microscope using an objective lens with 50× magnification and either an 532 nm (2.5 – 5 mW laser power) or 785 nm laser (0.03–15 mW laser power) at an exposure time of 10 s with 5 accumulations. Attenuated total reflectance Fourier transform infrared spectroscopy (ATR FT-IR) was performed on an Agilent Cary 630 FTIR with diamond crystal. ^1^H nuclear magnetic resonance spectroscopy (1D ^1^H- and ^1^H–^1^H-COSY NMR) was performed on a Bruker AV600neo (600 MHz) instrument with a Cryo-BBO probe head at 25 °C. ^1^H, ^13^C, ^29^Si and ^93^Nb *solid-state* nuclear magnetic resonance spectroscopy (*ss*NMR) was recorded on a Bruker Avance III HD 500WB spectrometer using a double-bearing MAS probe (DVT BL4). To aid with the interpretation of the ^93^Nb *ss*NMR spectra, density functional theory (DFT) calculations were performed using the CASTEP program with the GIPAW method on model structure geometries of different Nb_*x*_Cl_*y*_ complexes. Scanning electron microscopy (SEM) with energy dispersive X-ray spectroscopy (EDX) for bulk analysis and elemental mapping was performed on a Hitachi TM3030 Plus table top scanning electron microscope (SEM) equipped with an Oxford Instruments Xplore Compact 30 detector at an acceleration voltage of 15 kV. High resolution (scanning) transmission electron microscopy (HR-(S)­TEM) with energy dispersive X-ray spectroscopy (EDX) for bulk analysis and elemental mapping was conducted with a Thermo Scientific Talos F200x (scanning) transmission electron microscope equipped with a SuperX EDS system (Velox software) at an acceleration voltage of 200 kV. X-ray emission spectroscopy (Nb Lβ_2_) was measured on an in-house designed energy dispersive vacuum von Hamos spectrometer at the PINK tender X-ray beamline^38^ at BESSY II. More detailed information can be found in the Supporting Information.

### Catalyst Testing

To handle acetylene in the pressurized state safely, several strict safety measures have to be followed. For more detailed information on safety considerations and on the reactor setup see the dissertation of I.-T. Trotus^39^ or previously published studies.^13,40,141^ Catalytic testing was performed using a plug-flow fixed bed reactor (stainless steel 316L, 8 mm i.d.) in a temperature range from 120 to 240 °C at 3 bar pressure. The reactor was heated by means of an external oven equipped with a thermocouple (T_heating_). On stream temperature inside the reactor was measured by a thermocouple immersed into the catalyst bed. Previously calibrated mass flow controllers ensured a controlled flow of gases totaling to a WHSV in the range of 1 700 to 10 300 cm^3^ h^–1^ g_cat_^–1^. The catalytic materials were tested by carefully supporting their powders on a bed of predried quartz wool that was held in place by an underlying metal sieve and a quartz wool plug on top. As an internal standard methane was added to the product stream downstream of the reactor. An online gas chromatograph (Agilent 7890B) was used for qualitative and quantitative analysis of the feed and product gas stream composition. The gas flow of acetylene, ethylene, propene, 1-butene and 1,3-butadiene was determined via the peak area ratio to methane (set to a constant flow) applying response factors from previous calibration. For the determination of the gas flow of benzene (1,3-cyclohexadiene and cyclohexene) via the peak area ratio to methane, the relative sensitivity value for H_2_–FID detectors (extrapolated between cyclohexane and benzene) reported in literature^41^ were applied. The total flow of other volatile minority species was calculated accordingly estimating an average relative sensitivity value of 1. Acetylene (or ethylene) conversion (X_C2H2_), selectivity to benzene, ethylene, propene, 1-butene, 1,3-butadiene, 1,3-cyclohexadiene, cyclohexene and other volatiles (S_C_*x*__) and carbon balance (CB) was calculated via eqs 1–3. For more details see the Supporting Information.

1 $$X_{C_{2}H_{2}}=1-\frac{{ṅ}_{C_{2}H_{2},\mathrm{out}}}{{ṅ}_{C_{2}H_{2},\mathrm{in}}}$$

2 $$S_{C_{x}}=\frac{\left({ṅ}_{C_{x},\mathrm{out}}-{ṅ}_{C_{x},\mathrm{in}}\right)\cdot \left(a_{C_{x}}/2\right)}{{ṅ}_{C_{2}H_{2},\mathrm{in}}-{ṅ}_{C_{2}H_{2},\mathrm{out}}}$$

3 $$\mathrm{CB}=\frac{\overset{6}{\underset{x=1}{\sum }}\left(a_{C_{x}}\cdot {ṅ}_{C_{x},\mathrm{out}}\right)}{\overset{6}{\underset{x=1}{\sum }}\left(a_{C_{x}}\cdot {ṅ}_{C_{x},\mathrm{in}}\right)}$$

## Results and Discussion

### Screening of Early Transition Metal Chlorides

To assess the performance of promising high-valent early transition metal chlorides such as ZrCl_4_, HfCl_4_, NbCl_5_, TaCl_5_, MoCl_5_, WCl_6_ and ReCl_5_ as solid catalysts in the gas-phase cyclotrimerization of acetylene to benzene, the respective materials were carefully supported on quartz wool in a fixed bed reactor and exposed to an acetylene/nitrogen gas flow (1:10, 6 600 cm^3^ h^–1^ g_cat_^–1^) at 150 °C and 3 bar pressure. Figure 1 displays the acetylene conversion and selectivity to benzene and other volatile byproducts after different times on stream (Figures S5–S11). While no benzene formation was observable for ZrCl_4_ and HfCl_4_, low to moderate benzene selectivity was obtained with the other metal chlorides, partially over extended times on stream (up to 19 h). Benzene selectivity and mass activity was highest for NbCl_5_ (48% at X C_2_H_2_ = 88%), followed by WCl_6_ (26% at X C_2_H_2_ = 12%), TaCl_5_ (9% at X C_2_H_2_ = 3%), ReCl_5_ (5% at X C_2_H_2_ = 4%) and MoCl_5_ (4% at X C_2_H_2_ = 4%). Most pronounced in the case of WCl_6_ (Figure S10), the reaction generally starts (in the case of active materials) in a highly exothermic manner, increasing the catalyst bed temperature by up to +16 °C. Benzene formation sets in after this induction period that is most likely linked to the in situ formation of low-valent metal species that are able to perform the catalytic redox cycle, as reported for liquid media.^35,36,42^ Lacking stability for lower oxidation states as well as lower Lewis acidity might explain the absence of measurable catalytic activity for ZrCl_4_ and HfCl_4_ in this regard. The duration of the induction period, based on the buildup of benzene selectivity, varies between the different metal chlorides, ranging from 8 min (NbCl_5_) to almost 2 h (WCl_6_). While the benzene selectivity reaches its maximum after the induction period and then slowly decreases again, acetylene conversion is in all cases significantly dropping after 0.5 to 2 h on stream suggesting severe deactivation phenomena.

**Figure 1:**
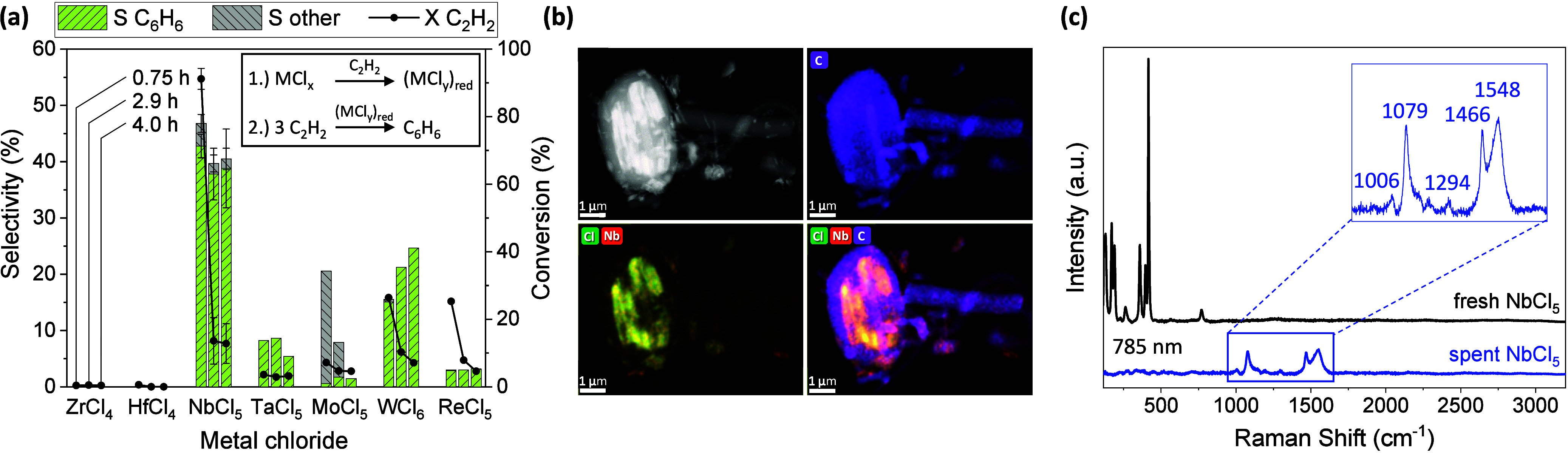
(a) Benzene selectivity, selectivity to other volatiles and acetylene conversion at different times on stream for different metal chloride catalysts (scatter for NbCl_5_ for 4 experiments) in the gas-phase cyclotrimerization of acetylene (C_2_H_2_/N_2_ 1:10, 150 °C, 3–4 bar, WHSV 6 600 cm^3^ h^–1^ g_cat_^–1^) with a reaction scheme at the top right. (b) HR-(S)­TEM image with elemental mapping of spent NbCl_5_ catalyst revealing the encapsulation with carbon deposits. (c) Raman spectra (background corrected) of fresh and spent NbCl_5_ catalyst taken with a 785 nm laser, enlarged signal area for spent catalyst reveals characteristic bands for *trans*-polyacetylene.

Benzene and other volatile byproducts were, besides online GC-FID analysis, also identified via offline GC-MS analysis. Other volatile byproducts could be partially identified as a broad mixture of various (non)­chlorinated unsaturated C_2_ and C_4_ compounds as well as either chlorinated or alkylated (higher) aromatics like toluene, naphthalene and biphenyl (Figure S12). Volatile byproducts are in particular present during the induction period of the catalysts. Especially the chlorinated C_2_ (e.g., cis-1,2-dichloroethylene) and (chlorinated) C–C coupled C_4_ compounds (e.g., diacetylene) might originate from the in situ reduction of the metal chlorides as reported for alkyne cyclotrimerization in liquid media (mechanistic proposal see Figure S13).^43^ Possible reaction pathways to other byproducts under reaction conditions can be seen in Figure S14 in the SI. MoCl_5_, ReCl_5_ and WCl_6_ as the strongest oxidants exhibit the highest selectivity to these species with total selectivity to nonbenzene volatiles, ranging from 8 to 20% during the first hour on stream, further supporting this hypothesis.

### Deactivation Phenomenon

As expected from the overall low carbon balance (<50%) based on volatile (by)­products, the recovered catalyst beds generally increased in weight after reaction, visibly revealing substantial black (sometimes even oily) deposits (Figure S15). Combustion, SEM-EDX and (S)­TEM-EDX analysis suggests a carbon content in the spent NbCl_5_ catalyst between 41 and 70 wt % (Tables S1–S4). TEM elemental mapping of the pristine (Figure S16) and spent NbCl_5_ catalyst (Figure 1b and Figures S17–S19) reveals a thick encapsulation of the catalyst by these carbon deposits being most likely responsible for the fast deactivation with time on stream. Surface morphology investigations with SEM and SEM-EDX elemental mapping, comparing the pristine (Figure S20) and spent NbCl_5_ catalyst (Figures S21–S23), shows that these carbon deposits consist majorly of plate- and fiber-like growths on the catalyst surface. In order to study the type of carbon species in these carbon deposits, XPS of the spent NbCl_5_ catalyst was performed, revealing a complex mixture of different carbon species at the catalyst surface ranging from carbon bound in π-systems (291.0 eV) to carbon in aliphatic (285.0 eV) or graphitic environments (284.5 eV) (Figure S24).^42^ Bulk analysis of the spent NbCl_5_ catalyst via ATR FT-IR spectroscopy reveals the presence of both aromatic and aliphatic species, with bands observed at 3022 cm^–1^ (aromatic/sp^2^ C–H stretch), 2917 cm^–1^ (aliphatic C–H stretch), multiple bands between 1594 and 1439 cm^–1^ (aliphatic C–H bends or aromatic C=C ring modes), bands at 1035 cm^–1^ (aromatic C–H in-plane bend) and 678 cm^–1^ (aromatic C–H out-of-plane bend) (Figure S25). Further analysis of these aromatics was achieved via TGA-MS allowing for the detection of species such as benzene (*m*/*z* 78), toluene (*m*/*z* 91), styrene (*m*/*z* 104), ethylbenzene/xylene (*m*/*z* 106), 4-methylstyrene (*m*/*z* 117) and naphthalene (*m*/*z* 128) (Figures S26–S28). Quenching, dissolving and analyzing surface species of the spent NbCl_5_ catalyst via GC-MS (Figures S29–S30) and ^1^H NMR spectroscopy (Figures S31–S32), further enlarges the scope of aromatic byproducts to a complex mixture of various chlorinated and/or alkylated mono, di- and biphenyls. Raman spectroscopy of the spent NbCl_5_ catalyst (Figure 1c) enabled the analysis of the nondissolvable residue revealing characteristic bands of defect-rich/polydisperse *trans*-polyacetylene at 1006 (out-of-plane C–H deformation), 1079 (overlap C–C/C-H vibration), 1294 (C–H vibration), 1466 (*trans* C=C vibration) and 1548 cm^–1^ (*trans* C=C vibration) (Figures S33–S34).^44,45^ Further corroboration of the presence of polyacetylene, accounting for approximately 80% of the carbonaceous deposit, was obtained through ^13^C CP-MAS *ss*NMR spectroscopy and TGA analysis under air flow (Figures S35–S36).^44,46^ Being the dominant side reaction in liquid-phase cyclotrimerization of acetylene,^35,36,42,43^ this reactivity seems to persist in a gas-phase reaction and is most likely responsible for the characteristic plate- and fiber-like growths^47^ on the catalyst surface in SEM imaging. Under more severe reaction conditions using nondiluted acetylene feeds and higher oven temperature even the formation of graphitic carbon species (D and G band at 1376 and 1603 cm^–1^) can be observed via Raman spectroscopy (Figures S37–S38). An overview over potential pathways to byproducts under reaction conditions is provided in Figure S14.

### In Situ Generation of Reduced Metal Species

Besides the formation of carbonaceous deposits, ICP-OES, SEM-EDX and (S)­TEM-EDX analyses together with IC measurements reveal a significant lowered Nb/Cl ratio in the spent NbCl_5_ catalyst from initially 1: (4.7 ± 0.3) to 1: (3 ± 1) suggesting in situ formation of reduced metal species as observed in liquid media^36,42,43^ (see Table S1). To identify the presence of low-valent niobium species, the pristine and spent NbCl_5_ catalyst were first analyzed via powder X-ray diffraction (PXRD). As depicted in Figures S39–S40, the spent NbCl_5_ exhibits additional reflections attributable to an NbCl_4_ phase after reaction, which were not present in the pristine NbCl_5_. The presence of NbCl_4_ in the spent catalyst was furthermore visible by the formation of characteristic light blue aqua complexes upon contact with humidity or water during the reactor cleaning process (Figure S41).^48^ Since Nb^4+^ itself is unable to perform the catalytic cycle, it is likely that NbCl_4_ formed by the comproportionation of neighboring (catalytically active) Nb^3+^ and (pristine) Nb^5+^ after reaction at the surface-bulk interface.

In order to obtain more detailed information on the oxidation state of niobium, the pristine and spent NbCl_5_ catalyst were subsequently analyzed via ^93^Nb *solid-state* NMR spectroscopy (*ss*NMR). To deal with the substantial quadrupolar interactions of this I = 9/2 nucleus,^49–52^ the WURST-QCPMG pulse program,^53^ consisting of a sequence of broad-band sweeping excitation and inversion pulses, was applied to extract the quadrupole coupling constant *C*_*q*_ and the quadrupole asymmetry parameter η from the full spectrum. Due to the Q-factor limitation of the resonance circuit, the full spectrum was obtained as the coaddition of 14 single spectral fragments with varying carrier frequency (Figure S42). As depicted in Figure 2a, the *ss*NMR signal of the central +1/2 ↔ −1/2 transition (m_*z*_) for pristine NbCl_5_ is strongly broadened, exhibiting an estimated *C*_*q*_ and η value of 80 MHz and 0.25 at 11.7 T, respectively, which is in a similar range as reported for “comparable” niobium (V+) chloride cyclopentadienyl complexes^51^ and other niobium oxide/halogenide systems.^49,50^ DFT-PBE calculations demonstrated good agreement with the experimental measurement in case of the pristine NbCl_5_ (*C*_*q*_ = 71.5 MHz, η = 0.45, Ω = 332 ppm, κ = −0.3, Figure S43). The spent NbCl_5_ reveals a very similar signal shape that exhibits, however, a new peak near 0 ppm but also additional signal density extending toward higher shielding (edge at −3000 ppm). The new Nb species contributing to these new signals cannot be clearly identified, but lower valence species are valid candidates. Direct comparison with the ^93^Nb spectrum of reduced niobium chloride species like NbCl_4_ and Nb_3_Cl_8_ is challenging due to their inherent instability and unavailability in purity form, but also due to their partially strong paramagnetic properties. Their respective NMR signals of the central transition were simulated for comparison via DFT- PBE using crystallographic data (Figure S43). The unusual high chemical shift anisotropy at such strong electric field gradients predicted by the simulations limit however their reliability. Experimental measurements of similar low-valent but more stable ether complexes such as NbCl_4_(THF)_2_ and NbCl_3_(DME) (Figures 2a and S44–S45) provide further hints of potential signal contributions in the region from Nb^3+^ (and Nb^4+^) species. It is worth noting that higher electronic shielding is expected when lowering the oxidation state as reported for other niobium based systems.^50,51^

**Figure 2:**
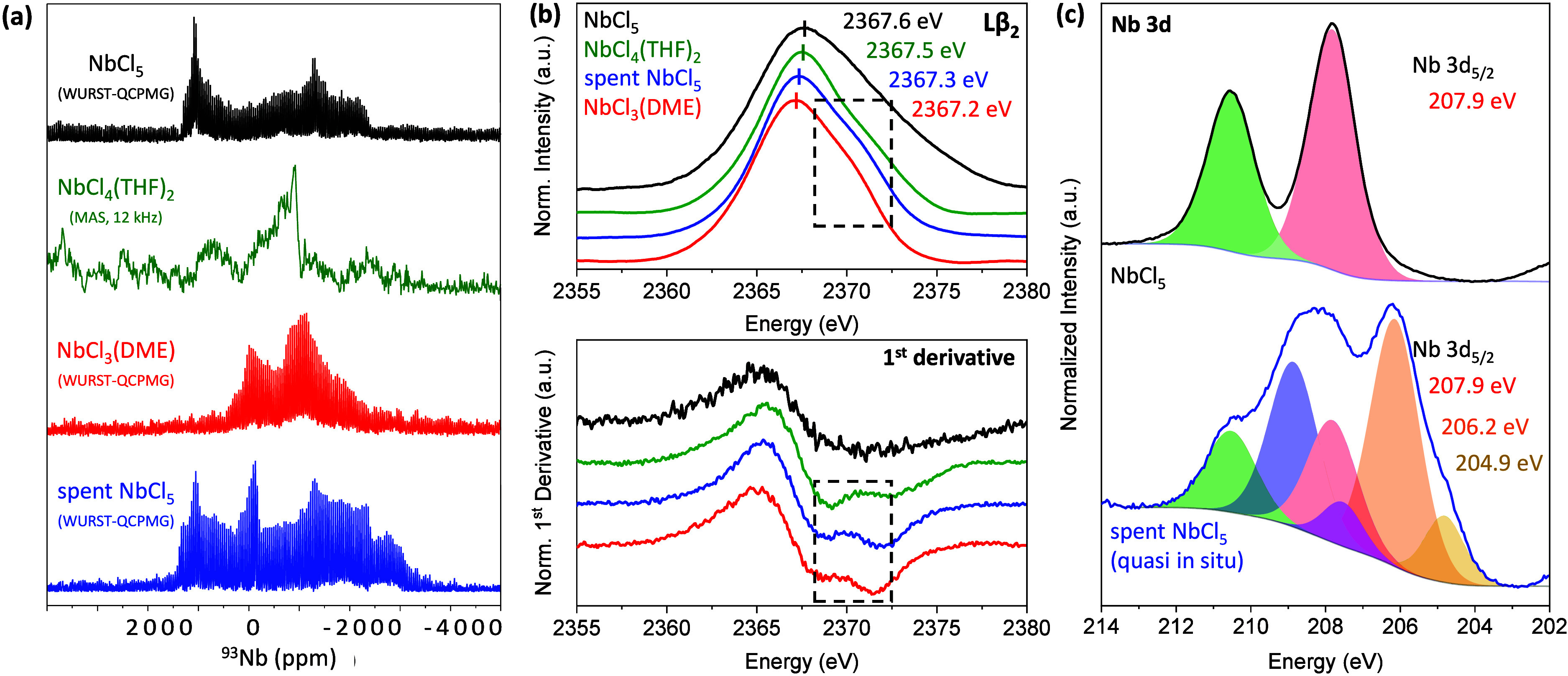
(a) ^93^Nb *solid-state* NMR spectra of the central +1/2 ↔ −1/2 transition (m_*z*_) of fresh NbCl_5_ (black), NbCl_4_(THF)_2_ (green) NbCl_3_(DME) (red) and spent NbCl_5_ (blue) extracted from an overlay of 14 single spectral fragments via a WURST-QCPMG pulse sequence or via MAS with a single excitation pulse. (b) Top: 4d-to-2p X-ray emission spectra for fresh NbCl_5_ (black), spent NbCl_5_ (blue), NbCl_4_(THF)_2_ (green) and NbCl_3_(DME) (red). Spectra were normalized with respect to the maximum intensities. Bottom: first derivatives of the respective X-ray emission spectra revealing the shoulder at higher energies in the case of low-valent niobium species. (c) Quasi in situ XPS measurement of NbCl_5_ before and after exposure to reaction gas (C_2_H_2_/N_2_ 1:49, 120 °C, 1 atm, WHSV ca. 30 000 cm^3^ h^–1^ g_cat_^–1^) with Nb 3d_3/2_/3d_5/2_ in green/red for Nb^5+^, blue/orange for Nb^4+^ and purple/yellow for Nb^3+^, respectively.

Further support for the presence of reduced niobium species in the bulk of the spent NbCl_5_ catalyst was found via nonresonant L-shell X-ray emission spectroscopy (XES). Figure 2b depicts the 4d-to-2p XES spectra obtained for fresh and spent NbCl_5_ as well as for NbCl_4_(THF)_2_ and NbCl_3_(DME). The energy of the maximum of the Lβ_2_ emission line ranges from 2367.6 eV for pristine NbCl_5_ to 2367.2 eV for NbCl_3_(DME), consistent with changes in oxidation states from Nb^5+^ to Nb^3+^. The low-valent niobium chloride species also contain an additional broad shoulder feature at higher energies most likely resulting from transitions from the Nb d-orbitals and/or the π-bonding orbitals localized at the Cl centers (Nb^5+^ - d^0^, Nb^4+^ - d^1^, Nb^3+^ - d^2^).^54^ For NbCl_4_(THF)_2_ and NbCl_3_(DME) this shoulder centers at 2370.5 and 2369.6 eV, respectively. The spent NbCl_5_ exhibits a broad shoulder feature at 2370.0 eV, in between the aforementioned signals, suggesting the presence of both Nb^4+^ and Nb^3+^ species.

To confirm the formation of low-valent niobium species upon contact with acetylene on the surface of the solid NbCl_5_ catalyst, X-ray photoelectron spectroscopy (XPS) was performed for the pristine NbCl_5_ (Figures S46–S47) and spent NbCl_5_ catalyst (Figures S48–S49) and the NbCl_4_(THF)_2_ (Figures S50–S51) and NbCl_3_(DME) references (Figures S52–S53). However, the ex situ results are not conclusive, as the spent NbCl_5_ catalyst and the NbCl_4_(THF)_2_ and NbCl_3_(DME) references all exhibit three different niobium species and potential Nb^x+^-O species from oxygen impurities are also shifted toward lower binding energies compared to their Nb^x+^-Cl counterparts (see XPS of Nb_2_O_5_, Figures S54–S55). To better address the inherent instability of low-valent niobium chloride species, particularly in coordinatively unsaturated surface sites, and rule out potential misinterpretation of Nb^x+^-O species at lower binding energies from oxygen traces during inert sample transfer, a quasi in situ XPS measurement was performed under conditions very close to reaction conditions (Figures S56–S63). Pristine NbCl_5_ was introduced into the apparatus, first measured before reaction (Figures S56–S57), then within the instrument heated to 120 °C under N_2_ flow, subjected to an acetylene/nitrogen gas flow (C_2_H_2_/N_2_ 1:49, 1 atm, WHSV ca. 30 000 cm^3^ h^–1^ g_cat_^–1^) for 1 h, cooled down under nitrogen flow, evacuated and remeasured (Figures S58–S61). Figure 2c depicts the quasi in situ XPS spectra of the Nb 3d region for the pristine and spent NbCl_5_ catalyst. While only one niobium species assignable to Nb^5+^ (3d_5/2_ = 207.9 eV) is observable for the pristine NbCl_5_, three different niobium species (3d_5/2_ = 207.9, 206.2, and 204.9 eV) are present in the spent NbCl_5_. These niobium species are very likely attributable to Nb^5+^, Nb^4+^ and Nb^3+^, respectively, providing evidence for their formation on the surface of NbCl_5_ upon contact with acetylene. After a short contact to air (to reoxidize low-valent species back to Nb^5+^) and remeasurement, these niobium species merge into two (3d_5/2_ = 207.9 and 206.8 eV), belonging presumably to Nb^5+^ bound to chlorine and oxygen, respectively, ruling out an eventual misinterpretation of previous low-valent niobium species in the spent NbCl_5_ catalyst (Figures S62–S63).

### Reaction Conditions and Niobium Catalyst Precursors

To prove the activity of low-valent niobium species in the gas-phase cyclotrimerization reaction of acetylene to benzene, the “stable” ether complexes of Nb^4+^ (NbCl_4_(THF)_2_) and Nb^3+^ (NbCl_3_(DME)) were tested under identical reaction conditions of an acetylene/nitrogen gas flow (1:10, 6 600 cm^3^ h^–1^ g_cat_^–1^) at 150 °C and 3 bar pressure (Figures S7 and S64–S66). No measurable catalytic activity was observed in the case of NbCl_3_(DME), which can be attributed to the lower Lewis acidity of the Nb^3+^ center. This means that it is unable to coordinate gaseous acetylene molecules strongly enough to form organic ligands that are likely necessary for a catalytically active Nb^3+^ species. In case of NbCl_4_(THF)_2_, however, significant catalytic activity with comparable benzene selectivity was observed, albeit lower than for NbCl_5_ and with very rapid deactivation, proving the activity of low-valent niobium species. Furthermore, an increased carbon balance (>70%) via the formation of more volatile byproducts (at least at the beginning of the reaction) indicates that the formation of the nonvolatile carbonaceous deposit (particularly polyacetylene) seems to be closely related to the Lewis acidity.

In order to further improve catalytic performance by enhancing the in situ formation of low-valent niobium species from NbCl_5_ and reducing the formation of carbonaceous deposits (or potentially inactivating organyl ligands), an attempt was made to replace nitrogen by hydrogen in the feed (Figures S7 and S67–S70). While no effect on initial activity or deactivation was observed at 150 °C, only a slight increase in activity with, however, a similar deactivation rate was measured at lower temperature (135 °C). Additionally, no effect on the formation of polyacetylene was observable via Raman spectroscopy of the spent catalyst (Figure S71). Substantial reduction of NbCl_5_ by hydrogen gas does not proceed at temperatures below 240 °C, as reported by Harjanto and co-workers.^55^ Co-feeding hydrogen, however, decreased benzene selectivity, forming other byproducts such as 1,3-butadiene, 1-butene, propene and ethylene (and in small traces even 1,3-cyclohexadiene and cyclohexene at 150 °C) identified by GC-FID and partially GC-MS (Figure S72). These byproducts are likely to result from the hydrogenation of reaction intermediates.

In an attempt to activate the catalyst in an alternative manner, NbCl_5_ was pretreated at 150 °C with ethylene before reaction start (Figures S7 and S73–S75). Ethylene alone is not cyclotrimerized by NbCl_5_ to cyclohexane (as also reported in liquid media^56^) but seemingly coordinates to the Lewis acid during pretreatment, as it is desorbed stepwise again during subsequent reaction with acetylene (Figure S74). Even though no significant increase in activity and only a small increase in benzene selectivity is observed after pretreatment, a significantly more exothermic first contact reaction of the pretreated NbCl_5_ with the acetylene feed (catalyst bed temperature 208 °C) is measurable compared to the untreated NbCl_5_ (catalyst bed temperature 167 °C) (Figures S7 and S74). Intrigued by these results, nitrogen was replaced stepwise with ethylene as cofeed to acetylene during reaction from a C_2_H_2_/C_2_H_4_/N_2_ ratio of 1:0:10 to 1:10:0 at 135 °C (Figures 3a, S68 and S76–S79). While the initial conversion at 0.75 h on stream significantly increases stepwise from 49% (C_2_H_2_/C_2_H_4_/N_2_ 1:0:10) to 89% (C_2_H_2_/C_2_H_4_/N_2_ 1:10:0), benzene selectivity gradually decreases from 41% to 23%, respectively, as depicted in Figure 3a. Despite the decrease in benzene selectivity, the carbon balance generally increases with more ethylene cofed due to a higher selectivity to other volatiles (from 2% up to 11%) and the formation of cocyclized products of acetylene and ethylene such as 1,3-cyclohexadiene (up to 17%) and cyclohexene (up to 1%), both identified via GC-FID and GC-MS (Figure S80). Alongside the carbon balance, the deactivation rate also significantly decreases with increasing ethylene cofeed. As indicated by the cocyclization products, it is highly likely that cofeeding ethylene leads to the formation of more saturated and less reactive (by)­products that undergo fewer follow-up reactions to heavier, nonvolatile species. Deactivation is thus to some extent suppressed. Raman spectroscopy of the spent NbCl_5_ catalyst, however, demonstrates that polyacetylene formation is not (completely) suppressed by ethylene cofeeding, as sharper Raman bands and even visible overtones still indicate polyacetylene, but of a more defect-free/(shorter) monodisperse character (Figure S81).^44,45^ Even though ethylene cofeeding thus does not seem to be of practical utility for benzene formation, these results are interesting at a fundamental level, as cocyclization reactions of even triple and double bonds, driven by the respective partial pressure, are seemingly possible in a gas-phase reaction with this catalyst.

**Figure 3:**
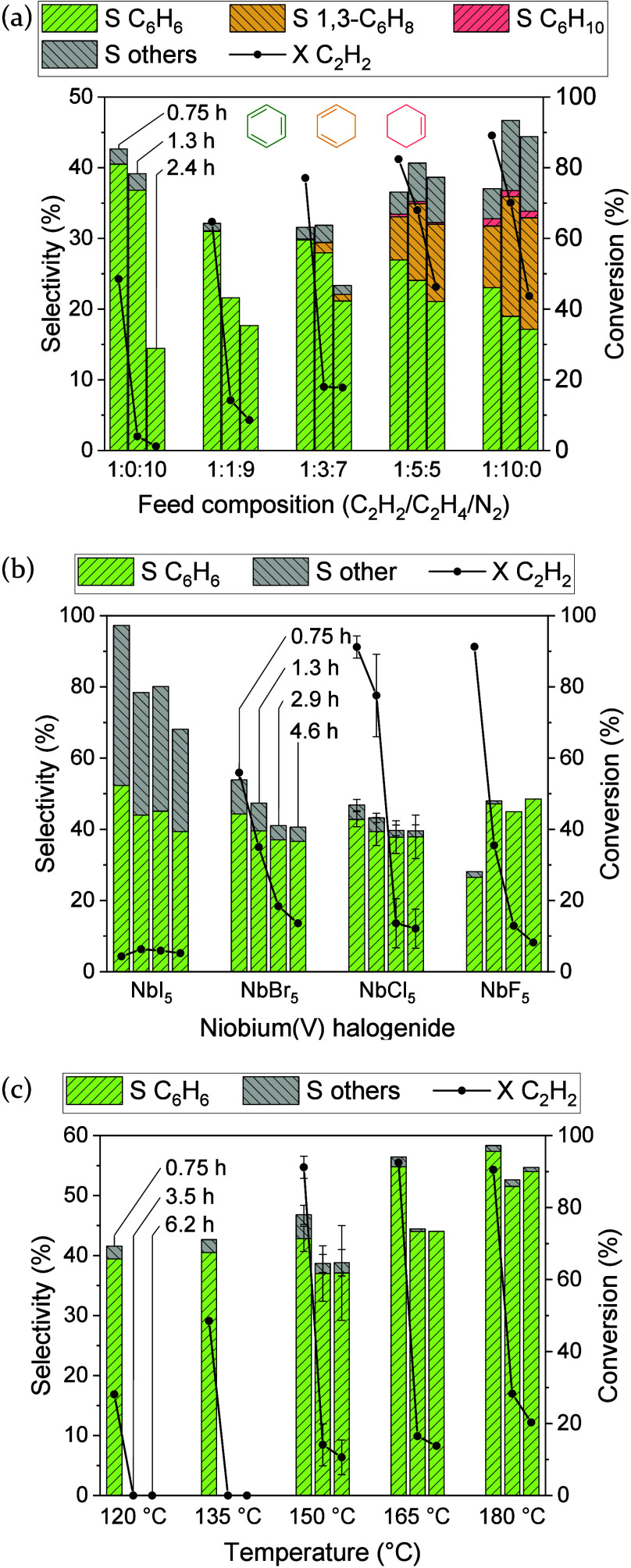
Benzene selectivity, selectivity to other volatiles and acetylene conversion (scatter for 4 experiments) at different times on stream in the gas-phase cyclotrimerization of acetylene (C_2_H_2_/N_2_ 1:10, 3 bar, WHSV 6 600 cm^3^ h^–1^ g_cat_^–1^) for (a) NbCl_5_ with stepwise replacement of nitrogen by ethylene in the feed at 135 °C, (b) different niobium pentahalogenides at 150 °C and (c) NbCl_5_ at different temperatures.

To investigate the effect of Lewis acidity on the catalytic performance the different pentahalogenides of niobium, NbF_5_, NbCl_5_, NbBr_6_ and NbI_5_ were tested (Figures S7, S82–S84). Figure 3b compares their catalytic performance at different times on stream under an acetylene/nitrogen gas flow (1:10, 6 600 cm^3^ h^–1^ g_cat_^–1^) at 150 °C and 3 bar pressure. As expected, the mass activity decreases significantly with the increasing molecular weight of the penthalogenides from 91% acetylene conversion at 0.75 h on stream for NbF_5_ to only 4% for NbI_5_. While no significant improvement in benzene selectivity can be observed by lowering the Lewis acidity from NbF_5_ to NbI_5_, the selectivity to other volatiles increases significantly in this direction. It is likely that a reduced Lewis acidity leads to lower polyacetylene formation, favoring other side-reactions to volatiles. In addition, slightly lowered deactivation rate is observed. The constant benzene selectivity independent of the halogenide, however, further strengthens the hypothesis that the catalytically active species is formed in situ, largely independently of the catalyst precursor, and does not necessarily contain halogenides but rather other organic ligands.

To see if sluggish benzene desorption kinetics limits activity, benzene selectivity and lifetime, the reaction temperature for the NbCl_5_ catalyst was varied from 120 to 180 °C under otherwise identical reaction conditions (C_2_H_2_/N_2_ 1:10, 6 600 cm^3^ h^–1^ g_cat_^–1^, 3 bar, Figures S7, S68, S85–S87). Figure 3c summarizes the catalytic performance at different times on stream for the different temperatures. Indeed both benzene selectivity and acetylene conversion significantly increase with temperature. At 0.75 h on stream, they range from 40% benzene selectivity at 28% acetylene conversion at 120 °C to 57% benzene selectivity at 91% acetylene conversion at 180 °C. Slightly decreasing selectivity to other volatiles and decreasing deactivation rate with temperature suggest that strongly adsorbed benzene is prone to undergo side-reactions to nonvolatile substituted or polyaromatic compounds which can be partially avoided at higher temperatures. Further increased reaction temperatures were not possible due to the low melting temperature of NbCl_5_ (*T*_m_ ∼ 203 °C^57^) and resulting reactor blockage.

### Chemically Grafting NbCl_5_ to Mesoporous Silica Gel

To increase the dispersion of the active species and facilitate handling of the catalyst, NbCl_5_ was chemically grafted to a mesoporous silica gel (300–400 μm, 302 m^2^/g, 15 nm pores) by a surface reaction with the hydroxyl groups of the support in a toluene solution of NbCl_5_ at room temperature (see the Experimental Section, Figures 4a and S88). The molar ratio of niobium to surface hydroxyl group (4.9 OH/nm^2^ ^58^) was varied in a range from 0.1 to 1.1 in order to achieve different loading and dispersion levels of NbCl_*x*_ species. In case of the lowest loading the initially characteristic dark red-colored reaction solution was completely colorless after grafting, demonstrating a quantitative conversion to mono- and bipodal Nb­(-O−)_1–2_Si species (Figures 4a and S89), as also reported elsewhere.^59^ Nonreacted solubilized NbCl_5_ at higher loadings most likely coordinates to these monomeric species at the silica gel surface during slow solvent evaporation. This would result in the formation of increasingly less spatially separated dimeric species that upon in situ reduction during catalysis should form covalent Nb–Nb bonds to the initially grafted species, as typical for low-valent niobium chlorides^57^ (Figures 4a and S89). ATR FT-IR spectroscopy demonstrates the successful deposition of NbCl_*x*_ moieties on the silica gel support (Figure S90). This finding is further corroborated by a decreasing ratio of Q^3^/Q^4^ species (surface single silanol/bulk silicon) in ^29^Si (CP-)­MAS *ss*NMR spectroscopy when transitioning from pristine silica gel to the NbCl_*x*_-functionalized material (Figure S91). A more precise analysis of the formed NbCl_*x*_ species via Raman spectroscopy was not possible due to low signal to fluorescence ratio (Figure S92). The achieved loading of NbCl_*x*_ species (5 to 33 wt %) and the increasing Cl to Nb ratio (3.0 to 3.6) of the functionalized silica gel materials was verified via ICP-OES and IC measurements (Table S5). N_2_ physisorption measurements, depicted in Figure 4b, demonstrate the full accessibility of the porous network after surface functionalization with a slight decrease of the average pore size (BJH-adsorption) from 14.4 to 12.2 nm with increasing NbCl_*x*_ loading (Table S6, Figures S93–S94). HR-(S)­TEM imaging with elemental mapping (Figures 4c and S95–S103) demonstrates the very high dispersion of the NbCl_*x*_ functionalities on the silica gel support, independent of the loading. Solely in the case of the highest NbCl_*x*_ loading is a ca. 1 nm thick surface coating of the silica particles visible (Figure S95). Upon longer irradiation with the electron beam in the microscope small nanoagglomerates also become visible in the case of lower NbCl_*x*_ loadings (Figures S96–S103).

**Figure 4:**
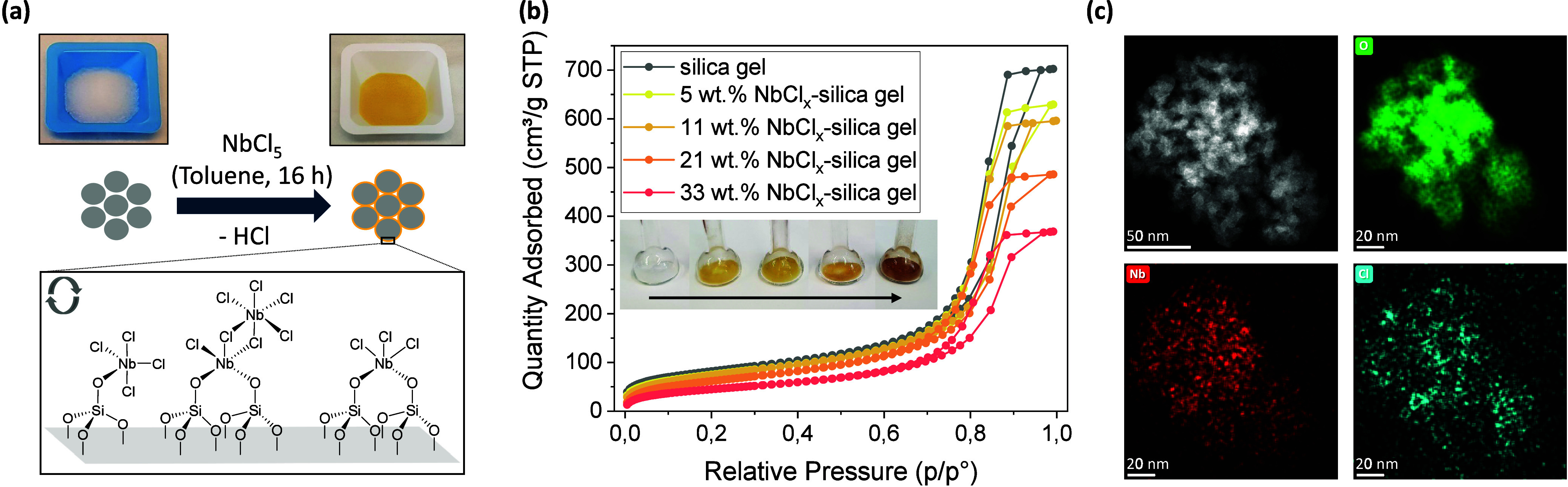
(a) Schematic surface functionalization via the reaction of NbCl_5_ dissolved in toluene with surface hydroxyl groups of dispersed mesoporous silica gel particles at room temperature to monomeric and dimeric mono- or bipodal Nb­(−O−)_1–2_Si species. Surface species adapted from ref 59. Copyright 2015 American Chemical Society. (b) N_2_ physisorption data of silica gel materials functionalized with different amounts of NbCl_5_. Inlet image shows the respective materials with increasing NbCl_*x*_ loading. (c) HR-(S)­TEM image with respective elemental mappings for O, Nb and Cl for the 21 wt % NbCl_*x*_-silica gel material.

The NbCl_*x*_ functionalized materials were used under similar but adapted reaction conditions as the bulk NbCl_5_ catalyst (C_2_H_2_/N_2_ 1:10, 180 °C, 3 bar), to take the up to four times higher catalytic activity per Nb (WHSV 66 000 to 80 000 cm^3^ h^–1^ g_Nb_^–1^ (supported) and 19 000 cm^3^ h^–1^ g_Nb_^–1^ (bulk)) and lower deactivation rate into account. Figure 5a compares the selectivity to benzene and other volatiles for the NbCl_*x*_ functionalized silica gels and the bulk NbCl_5_ catalyst averaged over an acetylene conversion range between 70% and 30%. The catalytic performance remains quite constant for each material over this broad conversion range, offering ideal conditions for a comparison (Figures S87, S104–107). However, the activity of the 5 wt % NbCl_*x*_-silica gel material was too low to be measurable with the given reactor dimensions in this conversion range. Given the discolored reaction solution, it is very likely that this material contains mostly monomeric NbCl_*x*_ species that might not be active in the reaction due to the low Lewis acidity while having oxygen atoms as strong π-donors bound next to the Nb center. For the other materials, the average benzene selectivity significantly increases, from 50% for the bulk NbCl_5_ to a maximum of 70% for decreasing loadings of NbCl_*x*_ species (21 and 11 wt %). The selectivity to other volatiles decreases from 1 to 0%. We hypothesize that this selectivity improvement with increasing dispersion and increasing relative ratio of inactive monomeric to active dimeric NbCl_*x*_ species on the silica gel support might be linked to a spatial site isolation effect. Being surrounded by monomeric NbCl_*x*_ species with lower Lewis acidity, reaction intermediates (aromatic or polymeric) can presumably desorb quicker from active centers, avoiding sequential reactions (e.g., Friedel–Crafts-Alkylation, chlorination, etc.) to potentially nonvolatile compounds (Figure S108), which increases benzene selectivity.

**Figure 5:**
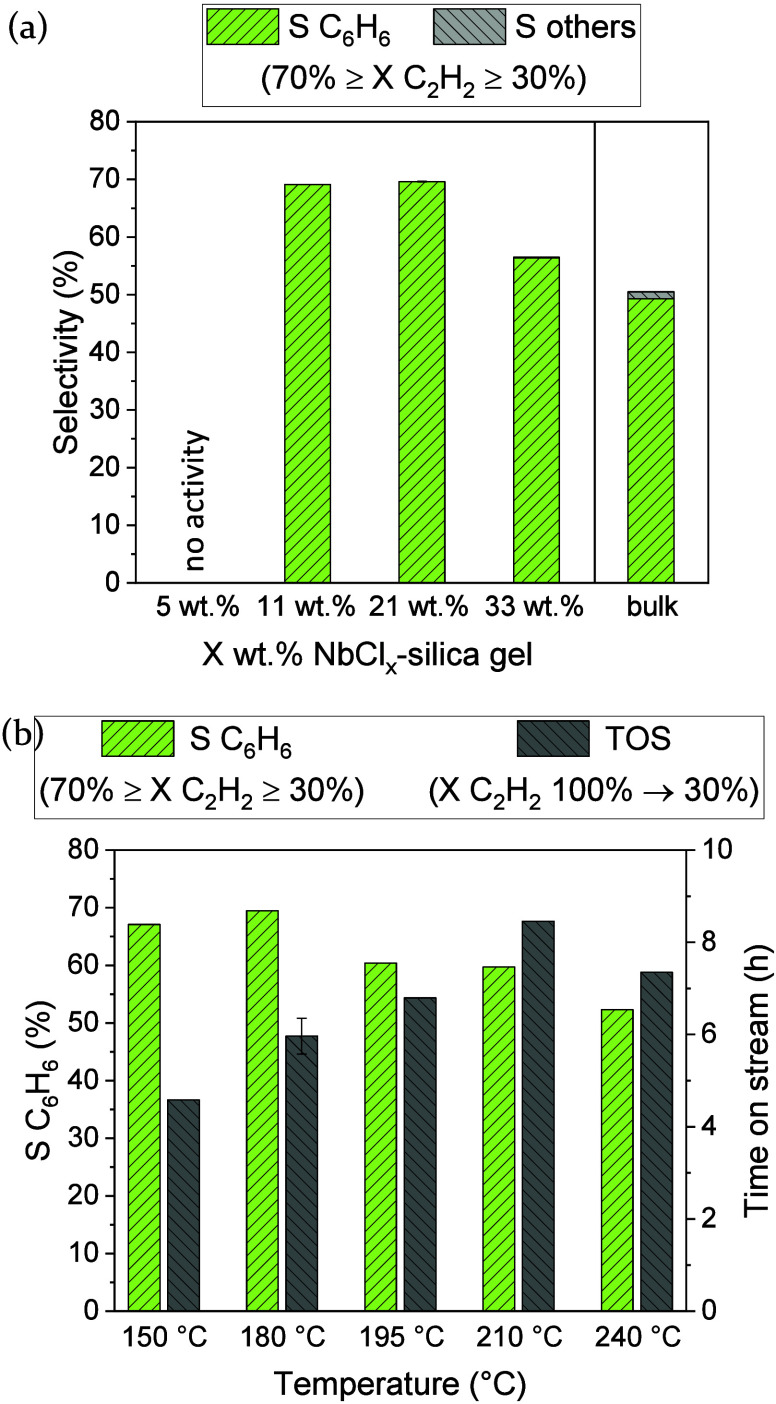
Average benzene selectivity and selectivity to other volatiles (scatter for 2 experiments) in the acetylene conversion range between 70% and 30% in the gas-phase cyclotrimerization of acetylene (C_2_H_2_/N_2_ 1:10, 3 bar, WHSV 66 000 – 80 000 cm^3^ h^–1^ g_Nb_^–1^ (supported) and 19 000 cm^3^ h^–1^ g_Nb_^–1^ (bulk)) for (a) different loadings of NbCl_*x*_ species on mesoporous silica gel in comparison to the bulk NbCl_5_ catalyst at 180 °C and (b) 21 wt % NbCl_*x*_-silica gel at different temperatures with the time on stream until the initial acetylene conversion (ca. 100%) drops to 30%.

As a result of the increased dispersion and lower selectivity to nonvolatile compounds, the deactivation rate of the composites is also decreasing with the loading, being approximately up to three times lower for 21 wt % NbCl_*x*_-silica gel material than for the bulk NbCl_5_. Normalized to a comparable weight hourly space velocity per Nb, the time on stream with an acetylene conversion above 90% can be increased going from the bulk NbCl_5_ from <1 to 8.5 h for the 21 wt % NbCl_*x*_-silica gel material (Figures S87 and S106, S109–S111). Increased weight after catalysis (+(51 ± 5) wt % for 21 wt % NbCl_*x*_-silica gel), N_2_ physisorption (Table S7, Figures S112–S114) and combustion analysis (36.0 wt % C for 21 wt % NbCl_*x*_-silica gel, Table S8) of the spent NbCl_*x*_-silica gel materials indicate a deactivation via the formation of carbon deposits. This causes a loss in surface area, pore volume and pore size, which increases with lower dispersion of NbCl_*x*_ species and coverable support surface area (Tables S6–S7). HR-(S)­TEM images with elemental mapping further evidence the formation of a carbon overlayer, likely encapsulating support and active sites alongside a partial agglomeration of NbCl_*x*_ species during reaction (Figure S115). Increasing the reaction temperature from 180 to 240 °C for the 21 wt % NbCl_*x*_-silica gel catalyst further slows down deactivation rate as indicated by an increasing time on stream until acetylene conversion drops from initially close to 100% to less than 30%, as depicted in Figure 5b. A temperature beyond 180 °C is, however, detrimental to benzene selectivity decreasing from an average value of 70% at 180 °C to 52% at 240 °C (Figures S116–S119). Higher temperatures most likely facilitate the desorption of reaction intermediates, slowing down the formation of carbon deposits. On the other hand, higher temperatures might be harmful for the catalytic active species (e.g., comproportionation with neighboring Nb^5+^ sites or removal of decisive organic ligands). Reforming of the catalytically active species on-stream would consume additional acetylene, lowering the overall benzene selectivity. Although postcatalysis characterization of the spent catalyst did not suggest significant niobium loss (Table S8), further research is needed to investigate the possibility of volatile niobium minority species, particularly with respect to the requirements for polymer-grade benzene. At optimal reaction conditions of 180 °C the 21 wt % NbCl_*x*_-silica gel catalyst achieves a productivity of 1.60 g_C6H6_ g_cat_^–1^ h^–1^, significantly outperforming by a factor of 20 the best Ni/silica catalyst reported so far by Boudjahem and co-workers (0.08 g_C6H6_ g_cat_^–1^ h^–1^, C_2_H_2_/H_2_/H_2_O/He 1:4:0.1:15, 40 °C, 1 atm, WHSV 30 000 cm^3^ g_cat_^–1^ h^–1^, Table S9) underlining the benefits of a heterogenized molecular catalyst for this reaction.^34^ Further optimization of the molecular moiety, particularly with regard to reducing the formation of nonvolatile species, could render this catalyst design promising for future industrial applications. Both niobium pentachloride and silica gel are inexpensive, nontoxic and abundant. Functionalizing the final pellets allows addressing the generally low attrition resistance of silica gels with suitable binders, while efficiently exploiting the high reactivity of the grafted molecular catalyst.

## Conclusion

Several high-valent early transition metal chlorides were investigated as solid catalysts in the gas-phase cyclotrimerization of acetylene to benzene. With a benzene selectivity of 48% at 88% acetylene conversion, NbCl_5_ provides both the highest mass activity and selectivity in this reaction, followed by WCl_6_, TaCl_5_, ReCl_5_ and MoCl_5_. Other, mostly nonvolatile, byproducts were identified as various chlorinated and alkylated acetylene oligomers with aliphatic and aromatic character. Lighter, volatile byproducts could partially be correlated with the in situ formation of the catalytically active reduced metal species. In the case of the spent NbCl_5_ catalyst, the presence of low-valent species (Nb^4+^ and Nb^3+^) could be evidenced via PXRD, ^93^Nb *ss*NMR spectroscopy, XES and quasi in situ XPS, suggesting a similar catalytic functioning as in a homogeneous reaction environment. Nonvolatile byproducts, such as polyacetylene, lead to the formation of carbon deposits that increasingly encapsulate the catalyst over time, causing its rapid deactivation. Co-feeding hydrogen or ethylene can, in the case of the latter, increase activity and slow down deactivation, but both are detrimental to benzene selectivity, forming hydrogenated intermediates or cocyclized products. Other catalyst precursors with lower oxidation states or other pentahalogenides result in similar benzene selectivity, suggesting the formation of similar catalytically active species. Lower Lewis acidity of the niobium center appears to reduce the formation of nonvolatile byproducts, but can be detrimental to catalytic activity. Higher temperatures increase both activity and benzene selectivity, while slowing down deactivation, presumably via an accelerated desorption of reaction intermediates. Chemical grafting of NbCl_*x*_ moieties, as spatially isolated dimeric species, on a mesoporous silica gel support significantly increases benzene selectivity (to 70%), catalyst activity per Nb (by around three times) and reduces the deactivation rate (by around three times). With a productivity of up to 1.60 g_C6H6_ g_cat_^–1^ h^–1^, the composite catalysts significantly outperform previously reported solid catalysts for this gas-phase reaction, demonstrating the strengths of a heterogenized molecular catalyst design for this challenging gas-phase conversion.

## Supplementary Material



## Data Availability

All XES data generated and analyzed during this study are available in the Edmond Open Research Data Repository (Edmond Open Research Data Repository, 10.17617/3.HO6LII).

## Notes

Additional experimental details, photographs of reactor setup, analytical and catalytic raw data (PDF)

## Abbreviations

HR-(S)­TEM: high resolution (scanning) transmission electron microscopy
SEM: scanning electron microscopy
EDX: energy dispersive X-ray spectroscopy
ICP-OES: inductively coupled plasma optical emission spectrometry
IC: ion chromatography
GC-FID: gas chromatography with flame ionization detection
GC-MS: gas chromatography with mass spectrometry detection
PXRD: powder X-ray diffractometry
*ss*NMR: *solid-state* nuclear magnetic resonance spectroscopy
XES: X-ray emission spectroscopy
BET: Brunauer–Emmett–Teller
XPS: X-ray photoelectron spectroscopy
ATR FT-IR: attenuated total reflectance Fourier transform infrared spectroscopy
TGA-MS: thermogravimetric analysis coupled with mass spectrometry detection
COSY: correlation spectroscopy
DFT-PBE: density functional theory applying Perdew–Burke–Ernzerhof method
WURST-QCPMG: wideband, uniform rate, smooth truncation-quadrupole Carr–Purcell–Meiboom–Gill
MAS: magic angle spinning
CP: cross-polarization
X: conversion
S: selectivity
TOS: time on stream
WHSV: weight hourly space velocity
