# Supplementary material for: A Heterogenized Molecular Catalyst for the Gas-Phase Cyclotrimerization of Acetylene to Benzene
Source: J Am Chem Soc. 2025 Oct 30;147(45):42088–99. doi: 10.1021/jacs.5c16274 (PMC12616696; doi:10.1021/jacs.5c16274)
Supplement: Supplementary file 1 [file ja5c16274_si_001.pdf]

Supporting Information

A Heterogenized Molecular Catalyst for the Gas-Phase Cyclotrimerization of Acetylene to Benzene

Jonathan M. Mauß [1], Sebastian Leiting [1], Christophe Farès [1], Anna G. Scott [2], Sergey Peredkov [2], Serena DeBeer [2], Claudia Weidenthaler [1], Ferdi Schüth [1*]

[1] Department of Heterogeneous Catalysis, Max-Planck-Institut für Kohlenforschung, Kaiser-Wilhelm-Platz 1, 45470 Mülheim an der Ruhr, Germany

[2] Department of Inorganic Spectroscopy, Max-Planck-Institut für Chemische Energiekonversion, Stiftstraße 34-36, 45480 Mülheim an der Ruhr, Germany

* Email: schueth@kofo.mpg.de

EXPERIMENTAL

Materials

$ZrCl_4$ (99.5% trace metal basis), $HfCl_4$ (98%), NbF_5 (98%) $NbCl_5$ (99%), $NbCl_4(THF)_2$ (≥98.5%), $NbCl_3(DME)$ (≥95%), $MoCl_5$ (99.95% trace metal basis), WCl_6 (95%) and $ReCl_5$ (50.0-53.0 Re metal basis) were purchased from Sigma Aldrich and used as received. $NbBr_5$ (≥99.9%) and NbI_5 (≥99.9%) were acquired from BOC Sciences and used as received. Quartz wool (Roth, chemically pure) and silica gel (Supelco, high-purity grade, Davisil Grade 646, pore size 150 Å) were used as obtained.

Catalyst synthesis

Most catalysts were used as neat compounds as received (for reactor filling, see below). For chemically grafting $NbCl_x$ moieties on a silica surface (see Figure S88), mesoporous silica gel (sieved fraction of 300-400 µm) was first pre-dried at 150 °C in a drying oven for 3 h, then 5.2 g of the dried silica gel was transferred to a dried Schlenk flask and further dried in vacuum (ca. 10^{-2} mbar) at 80 °C under constant slow stirring for at least 16 h. After transfer to an Ar filled glovebox, $NbCl_5$ was added in the amount corresponding to the targeted loading, light vacuum (ca. 100 mbar) was applied to the flask and dry toluene was added at room temperature under stirring and active vacuum sucking (ca. 100 mbar) via cannula transfer accompanied by an exothermic heat up of the reaction solution and the formation of HCl gas bubbles. The addition of toluene was continued until a $NbCl_5$ concentration of 7.0 g/L (0.8 g_{NbCl5} / 100 $g_{toluene}$) was achieved, ensuring a complete dissolution of $NbCl_5$ in a characteristic dark-red color.[1] The solution with the silica gel was stirred for at least 16 h, before toluene was removed very slowly over the course of 3-7 h by applying lower vacuum. The dark red functionalized silica gel material was subsequently further dried in vacuum (ca. 10^{-3} mbar) until it turned yellow indicating the complete removal of toluene. Commercial and synthesized catalytic materials were stored carefully sealed in an Ar filled glovebox (under cooling if necessary).

Catalyst characterization

If not indicated differently all characterization techniques, sample handling and storage in this study were performed under strict dry and inert conditions using nitrogen or argon filled gloveboxes and special inert gas sample holders. High-valent early transition metal chlorides are highly sensitive to humidity and oxygen, which is of particular importance when exclusively using their surfaces for catalytic applications. X-ray photoelectron spectroscopy (XPS) was measured on a customized spectrometer from SPECS GmbH with a Phoibos 150 hemispherical energy analyzer, a 1D-DLD detector and a non-monochromatic Mg radiation source (15 kV, 200 W, E = 1253 eV) applying the medium area lens mode. Survey scans and element-specific high-resolution scans were obtained with a pass energy of 50 and 20 eV, respectively. Measurements were performed under a vacuum of $< 1 \cdot 10^{-9}$ mbar using an electron flood gun. For the quasi in situ experiment, pristine $NbCl_5$ was first introduced into the measurement apparatus, fully characterized and subsequently transferred within the apparatus to a flow-type reaction chamber. Under N_2 flow (50 mL/min) the sample was heated with 5 °C/min from RT to 120 °C, before applying the reaction feed consisting of 2 vol.% C_2H_2 in N_2 (C_2H_2/N_2 1:49, 50 mL/min, WHSV ca. 30 000 $cm^3\ h^{-1}\ g_{cat}^{-1}$) for 1 h at 120 °C reaction temperature. After reaction the sample was cooled down at 5 °C/min under N_2 flow (50 mL/min) to RT and evacuated for 1 h before being transferred back to the measurement chamber for subsequent characterization. After the first measurement, the spent material was kept for 96 h in vacuum ($< 1 \cdot 10^{-9}$ mbar) inside the instrument before a second measurement, then shortly

removed from the instrument, reacted with air under ambient conditions for ca. 5 min and reintroduced into the instrument for a third measurement. This procedure was used to differentiate between low-valent niobium chloride species and Nb^{5+} bound to oxygen impurities instead of chlorine. As a safety warning, acetylene forms highly explosive acetylides upon contact with certain metals such as Cu that are often found in XPS instruments (e.g. sealings). The reaction chamber for this quasi in situ experiment did not contain any of those metals and a stainless steel-lined sample holder (316L) was used. Pristine $NbCl_5$ and Nb_2O_5 were measured and referenced to C 1s = 284.8 eV in order to extract the respective corrected values for Nb^{5+} $3d_{5/2}$ of 207.9 and 206.6 eV, respectively. All other measurements were then referenced to Nb^{5+} $3d_{5/2}$ = 207.9 eV to account for surface charging differences arising due to the expected significant differences in electric conductivity with the deposition of highly electron conducting polyacetylene in the spent samples. Fitting and deconvolution of the raw data was done with the CasaXPS software (version 2.3.26RP1.0)[2]. The gas chromatography with flame ionization detection or mass spectrometer as detector (GC-FID/-MS) for byproduct identification via gasbag samples or cold trap samples (at -50 °C dry ice/acetone) dissolved in MeOH collected at the reactor outlet during reaction or extracted from the carbon deposits of spent $NbCl_5$ catalyst (655 mg spent $NbCl_5$, 1.4 mL MeOH, 120 min ultrasonification, filtration over Whatman paper) was performed on a GC 7890B (Agilent, injector temperature 220 °C) equipped with an FID detector (at 350 °C) or on a Trace GC Ultra (Thermo Fischer Scientific, injector temperature 250 °C) directly coupled with a MS detector (Thermo Fischer Scientific, EI injector, ISQ Series or Q Exactive GC Orbitrap instrument). The gas (500 µl) or liquid (1.0 µl) was injected (split ratio 10:1 to 20:1) on the following columns with the respective temperature program depending on the sample composition: Rt-Msieve 5A column (for gas sample, 28 m, 0.53 mm ID, 50 µm df, 0.5 bar He, TCD instead of FID detector) at 30 °C (15 min isothermal, 8 °C/min ramp to 30 °C, 3 min isothermal), Rt-Q-Bond column (for gas sample, 30 m, 0.53 mm ID, 20 µm df, 0.5 bar He) at 30 °C (15 min isothermal, 8 °C/min ramp to 300 °C, 3 min isothermal), DB-1 column (for liquid sample, 30 m, 0.25 mm ID, 0.25 µm df, 0.6 bar H_2) at 50 °C (5 °C/min ramp to 350 °C), HP-5 MS column (for liquid sample, 30 m, 0.25 mm ID, 0.25 µm df, 0.6 bar H_2) at 50 °C (5 °C/min ramp to 320 °C, 3 min isothermal) and HP-Plot/Al_2O_3 column (for liquid sample, 27.5 m, 0.25 mm ID, 5.0 µm df, 0.8 bar H_2, 500 µl injection) at 40 °C (5 min isothermal, 5 °C/min ramp to 200 °C, 3 min isothermal). Thermogravimetric analysis coupled with mass spectrometry (TGA-MS) was done on a Netzsch Jupiter STA 449F3 instrument connected to a Netzsch Aëolos QMS 403D mass spectrometer (transfer line and injection system at 160 °C) in the temperature range from 45 to 1000 °C (25 °C/min, 10 mL/min Ar). Mass fragments in a range of 1-300 m/z were collected in the analog scan mode. Data analysis was done via the Netzsch Proteus – Thermal Analysis software (version 6.1.0). Nitrogen physisorption (N_2 physisorption) was conducted at -196 °C on a Micromeritics 3Flex. Prior to measurement the samples were degassed at 30 °C (5 °C/min, 1.5 h) in vacuum (< $1.33\cdot10^{-2}$ mbar). The surface area was determined using the Brunauer-Emmett-Teller (BET) method in the relative pressure range of 0.05 to 0.15 using the Micromeritics MicroActive software (version 5.02). The pore volume was calculated from the nitrogen quantity adsorbed at P/P^0 > 0.98 and the respective pore size distribution was estimated according to Barrett-Joyner-Halenda (BJH) algorithm on the adsorption isotherm (Harkins and Jura model with standard correction). Inductively coupled plasma optical emission spectroscopy (ICP-OES), ion chromatography (IC) and combustion analysis for the determination of the Nb, Cl, C and H content, respectively, were performed by Mikroanalytisches Labor Kolbe at Fraunhofer Umsicht in Oberhausen, Germany. ICP-OES measurements to determine the Nb content was conducted after a microwave digestion of the sample with a CEM MARS 6 unit on a Spectro Arcos ICP instrument. The Cl content was assessed on a Metrohm 930 Compact IC Flex Oven/SeS/PP/Deg after a combustion digestion of the sample using a Mitsubishi AQF-2100H unit. Combustion analysis for the C and H content was performed on an Elementar Vario Mikro CHNS analyzer. ICP-OES measurements were also performed in-house under ambient conditions on a Spectro Green FMX 46 Type 76004566 DSOI equipped with UVplus optics (range λ = 165 to 770 nm), ORCA (Optimized Rowland Circle Alignment), Crossflow and a Scotts chamber. For measurements, 10 to 20 mg of the sample was dissolved in 2 mL of an aqueous 40 wt.% HF solution at room temperature on a vibrating plate. Concentrations were obtained taking the average of three measurements with a previous calibration using a Nb standard from Analytichem. Powder X-ray diffraction (PXRD) measurements for qualitative phase identification were conducted on a Stoe STADI P transmission diffractometer equipped with a Mo radiation source (λ = 0.7093 Å), a primary Ge(111) monochromator (Mo $K\alpha_1$) and a position-sensitive Mythen1K detector. Diffraction patterns were acquired in the 2θ range of 2 to 50 ° with a step size of 0.015 ° and a collection time per step of 20 s. For the spent $NbCl_5$ catalyst, eight scans were recorded and summed up after measurement. Samples were prepared in glass capillaries (0.5 mm). The obtained diffraction patterns were qualitatively evaluated by comparison with entries from the ICDD PDF-2 2020 and the ICSD database. Raman spectroscopy was measured on a Renishaw inVia confocal Raman Microscope using an objective lens with 50x magnification and either an 532 nm (2.5 – 5 mW laser power) or 785 nm laser (0.03 - 15 mW laser power) at an exposure time of 10 s with 5 accumulations. Measurements were repeated on several spots of the sample to ensure the homogeneity of the sample. Attenuated total reflectance Fourier transform infrared spectroscopy (ATR FT-IR) was performed on an Agilent Cary 630 FTIR with diamond crystal. Spectra were obtained in a spectral range of 4000 to 650 cm^{-1} with a resolution of 4 cm^{-1} and 128 scan repetitions at room temperature. ^1H nuclear magnetic resonance spectroscopy (^1H- and ^1H-^1H-COSY NMR) was performed on a Bruker AV600neo (600 MHz) instrument with a Cryo-BBO probe head at 25 °C. Spectra were referenced to the deuterated solvent as an internal standard and the chemical shifts are reported in ppm. 50 mg of

spent $NbCl_5$ catalyst was ultrasonically dispersed for 30 min in 0.7 mL DMSO-d^6 and filtered with a syringe filter for the measurement of dissolvable non-volatile components in the carbon deposit. ^{1}H, ^{13}C, ^{29}Si and ^{93}Nb *solid-state* nuclear magnetic resonance spectroscopy (*ss*NMR) was recorded on a Bruker Avance III HD 500WB spectrometer using a double-bearing MAS probe (DVT BL4) at resonance frequencies of 500.192 MHz and 122 MHz for ^{1}H and ^{93}Nb, respectively. In case of the ^{93}Nb nucleus, the pulse calibration and chemical shift referencing were performed on a saturated $NbCl_5$ solution in acetonitrile. All NMR spectra were recorded at a temperature of 298 K (sensor temperature). Samples were packed into a 4-mm ZrO_2 rotor with a Vespel rotor cap inside a glovebox and kept in the dark at -20 °C when not in use. The ^{93}Nb Wideband Uniform Smooth Truncation-Quadrupole-Carr-Purcell Meiboom-Gill (WURST-QCPMG) spectra were recorded on a non-spinning sample with a sweep width of 5 MHz (40516.25 ppm). Up to 14 WURST-QCPMG sub-spectra were collected with variable carrier frequency from 10 000 ppm to -16 000 ppm in steps of 2000 ppm and co-added to in order to obtain the full span of the spectrum over a total spectral width of 8 MHz. From 128 to 20480 scans were accumulated at each frequency with a recycling delay of 0.7 s. The FIDs were recorded over a duration of 8 ms and included 40 QCPMG echo loops consisting of a 50 µs WURST pulse centered between 10 µs delays for ring-down spacing (receiver off) and a 130 µs acquisition delay (receiver on) resulting in the spikelet separation of 5000 Hz (full echo delay = 200 µs). The WURST pulse (pulse length = 50 µs, sweep width = 2 MHz, power index = 80) was generated within the shape tool module of Topspin 3.6. ^{1}H-decoupling was achieved with the tppm15 pulse train at 96 kHz. ^{29}Si (CP-)MAS *ss*NMR spectra of the pristine silica gel and $NbCl_x$-functionalized silica gel material were collected from 100 mg of material packed in a 4 mm rotor (+KelF cap) in a glovebox. For the direct polarization experiment of ^{29}Si, a 1.4 µs 30 ° excitation pulse was used with a recycling delay of 90s and a total of 768 scans were collected. The experimental conditions for the ^{29}Si CP-MAS NMR spectra were as follows: 10 kHz spinning rate, 25 s recycle delay, 512 scans, 4 ms contact time using a 70-100% ramp on ^{1}H optimized at ν_1 fields of approx. 74 kHz (^{1}H) and 59 kHz (^{29}Si), and 3.375 µs ^{1}H 90-pulse. ^{13}C CP-MAS NMR spectra were collected from 100 mg of material packed in a 4 mm rotor (+KelF cap) in a glovebox. The experimental conditions were as follows: 10 kHz spinning rate, 4 s recycle delay, 3000 scans, 3 ms contact time using a 70-100% ramp on ^{1}H optimized at ν_1 fields of approx. 50 kHz (^{1}H) and 56.5 kHz (^{13}C), and 3.25 µs ^{1}H 90-pulse. In both experiments, ^{1}H-decoupling was achieved with the tppm15 scheme at a field of 74 kHz. All spectra were processed in Topspin 3.6 and presented in MNova 15. To aid with the interpretation of the ^{93}Nb *ss*NMR spectra, we performed density functional theory (DFT) calculations using the CASTEP program with the GIPAW method on model structure geometries of different Nb_xCl_y complexes. The starting crystal cell structures for Nb_3Cl_8 (mp-29950), $NbCl_4$ (mp-31040) and $NbCl_5$ (mp-568483) were obtained from The Material Project (doi:10.1063/1.4812323). The calculations employed the PBE exchange-correlation functional, using a plane wave cutoff energy of 800 eV. The structures were optimized, relaxing all atomic positions while fixing the unit cell parameters to experimental values. The optimizations, which employed a LBFGS optimizer, were considered converged when the maximal residual force on an atom fell below 0.01 eV/Å and when the maximal atomic displacement with respect to the previous step was smaller than 0.0005 Å. DFT calculations of the isotropic shielding parameter σ_{iso}, the quadrupole coupling constant C_Q, and the asymmetry parameter η employed the gauge-including projector augmented wave (GIPAW) method as implemented in the CASTEP package. Calculations were performed on the DFT-optimized structures. In terms of exchange-correlation functional, pseudopotentials, cutoff energy, and k-meshes, the same settings as for the optimizations were used for the DFT-GIPAW calculations. Scanning electron microscopy (SEM) with energy dispersive X-ray spectroscopy (EDX) for bulk analysis and elemental mapping was performed on Hitachi TM3030 Plus table top scanning electron microscope (SEM) equipped with an Oxford Instruments Xplore Compact 30 detector at an acceleration voltage of 15 kV. Samples for SEM measurements were prepared under an argon shower by sprinkling dry specimen on a sticky carbon tape. High resolution (scanning) transmission electron microscopy (HR-(S)TEM) with energy dispersive X-ray spectroscopy (EDX) for bulk analysis and elemental mapping was conducted with a Thermo Scientific Talos F200x (scanning) transmission electron microscope equipped with a SuperX EDS system (Velox software) at an acceleration voltage of 200 kV with a brightfield (BF), darkfield (DF) and a high-angle annular darkfield detector (HAADF). Samples were prepared by sprinkling the dry powder onto a 400 mesh carbon lacey copper TEM-grid. X-ray emission spectroscopy (Nb $L\beta_2$) was measured on an in-house designed energy dispersive vacuum von Hamos spectrometer at the PINK tender X-ray beamline[3] at BESSY II. A bent Si(111) crystal with a bending radius of R = 250 mm dispersed incoming fluorescence radiation onto a GreatEyes CCD detector with a 26 µm x 26 µm pixel size (256 x 1024 pixels). The CCD detector accepted fluorescent radiation in a 2325-2382 eV energy window that corresponds to Bragg angles of θ = 58.3º-56.1º. The spectrometer resolution was approximately 1 eV. The excitation energy was set to 4000 eV using a multilayer monochromator. The beam size at the sample position was 30 µm x 500 µm fwhm (V x H) with a photon flux of ~$5e^{13}$ ph/s. In order to reduce radiation damage, the data were collected with continuous sample motion at a rate of 150 µm/s resulting in an effective sample exposure of 0.2 s per spot. Each pass took approximately 5 minutes. After that the scanning procedure was repeated. Typical measurement time per sample was 30 minutes or less. For the energy calibration procedure, Nb, Fe, and Pb foils were measured in the same configuration and calibrated to the Nb L[illegible]$_2$ and L[illegible]$_3$ lines at 2367.00 eV and 2334.70 eV, respectively, the Fe K[illegible]$_1$ line at 7057.98 eV, and the Pb M[illegible]$_1$ line at 2345.50 eV.[4] The energy of the Nb $L\beta_3$ line was calculated using the xraylib python library.[5] While the Nb and Pb spectra were collected using the Si(111) reflection, the Fe K[illegible] line was collected with the Si(333) reflection of the same

crystal without any rearrangements of the beamline optics or the spectrometer. Obtaining the Fe K[illegible] XES spectra required a higher excitation energy that was achieved with second order radiation at E = 8000 eV. To define the peak positions the Nb $L\beta_2$ and $L\beta_3$ XES spectra were fit with an asymmetric Lorentzian function, the Fe K[illegible] XES [illegible] spectrum was fit with four Voigt functions, and the Pb M[illegible] line was fit with one asymmetric Lorentzian (main peak) and three Voigt (small side peaks) functions. The energies were translated into Bragg angles and fit with a tangential function. All samples were stored and prepared in a N_2 filled glovebox and measured in the solid state at 30 K using helium as an exchange gas. The pure solids were ground to a fine powder and packed into 1 mm thick aluminum sample holders. The irradiated side of the cell was covered with 8 mm Kapton film and the backside of the cell was covered with 13 mm Kapton tape.

Catalyst testing

To handle acetylene in the pressurized state safely, several strict safety measures (e.g. explosion-safe cubicle, ventilation, steel with no other alloy components than carbon or highly alloyed steel, check valves, remote control to magnetic valves, purging possibilities, etc.) have to be followed. Reactions with pressurized acetylene should therefore not be performed in a common laboratory. For more detailed information on safety considerations when handling pressurized acetylene as well as more information on the reactor setup used in this study see the dissertation of I.-T. Trotus[6] or previously published studies[7]. Catalytic testing was performed using a plug-flow fixed bed reactor (stainless steel 316L, 8 mm i.d.) in a temperature range from 120 to 240 °C at 3 bar pressure. The reactor was heated by means of an external oven equipped with a thermocouple ($T_{heating}$). On stream temperature inside the reactor was measured by a thermocouple immersed into the catalyst bed. Pictures of the reactor setup can be seen in Figure S1. Hydrogen, nitrogen, ethylene, acetylene and methane were acquired from Air Liquide and used without further purification. Prior to compression by means of a compressor to 25 bar, acetylene was extracted and purified from acetone by a bed of activated alumina A and zeolite extrudates. Previously calibrated mass flow controllers ensured a controlled flow of gases totaling to a WHSV in the range of 1 700 to 10 300 $cm^3\ h^{-1}\ g_{cat}^{-1}$. The catalytic materials were tested by carefully supporting their powders on a bed of pre-dried quartz wool that was held in place by an underlying metal sieve and a quartz wool plug on top. The whole reactor preparation was performed inside an Ar filled glovebox. High-valent early transition metal chlorides are highly corrosive (especially upon contact with humidity) and partially strong oxidants. To avoid severe damage to reactor equipment, threads were covered with parafilm before alternated filling of small portions of quartz wool and catalytic material via a funnel (Figure S2-3). After preparation, the reactor was sealed and inertly transferred to the reactor setup and put under N_2 flow. All downstream piping of the reactor setup was heated between 150 and 200 °C to avoid product condensation. As an internal standard methane was added to the product stream downstream of the reactor. An online gas chromatograph (Agilent 7890B) with three sequential columns (Rxi-5Sil MS, RT-alumina BOND/Na_2SO_4, RT-Msieve 5A) from Restek and two FID and one TCD detector was used for qualitative and quantitative analysis of the feed and product gas stream composition. When heated and under reaction conditions high-valent early transition metal halogenides are prone to partially release halogen gases (F_2, Cl_2, Br_2 and I_2) and hydrogen halogenide gases (HF, HCl, HBr and HI) in small quantities that lead to severe corrosion upon contact with air humidity at leaks in the reactor setup (Figure S3). The reactor setup was therefore carefully checked for gas tightness with hydrogen prior to testing. GC columns were protected from corrosive gases and carbon dust particles using a 0.5 µm gas filter unit filled with Zn granulates (Figure S4). An additional heated backpressure regulator in parallel to the online GC allowed for the simultaneous sampling via gasbags and cold traps for offline GC-MS analysis (Figure S1). The gas flow of acetylene, ethylene, propene, 1-butene and 1,3-butadiene was determined via the peak area ratio to methane (set to a constant flow) applying response factors from previous calibration. For the determination of the gas flow of benzene, 1,3-cyclohexadiene and cyclohexene via the peak area ratio to methane, relative sensitivity values for H_2-FID detectors reported in literature[8] were applied. The total flow of other volatile minority species was calculated accordingly estimating an average relative sensitivity value of 1. Acetylene (or ethylene) conversion ($X_{C_2H_2}$) was calculated as the fraction of acetylene (or ethylene) exiting the reactor ($\dot{n}_{C_2H_2,\,out}$) relative to the acetylene (or ethylene) fed into the reactor ($\dot{n}_{C_2H_2,\,in}$) according to equation (1). The selectivity to benzene, ethylene, propene, 1-butene, 1,3-butadiene, 1,3-cyclohexadiene, cyclohexene and other volatiles (S_{C_x}) was determined using equation (2) based on the difference of molar flows in the feed ($\dot{n}_{C_x,\,in}$, when present) and product stream ($\dot{n}_{C_x,\,out}$) over the molar flow of converted acetylene considering the respective number of carbon atoms (a_{C_x}) of the compound. The carbon balance (CB) was calculated via equation (3) summing up the molar flows in the feed ($\dot{n}_{C_x,\,in}$) and product stream ($\dot{n}_{C_x,\,out}$) with the respective carbon atoms (a_{C_x}) of all present compounds. Several catalytic tests were repeated multiple times with the same and other batches for the bulk and supported materials (shown as scatters in the data) demonstrating good reproducibility. The average deviation between reproductions from the mean value was 3.6, 5.2 and 0.7% (abs.) or lower for the acetylene conversion, selectivity to benzene and other volatiles, respectively.

(1) $$X_{C_2H_2} = 1 - \frac{\dot{n}_{C_2H_2,out}}{\dot{n}_{C_2H_2,in}}$$

(2) $$S_{C_x} = \frac{\left(\dot{n}_{C_x,out}-\dot{n}_{C_x,in}\right)\cdot\left({}^{a_{C_x}}/_{2}\right)}{\dot{n}_{C_2H_2,in}-\dot{n}_{C_2H_2,out}}$$

(3) $$CB = \frac{\sum_{x=1}^{6}\left(a_{C_x}\cdot\dot{n}_{C_x,out}\right)}{\sum_{x=1}^{6}\left(a_{C_x}\cdot\dot{n}_{C_x,in}\right)}$$

TABLES

Table S1: ICP-OES (Nb content, N = 2), IC (Cl content) measurements and the determined atomic Nb/Cl ratio of the pristine and spent $NbCl_5$ catalyst after the gas-phase cyclotrimerization of acetylene to benzene (C_2H_2/N_2 1:10, 150 °C, 3 bar, WHSV ca. 6 600 $cm^3\ h^{-1}\ g_{cat}^{-1}$).

Element	Pristine $NbCl_5$ wt.%	Spent $NbCl_5$ wt.%
Nb	36 ± 2	21 ± 7
Cl	65	20
Nb / Cl (atomic)	1 : (4.7 ± 0.3)	1 : (3 ± 1)

Table S2: Combustion analysis and the determined atomic C/H ratio of spent $NbCl_5$ catalyst after the gas-phase cyclotrimerization of acetylene to benzene (C_2H_2/N_2 1:10, 150 °C, 3 bar, WHSV ca. 6 600 $cm^3\ h^{-1}\ g_{cat}^{-1}$).

Element	Spent $NbCl_5$ wt.%
C	40.9
H	3.4
C / H (atomic)	1 : 1

Table S3: SEM-EDX of the pristine and spent $NbCl_5$ catalyst after the gas-phase cyclotrimerization of acetylene to benzene (C_2H_2/N_2 1:10, 150 °C, 3 bar, WHSV ca. 6 600 $cm^3\ h^{-1}\ g_{cat}^{-1}$). The carbon content might be overestimated due to the preparation on a sticky carbon pad.

Element	Pristine $NbCl_5$ wt.% (at.%)	Spent $NbCl_5$ wt.% (N = 2) (at.%)
Nb	40.1 (12.8)	14 ± 1 (2.4 ± 0.1)
Cl	31.8 (26.5)	10.4 ± 0.3 (4.5 ± 0.1)
C	14.5 (35.7)	65 ± 3 (84 ± 4)
Si	-	2 ± 2 (1.0 ± 0.9)
O	13.6 (25.0)	8 ± 3 (8 ± 3)

Table S4: TEM-EDX of the pristine and spent $NbCl_5$ catalyst after the gas-phase cyclotrimerization of acetylene to benzene (C_2H_2/N_2 1:10, 150 °C, 3 bar, WHSV ca. 6 600 $cm^3\ h^{-1}\ g_{cat}^{-1}$). The carbon content might be overestimated due to the preparation on a lacey carbon supported copper grid.

Element	Pristine $NbCl_5$ wt.% (N = 7) (at.-%)	Spent $NbCl_5$ wt.% (N = 6) (at.-%)
Nb	40 ± 2 (18 ± 2)	13 ± 4 (2 ± 1)
Cl	52 ± 5 (60 ± 10)	15 ± 6 (7 ± 3)
C	3 ± 2 (9 ± 6)	70 ± 9 (89 ± 4)
Si	0.1 ± 0.1 (0.1 ± 0.2)	0.1 ± 0.1 (0.1 ± 0.1)
O	2 ± 1 (5 ± 2)	2 ± 1 (2 ± 1)

Table S5: Measured weight loadings of niobium (N = 2), chloride and respective total loading of $NbCl_x$ species and the molar Cl to Nb ratio measured via ICP-OES (Nb) and IC (Cl) for the $NbCl_x$-functionalized silica gels with different intended Nb/OH_{surf} ratios.

Nb/OH_{surf} ratio (synthesis)	Nb (wt.%)	Cl (wt.%)	$NbCl_x$ (wt.%)	Cl/Nb ratio (molar)
**0.1**	2.6 ± 0.6	2.8	5.4 ± 0.6	3.0 ± 0.8
**0.3**	4.9 ± 0.7	5.8	10.7 ± 0.7	3.3 ± 0.4
**0.6**	10 ± 2	11.2	21 ± 2	3.2 ± 0.6
**1.1**	14 ± 1	19.4	33 ± 1	3.6 ± 0.3

Table S6: BET surface area, pore volume and BJH average pore size (adsorption) from N_2 physisorption data of the pristine silica gel and the $NbCl_x$-functionalized silica gels.

$NbCl_x$ (wt.%)	BET surface area (m^2/g)	Pore volume (cm^3/g)	BJH (ads.) average pore size (nm)
**0**	297	1.09	14.4
**5**	274	0.98	13.9
**11**	272	0.92	13.1
**21**	230	0.75	11.8
**33**	167	0.57	12.2

Table S7: BET surface area, pore volume and BJH average pore size (adsorption) from N_2 physisorption data of the pristine silica gel and the spent $NbCl_x$-functionalized silica gels after the gas-phase cyclotrimerization of acetylene to benzene (C_2H_2/N_2 1:10, 180 °C, 3 bar, WHSV ca. 66 000 – 80 000 $cm^3\ h^{-1}\ g_{Nb}^{-1}$) with the percentage of loss in comparison to the pristine state before catalysis (Table S6) in brackets.

$NbCl_x$ (wt.%)	BET surface area (m^2/g)	Pore volume (cm^3/g)	BJH (ads.) average pore size (nm)
**0**	297	1.09	14.4
**5**	260 (-5%)	0.97 (-1%)	13.9 (-0%)
**11**	228 (-16%)	0.75 (-18%)	12.6 (-4%)
**21**	83 (-64%)	0.19 (-75%)	8.6 (-27%)
**33**	33 (-80%)	0.08 (-86%)	9.6 (-21%)

Table S8: Weight loading of Nb, Cl, C and H and the molar Cl/Nb and C/H ratio from ICP-OES (Nb, N = 2), IC (Cl) measurements and combustion analysis (C, H) of the pristine and spent 21 wt.% $NbCl_x$-silica gel catalyst after the gas-phase cyclotrimerization of acetylene to benzene (C_2H_2/N_2 1:10, 180 °C, 3 bar, WHSV ca. 66 000 $cm^3\ h^{-1}\ g_{Nb}^{-1}$).

Element	Pristine (wt.%)	Spent (wt.%)
**Nb**	10 ± 2	8 ± 3
**Cl**	11.2	5.6
**Cl/Nb (molar)**	3.2 ± 0.6	2.0 ± 0.7
**C**	-	36.0
**H**	-	3.0
**C/H (molar)**	-	1.0

Table S9: Literatur comparison of 21 wt.% $NbCl_x$-silica gel catalyst with the so far best performing 1 wt.% Ni/silica gel catalyst reported by Boudjahem and coworkers[9] in the gas-phase cyclotrimerization of acetylene to benzene with regard to productivity, space-time-yield and turnover frequency.

**Catalyst / Conditions**	**Productivity (g_{C6H6} g_{cat}^{-1} h^{-1})**	**Space-Time-Yield (g_{C6H6} cm_{cat}^{-3} h^{-1})**	**Turnover Frequency (mol_{C6H6} mol_{M}^{-1} h^{-1})**
**1 wt.% Ni/silica gel**<br>($C_2H_2/H_2/H_2O/He$ 1:4:0.1:15, 40 °C, 1 atm, WHSV 30 000 cm^3 g_{cat}^{-1} h^{-1})<br>*Appl. Cat. Gen.* **2003**, 250, 1, 49-64.	0.08	-	5.68
**21 wt.% $NbCl_x$-silica gel**<br>(C_2H_2/N_2 1:10, 180 °C, 3 bar,<br>WHSV 6 600 cm^3 h^{-1} g_{cat}^{-1}<br>(this work)	1.60	0.53	19.02

FIGURES

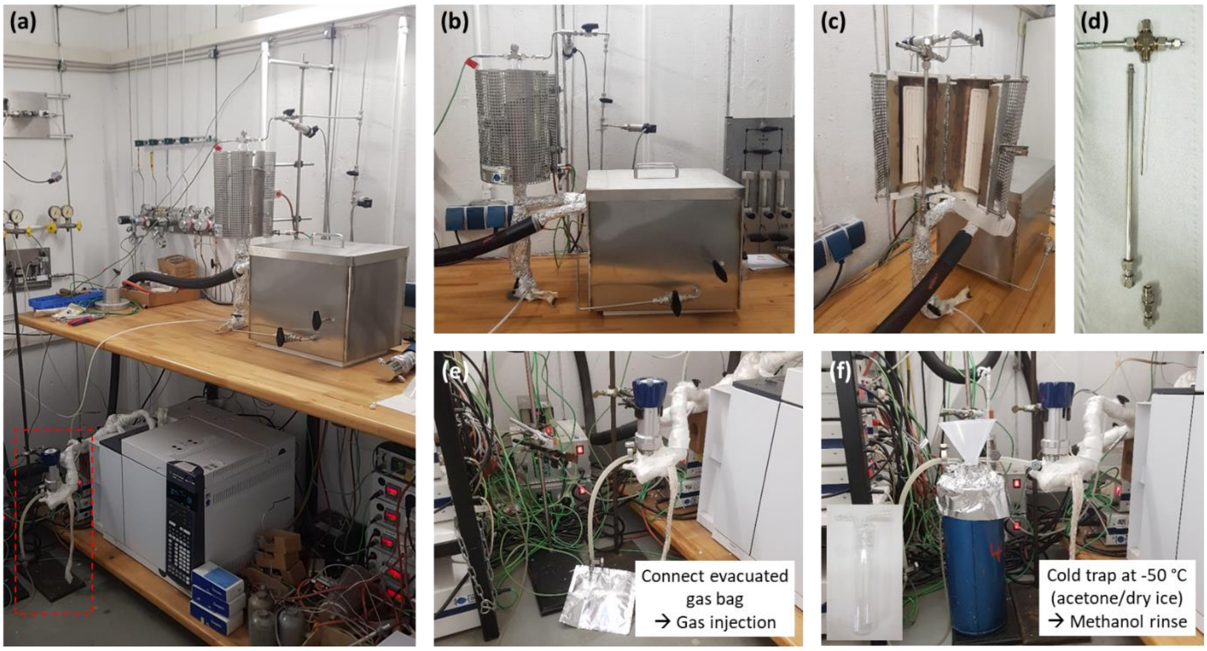

Figure S1: (a) Overview of the acetylene flow reactor linked to an online GC underneath. Red dashed box shows the additional heated backpressure regulator in parallel to GC for simultaneous sampling with gasbags and cold traps for offline GC-MS product analysis. (b) Flow reactor with oven and heated box with backpressure regulator. (c) Opened reactor oven. (d) Disassembled plug-flow fixed bed reactor. (e) Gasbag connected to an additional backpressure regulated exit for sampling. (f) Cold trap (-50 °C, acetone/dry ice) linked to additional backpressure regulated exit for sampling. Inlet shows the glass trap immersed in the Dewar.

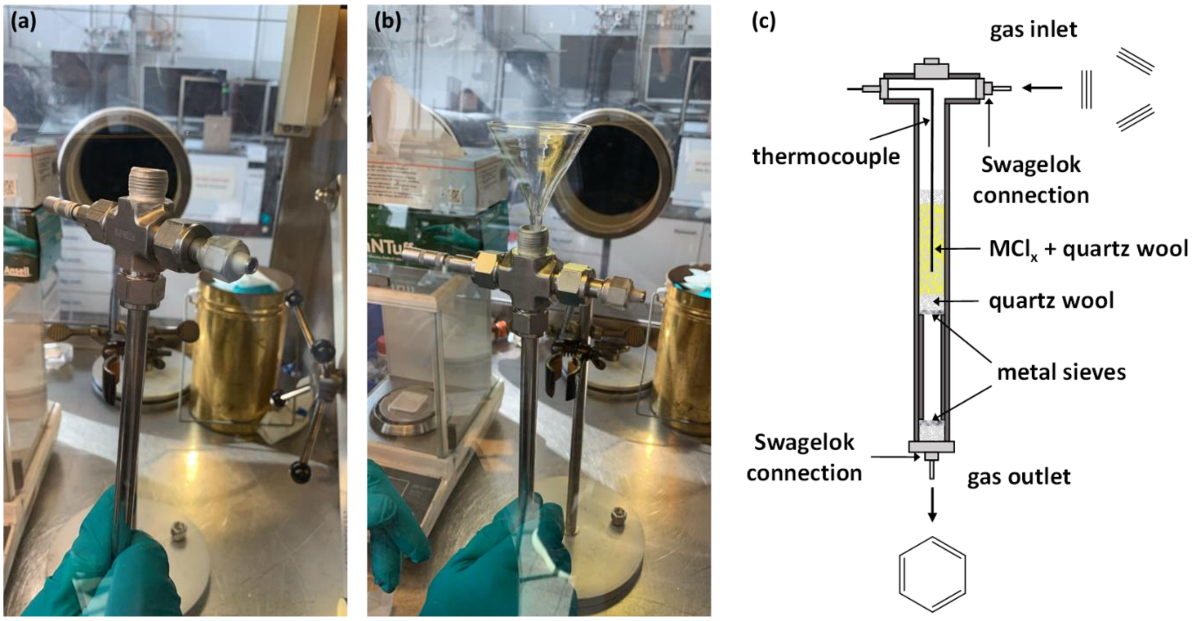

Figure S2: (a) Covering of reactor threads with parafilm to avoid contact with catalytic materials during filling in Ar filled glovebox. (b) Filling of portions of catalytic material via a funnel to avoid contact with thread. (c) Scheme of the prepared reactor for catalytic testing.

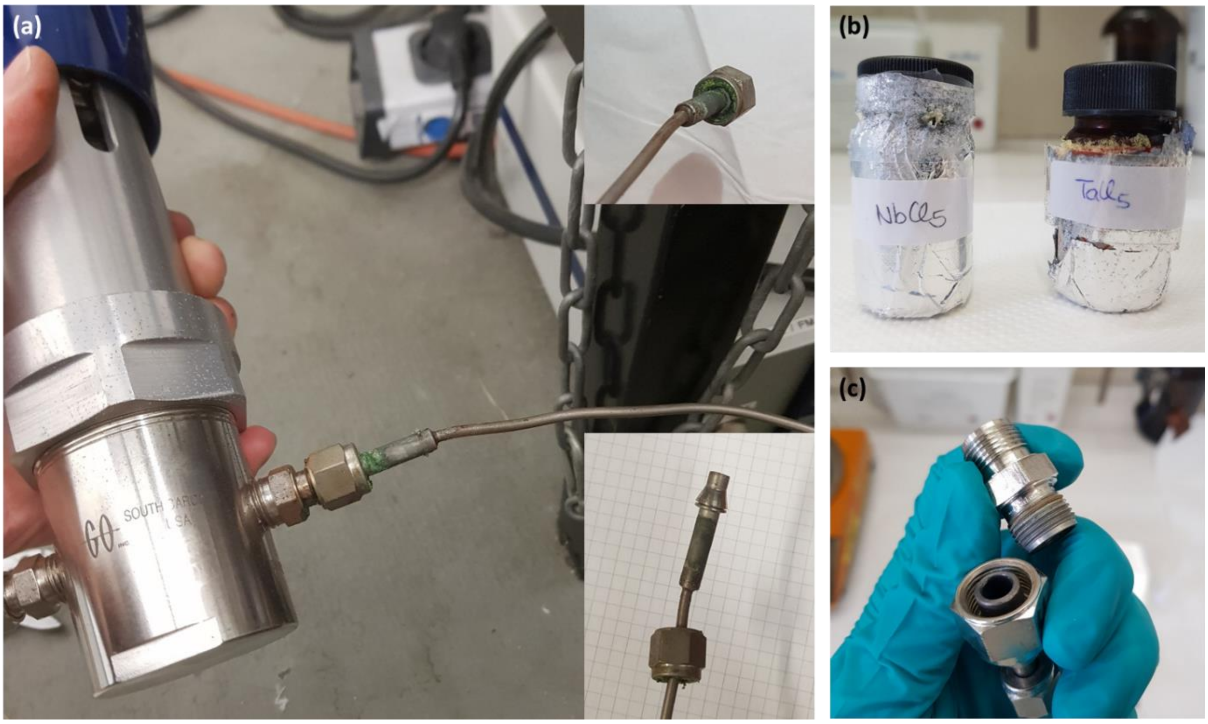

Figure S3: (a) Corroded thread and gas line due to a gas leak. Inlets showing corrosion only at the point where the gas left the (inert gas filled) interior and got into contact with air humidity. (b) Closed vessels of $NbCl_5$ and $TaCl_5$ wrapped in aluminum foil when stored incorrectly outside the glovebox demonstrating corrosion of the aluminum foil upon on contact with air humidity. (c) Corroded thread at the reactor outlet part due to a gas leak at this point.

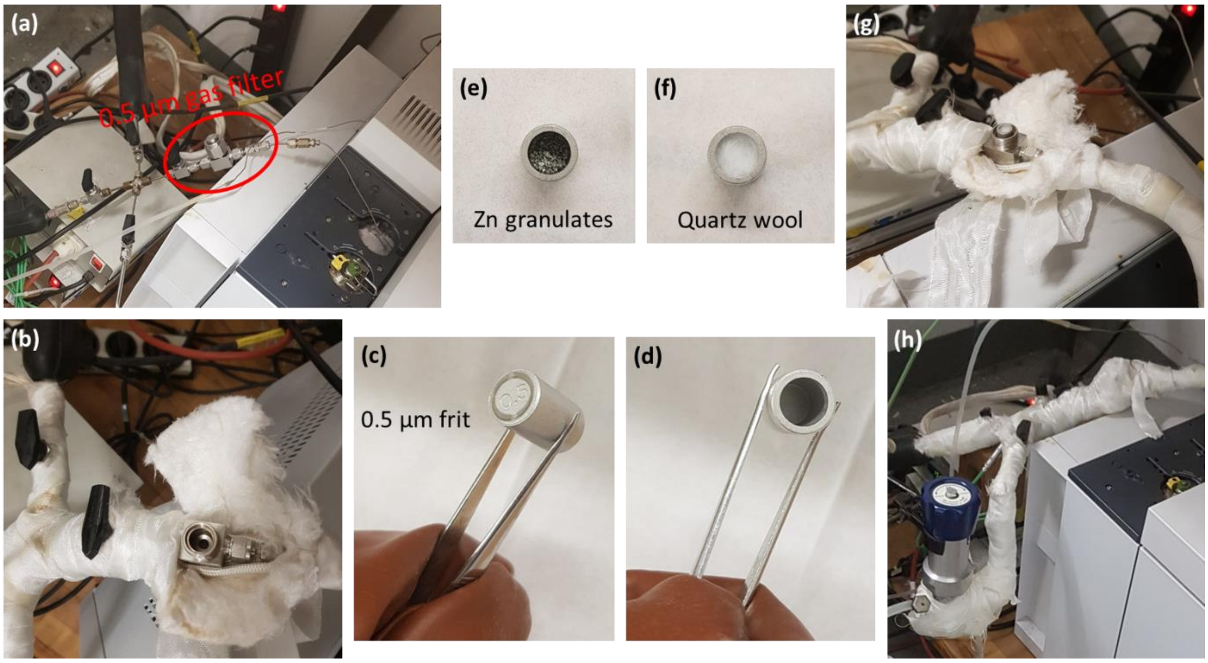

Figure S4: (a-h) Installation of a gas particle filter (Ø 0.5 µm) filled with Zn granulates and quartz wool in the heated downstream part of the reactor setup before the online GC to protect columns from corrosive gases and carbon dust particles.

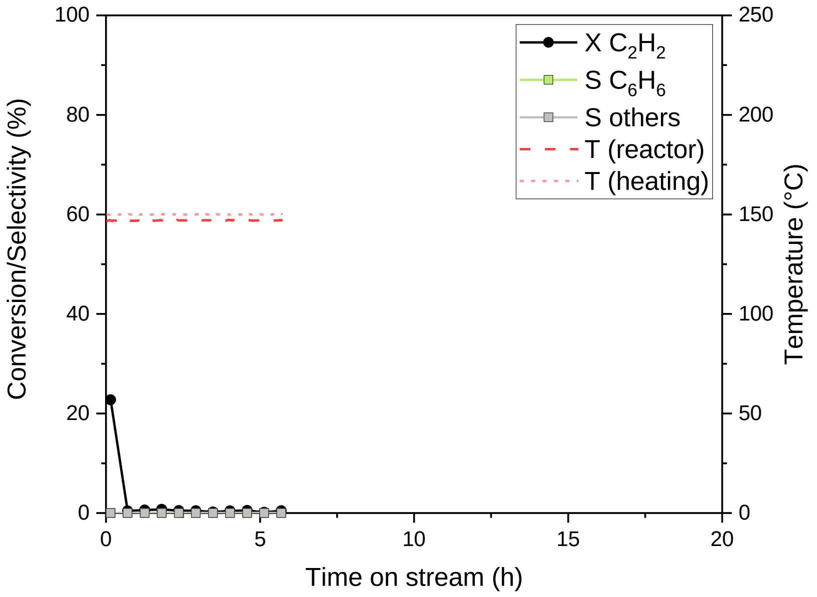

Figure S5: Selectivity to benzene and other volatiles (“others”), acetylene conversion and temperature profiles for $ZrCl_4$ in the gas-phase cyclotrimerization of acetylene to benzene (C_2H_2/N_2 1:10, 150 °C, 3 bar, WHSV 6 600 cm^3 h^{-1} $g_{cat}{}^{-1}$).

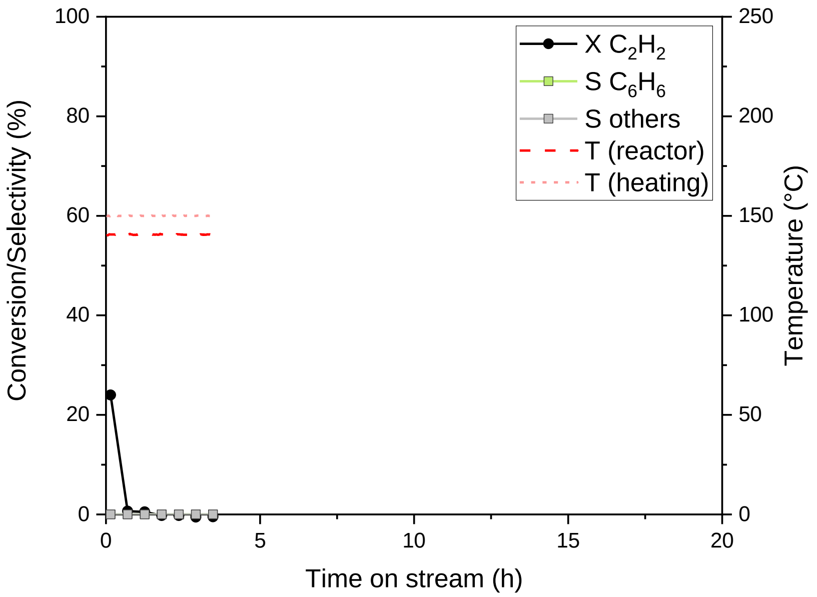

Figure S6: Selectivity to benzene and other volatiles (“others”), acetylene conversion and temperature profiles for $HfCl_4$ in the gas-phase cyclotrimerization of acetylene to benzene (C_2H_2/N_2 1:10, 150 °C, 3 bar, WHSV 6 600 cm^3 h^{-1} $g_{cat}{}^{-1}$).

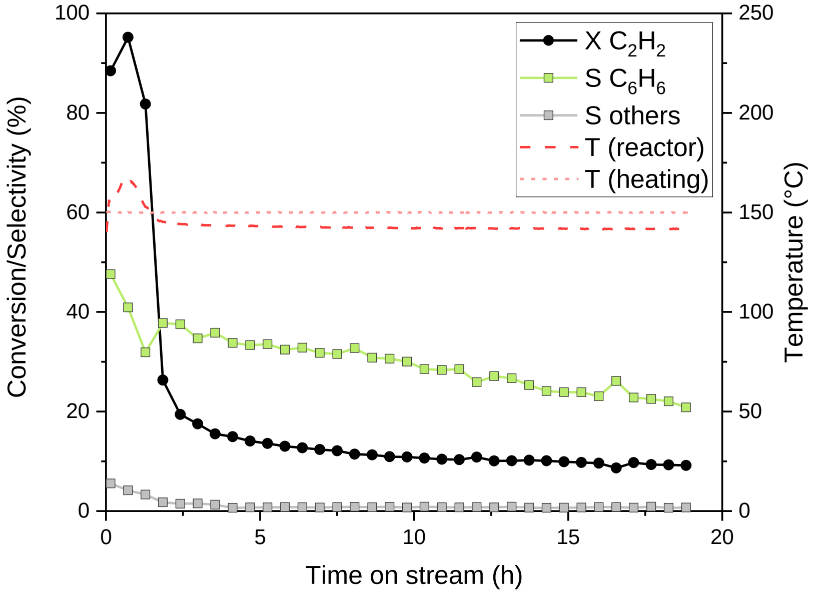

Figure S7: Selectivity to benzene and other volatiles ("others"), acetylene conversion and temperature profiles for $NbCl_5$ in the gas-phase cyclotrimerization of acetylene to benzene (C_2H_2/N_2 1:10, 150 °C, 3 bar, WHSV 6 600 $cm^3\ h^{-1}\ g_{cat}{}^{-1}$).

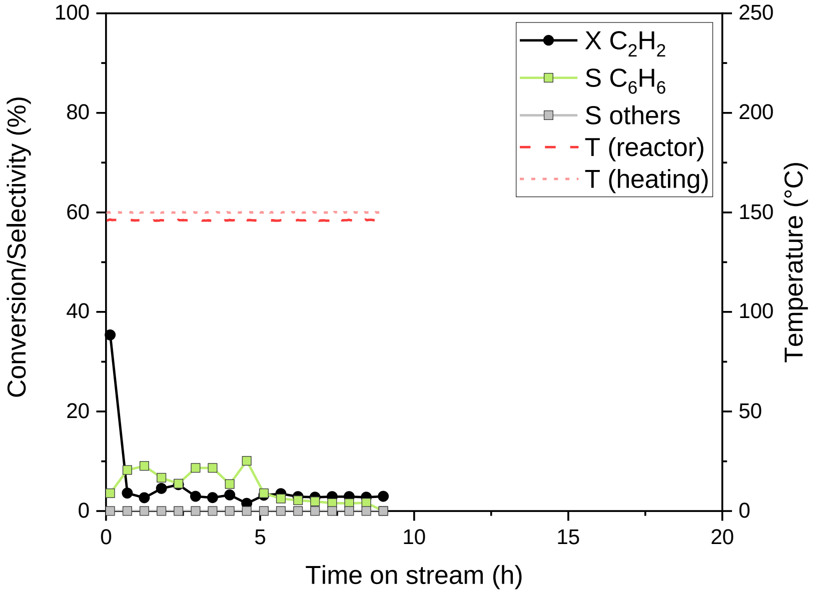

Figure S8: Selectivity to benzene and other volatiles ("others"), acetylene conversion and temperature profiles for $TaCl_5$ in the gas-phase cyclotrimerization of acetylene to benzene (C_2H_2/N_2 1:10, 150 °C, 3-4 bar, WHSV 6 600 $cm^3\ h^{-1}\ g_{cat}{}^{-1}$).

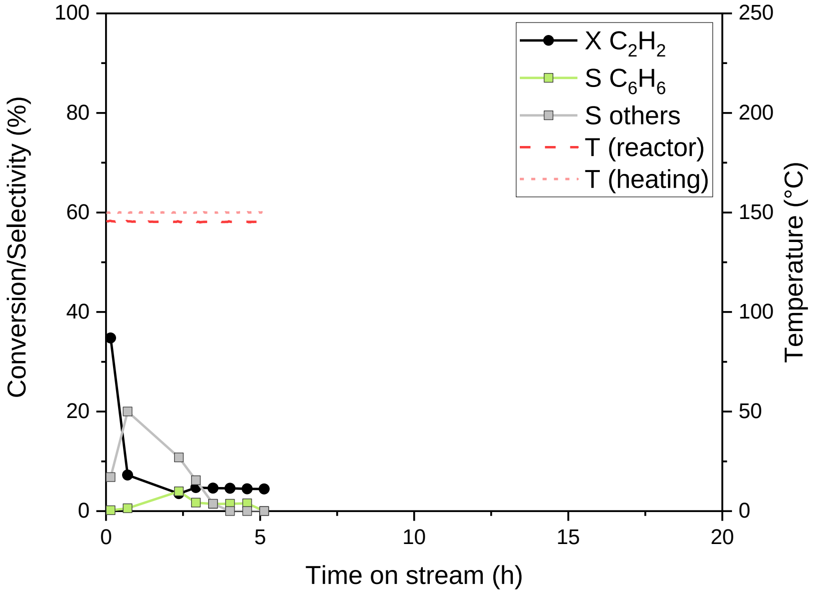

Figure S9: Selectivity to benzene and other volatiles ("others"), acetylene conversion and temperature profiles for $MoCl_5$ in the gas-phase cyclotrimerization of acetylene to benzene (C_2H_2/N_2 1:10, 150 °C, 3 bar, WHSV 6 600 cm^3 h^{-1} g_{cat} ^{1}).

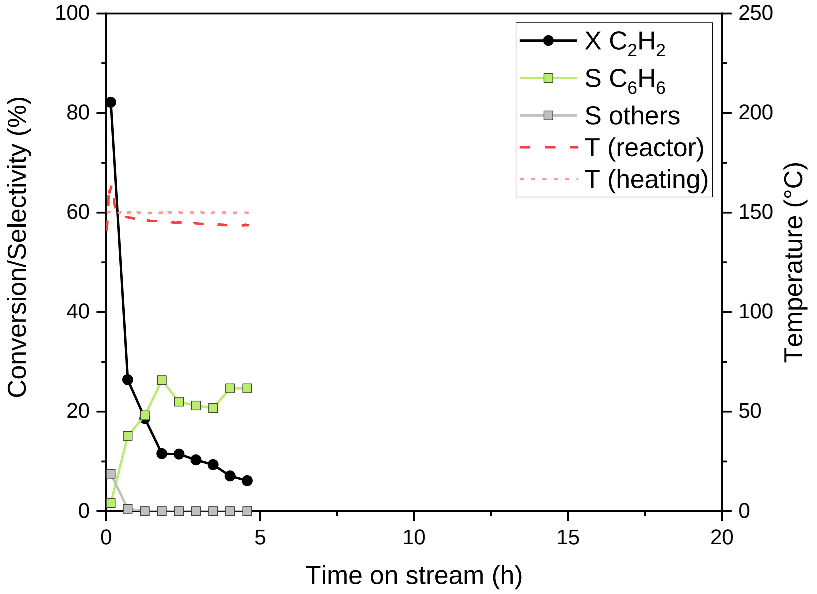

Figure S10: Selectivity to benzene and other volatiles ("others"), acetylene conversion and temperature profiles for WCl_6 in the gas-phase cyclotrimerization of acetylene to benzene (C_2H_2/N_2 1:10, 150 °C, 3 bar, WHSV 6 600 cm^3 h^{-1} g_{cat} $^{-1}$).

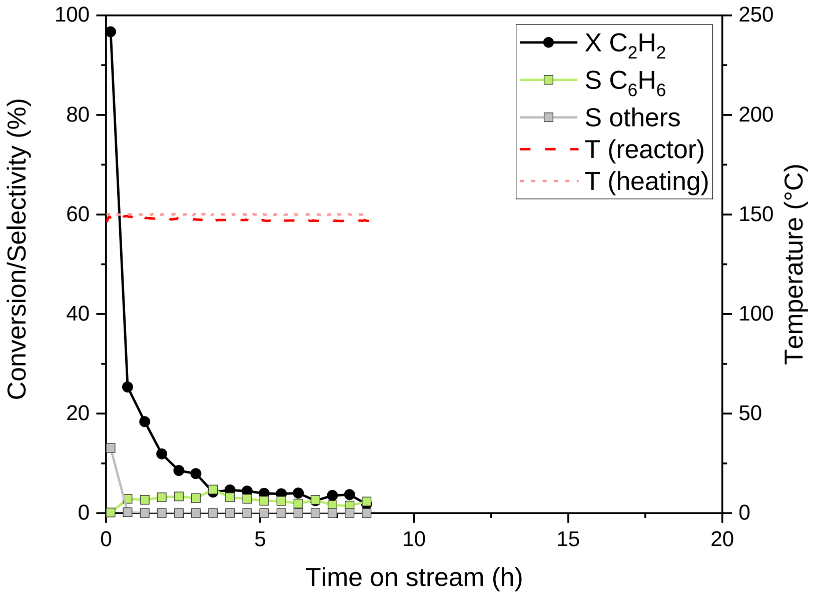

Figure S11: Selectivity to benzene and other volatiles ("others"), acetylene conversion and temperature profiles for $ReCl_5$ in the gas-phase cyclotrimerization of acetylene to benzene (C_2H_2/N_2 1:10, 150 °C, 3 bar, WHSV 6 600 cm^3 h^{-1} g_{cat}^{-1}).

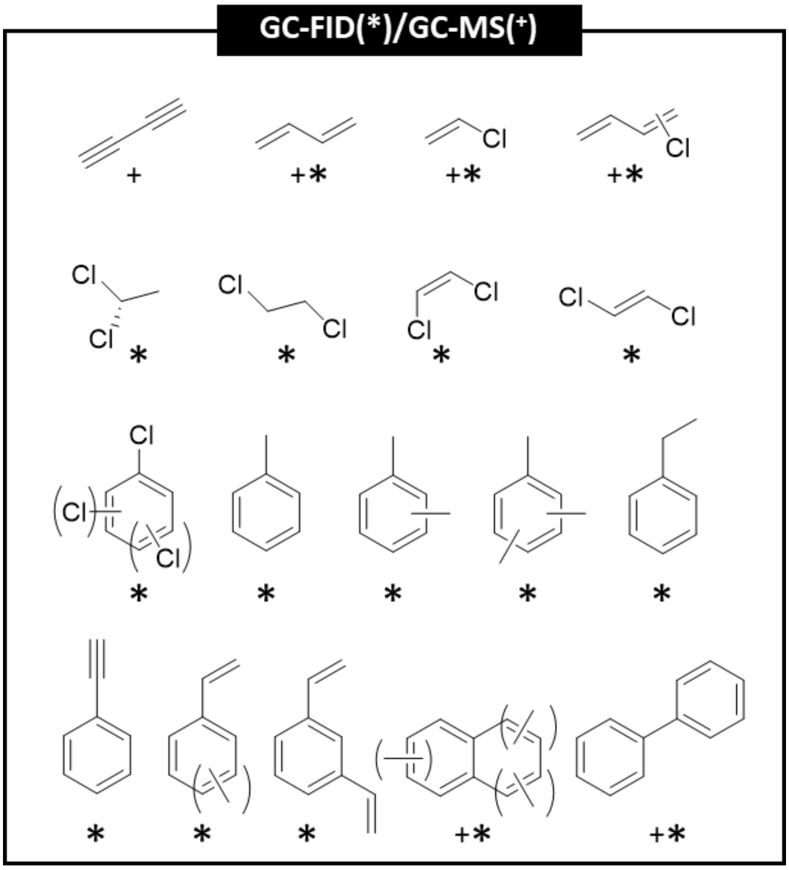

Figure S12: Volatile Byproducts ("other") assumed/identified via GC-FID (*) injecting reference compounds or via GC-MS (+) collecting gasbag samples or extracting from cold-traps.

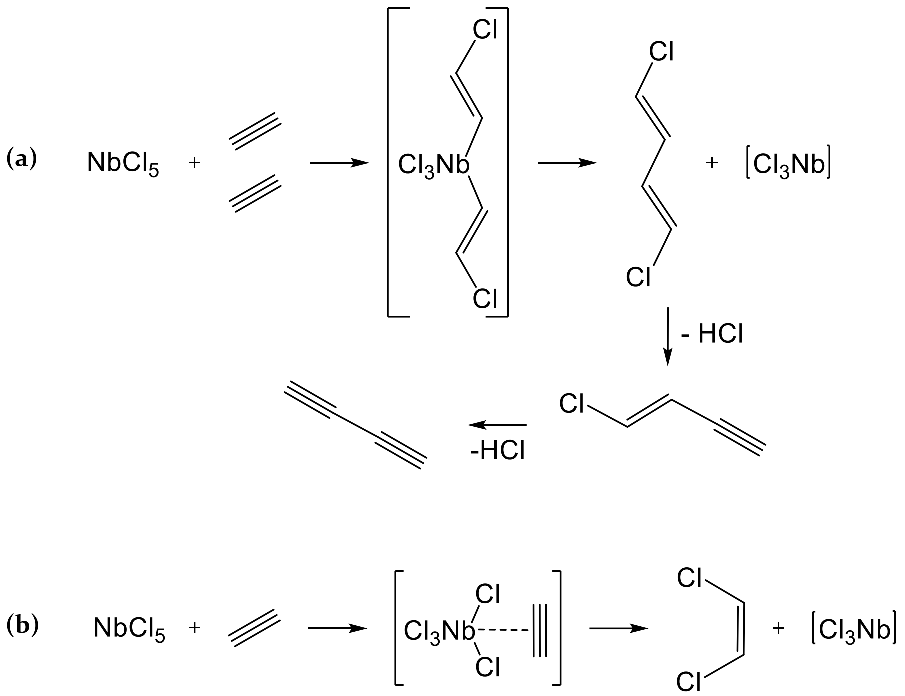

Figure S13: Proposed mechanism based on literature[10] on liquid media reactivity for the in situ reduction of $NbCl_5$ with acetylene via (a) C-C coupling to (chlorinated) C_4 compounds (e.g. diacetylene) and/or (b) direct chlorination of acetylene to cis-1,2-dichloroethylene.

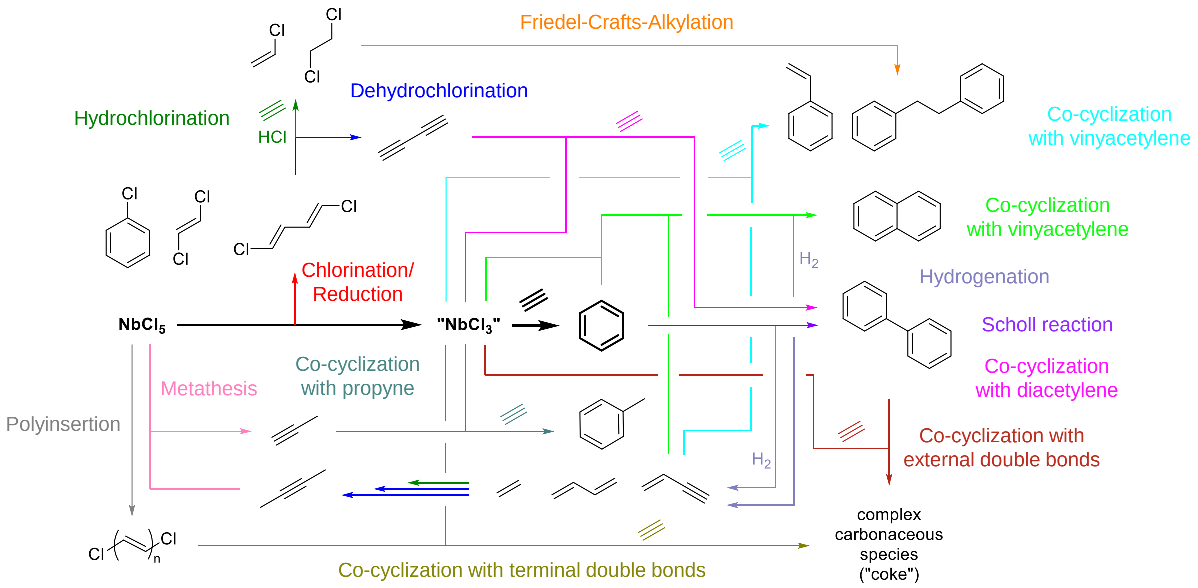

Figure S14: Possible reaction pathways to various detected volatile and non-volatile byproducts during the gas-phase cyclotrimerization reaction of acetylene to benzene on strong redox-active Lewis acids such as $NbCl_5$.

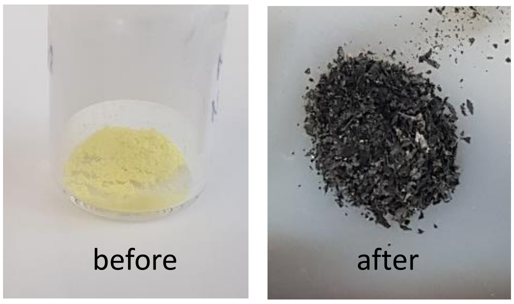

Figure S15: $NbCl_5$ catalyst before and after reaction with acetylene. Grinding the spent $NbCl_5$ catalysts reveals a surface contamination with a black deposit during reaction with unreacted yellow material underneath.

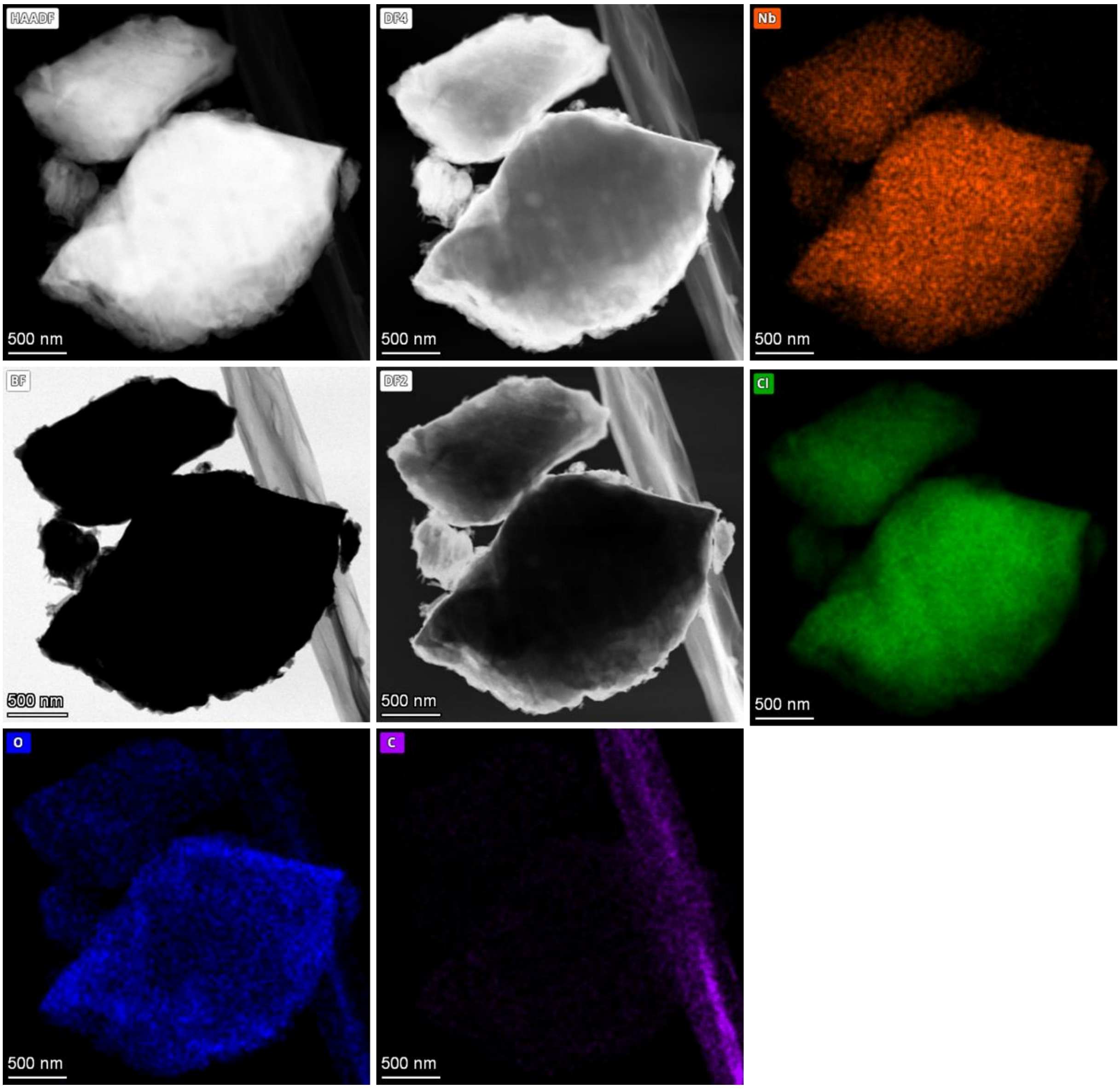

Figure 16: Transmission electron microscopy images with elemental mapping of the pristine $NbCl_5$ catalyst most likely revealing contact with some air/humidity impurities (that have reacted on the surface to NbO_x species) during inert transfer to the transmission electron microscope.

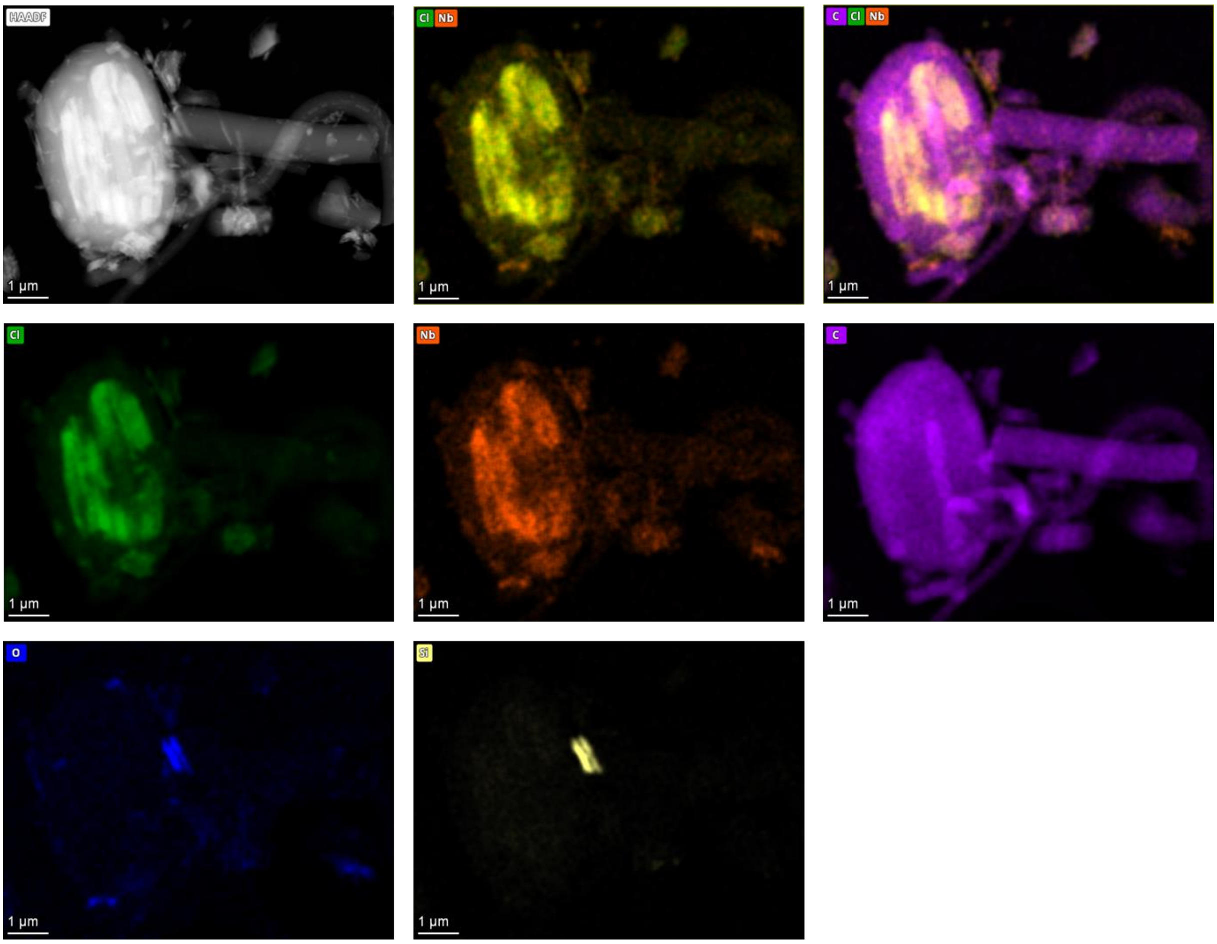

Figure 17: Transmission electron microscopy images with elemental mapping of the spent $NbCl_5$ catalyst after the gas-phase cyclotrimerization of acetylene to benzene (C_2H_2/N_2 1:10, 150 °C, 3 bar, WHSV ca. 6 600 $cm^3\ h^{-1}\ g_{cat}^{-1}$) summed up in Figure 1b in the manuscript.

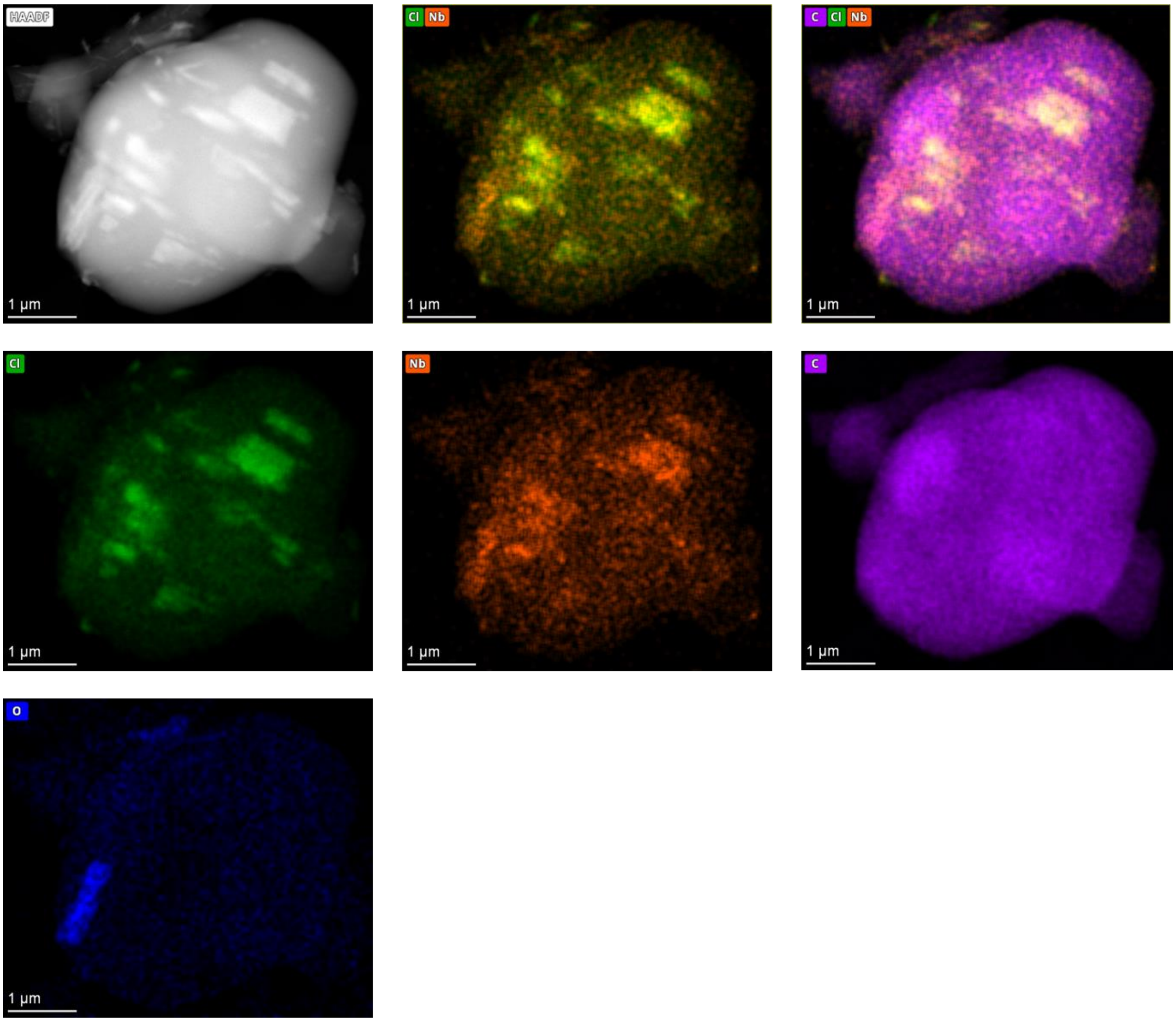

Figure 18: Transmission electron microscopy images with elemental mapping of the spent $NbCl_5$ catalyst after the gas-phase cyclotrimerization of acetylene to benzene (C_2H_2/N_2 1:10, 150 °C, 3 bar, WHSV ca. 6 600 $cm^3\ h^{-1}\ g_{cat}^{-1}$) showing the encapsulation with carbon.

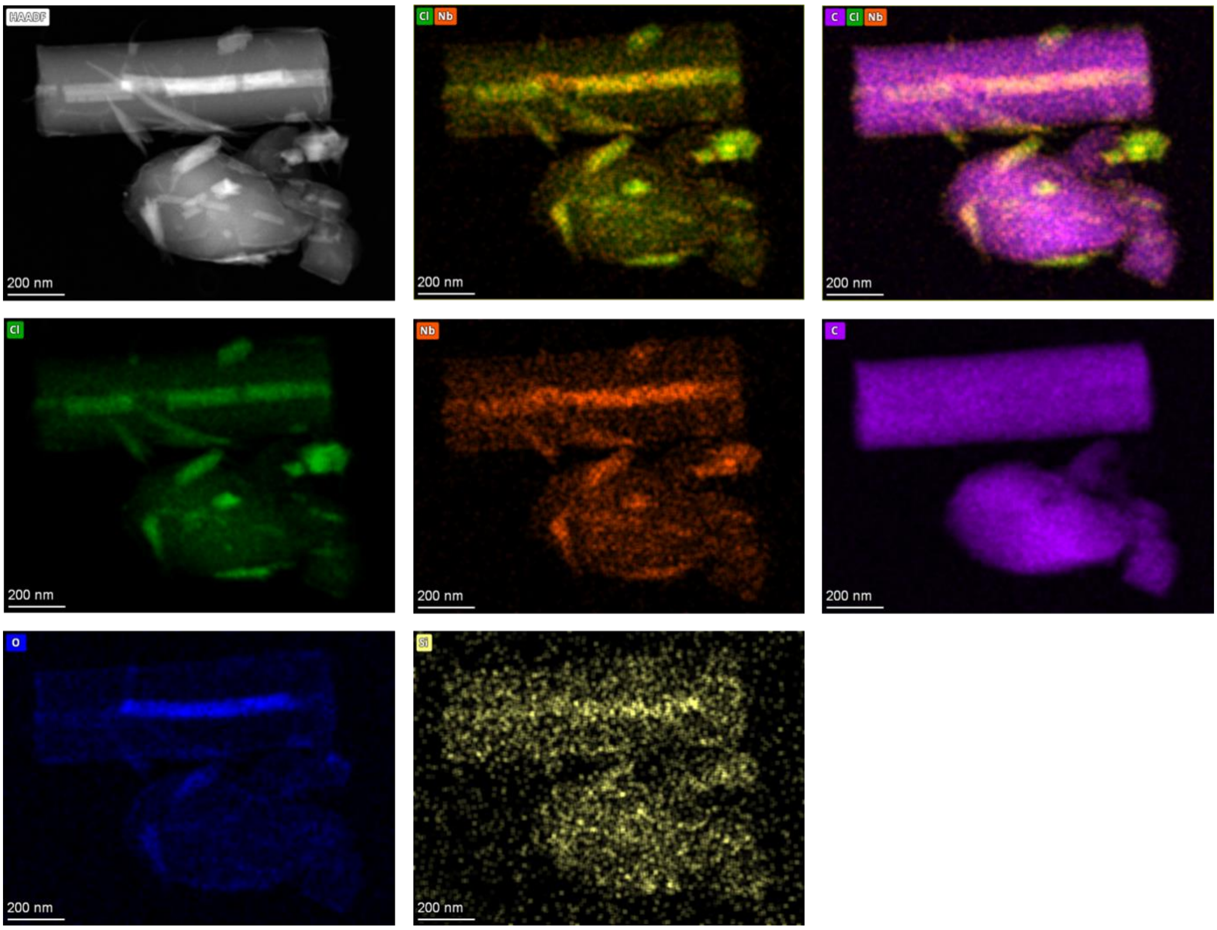

Figure 19: Transmission electron microscopy images with elemental mapping of the spent $NbCl_5$ catalyst after the gas-phase cyclotrimerization of acetylene to benzene (C_2H_2/N_2 1:10, 150 °C, 3 bar, WHSV ca. 6 600 $cm^3\ h^{-1}\ g_{cat}^{-1}$) showing the encapsulation with carbon.

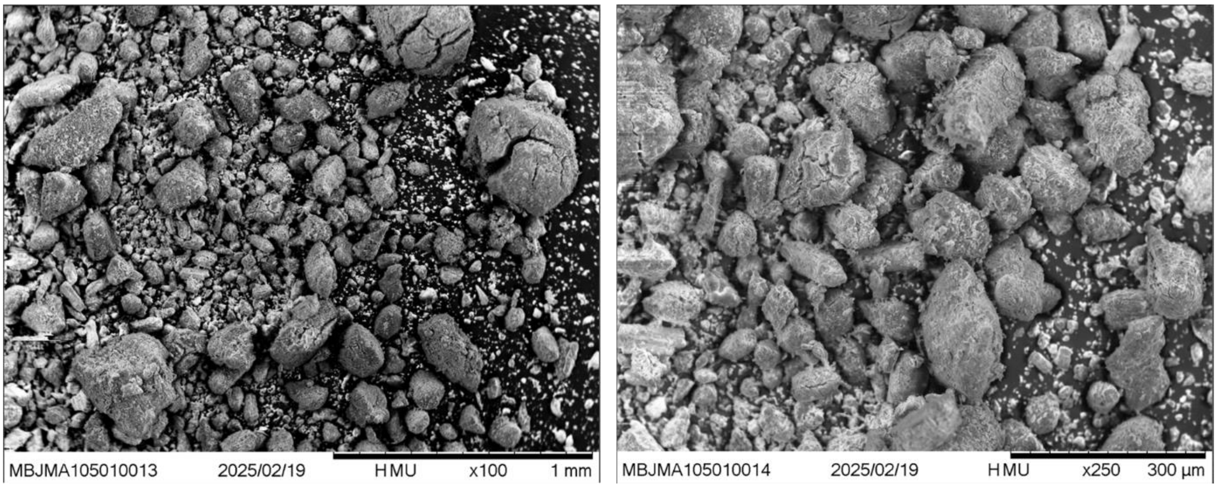

Figure 20: Scanning electron microscopy images of the pristine $NbCl_5$ catalyst.

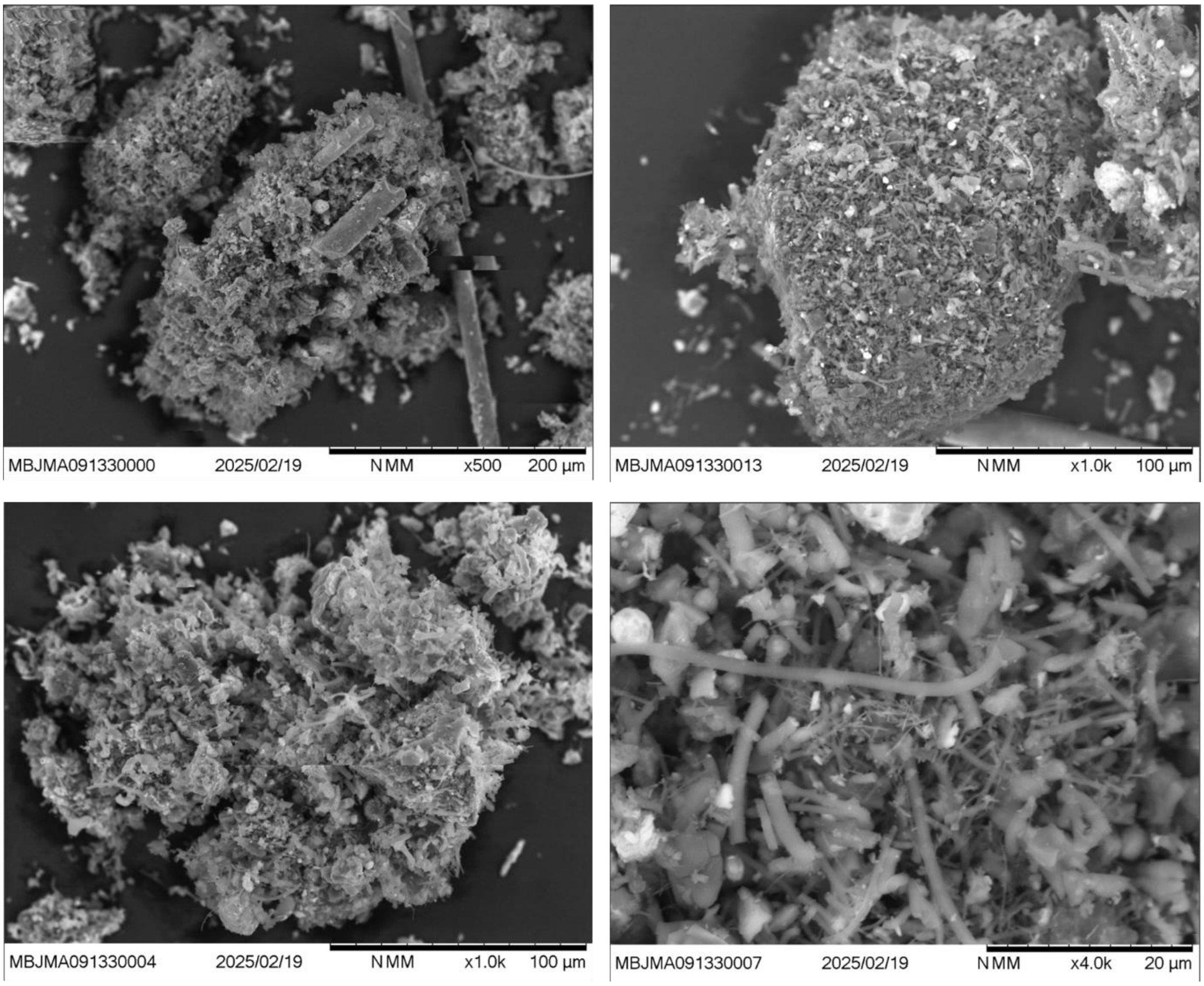

Figure 21: Scanning electron microscopy images of the spent $NbCl_5$ catalyst after the gas-phase cyclotrimerization of acetylene to benzene (C_2H_2/N_2 1:10, 150 °C, 3 bar, WHSV ca. 6 600 $cm^3\ h^{-1}\ g_{cat}^{-1}$) showing the plate- and fiber-like carbon growths on the surface of the catalyst.

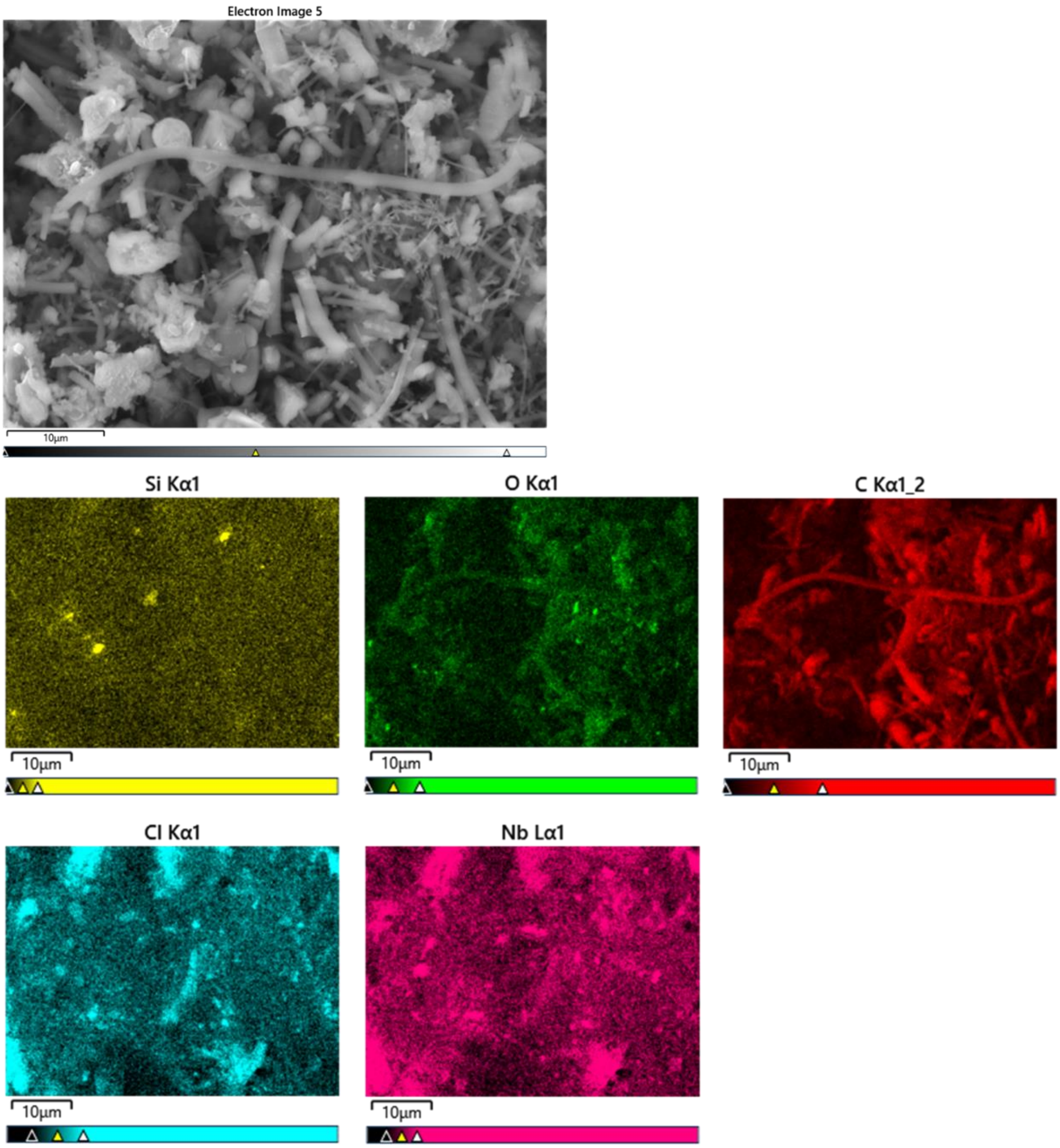

Figure 22: Scanning electron microscopy images with elemental mapping of the spent $NbCl_5$ catalyst after the gas-phase cyclotrimerization of acetylene to benzene (C_2H_2/N_2 1:10, 150 °C, 3 bar, WHSV ca. 6 600 $cm^3\ h^{-1}\ g_{cat}^{-1}$) showing the plate- and fiber-like carbon growths on the surface of the catalyst.

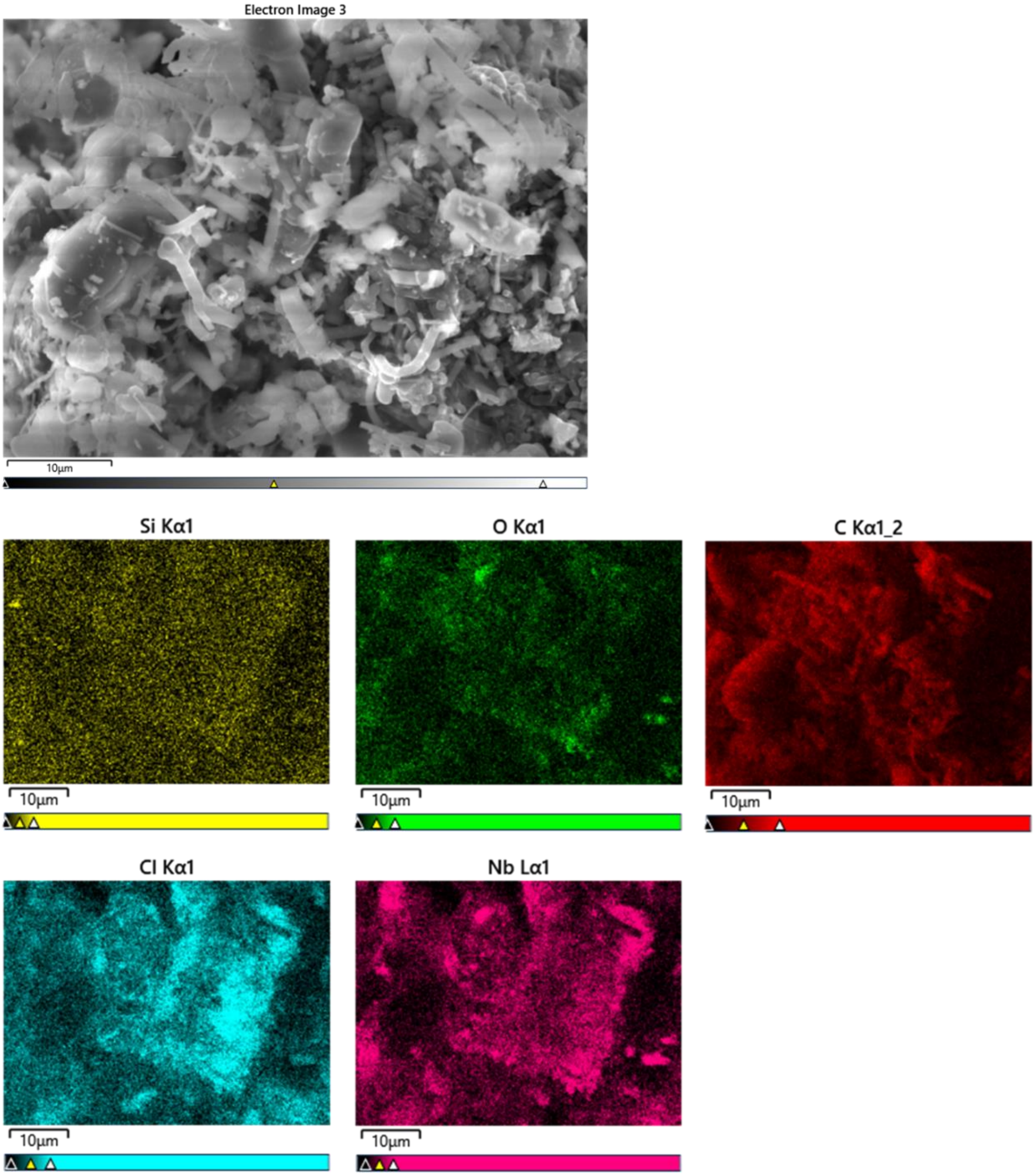

Figure 23: Scanning electron microscopy images with elemental mapping of the spent $NbCl_5$ catalyst after the gas-phase cyclotrimerization of acetylene to benzene (C_2H_2/N_2 1:10, 150 °C, 3 bar, WHSV ca. 6 600 $cm^3\ h^{-1}\ g_{cat}^{-1}$) showing the plate- and fiber-like carbon growths on the surface of the catalyst.

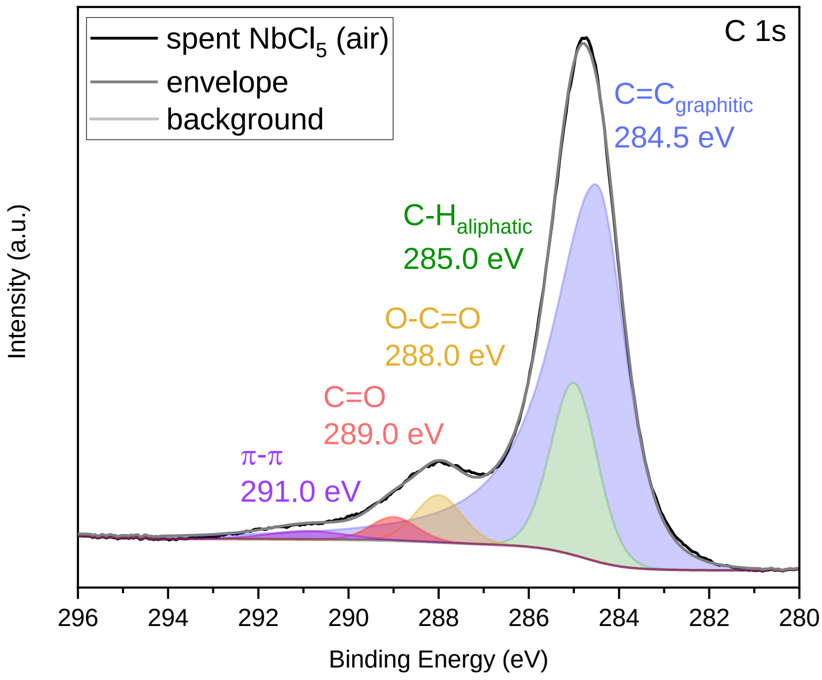

Figure 24: X-ray photoelectron spectrum of the C 1s region (referenced to $C=C_{graphitic}$ = 284.5 eV) of the spent $NbCl_5$ catalyst (in air contact) after gas-phase cyclotrimerization of acetylene to benzene (pure C_2H_2, 180 °C, 1.5 bar, WHSV 4 800 $cm^3\ h^{-1}\ g_{cat}^{-1}$).

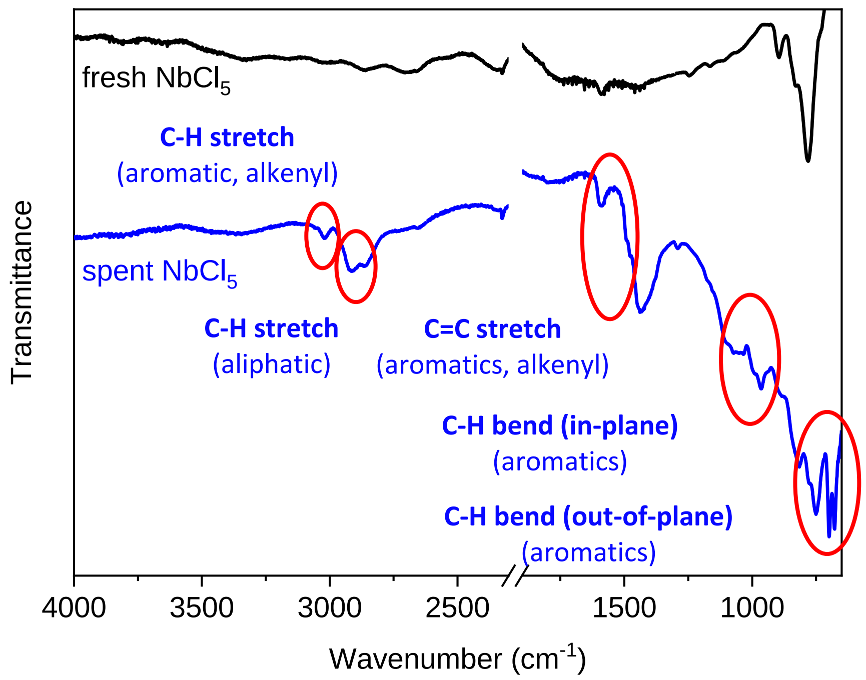

Figure S25: Fourier-transform infrared spectroscopy (FT-IR) of the pristine and spent $NbCl_5$ catalyst revealing aromatic and aliphatic compounds in the carbonaceous deposit of the spent $NbCl_5$ catalyst with bands at 3022 cm^{-1} (aromatic/sp² C-H stretch), 2917 cm^{-1} (aliphatic C-H stretch), multiple bands between 1594 and 1439 cm^{-1} (aliphatic C-H bend or aromatic C=C ring mode), bands at 1035 cm^{-1} (aromatic C-H in-plane bend) and 678 cm^{-1} (aromatic C-H out-of-plane bend).

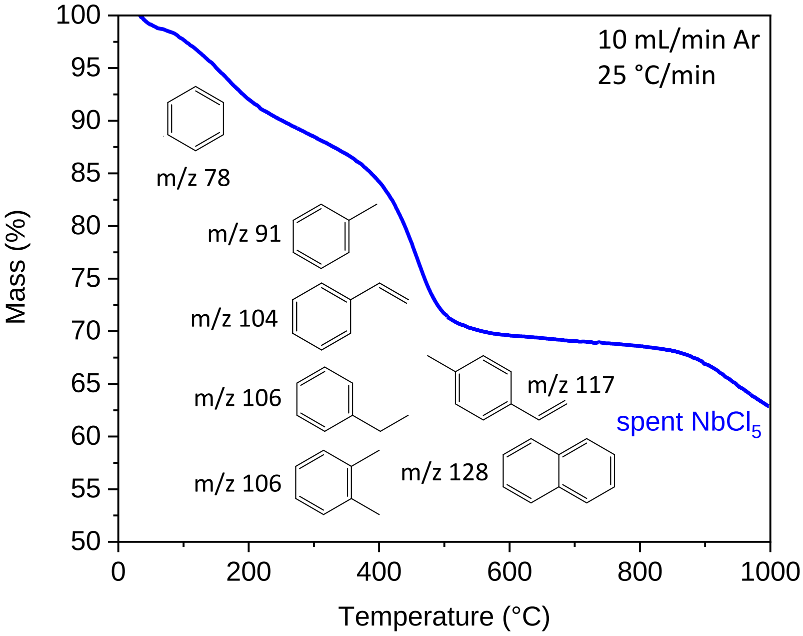

Figure S26: Thermogravimetric analysis coupled with a mass spectrometer of the spent $NbCl_5$ catalyst after the gas-phase cyclotrimerization of acetylene to benzene (C_2H_2/N_2 1:10, 150 °C, 3 bar, WHSV ca. 6 600 $cm^3\ h^{-1}\ g_{cat}^{-1}$) showing the desorption of (or the decomposition of the carbonaceous carbon deposit to) different aromatics with increasing temperature.

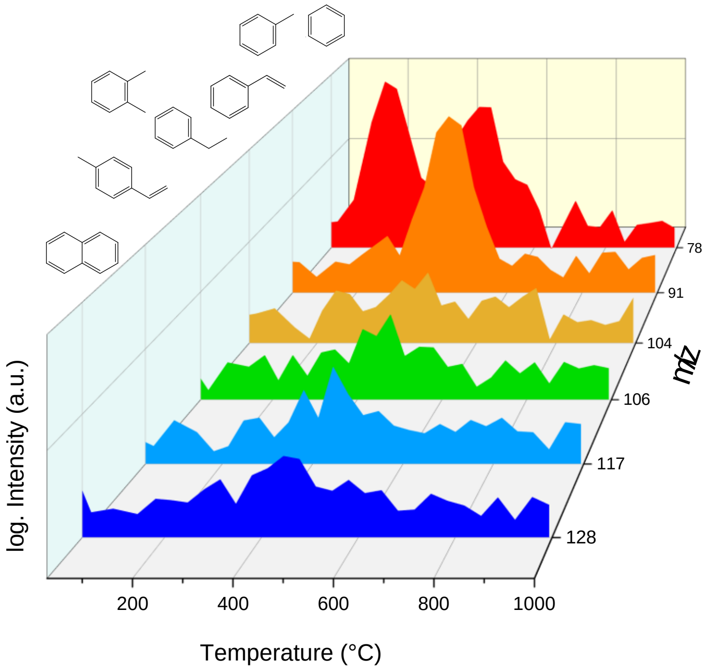

Figure S27: Selected mass spectrometer signals during the coupled thermogravimetric analysis of the spent $NbCl_5$ catalyst after the gas-phase cyclotrimerization of acetylene to benzene (C_2H_2/N_2 1:10, 150 °C, 3 bar, WHSV ca. 6 600 $cm^3\ h^{-1}\ g_{cat}^{-1}$) showing the desorption of (or the decomposition of the carbonaceous carbon deposit to) different aromatics at different temperatures.

(a)

(b)

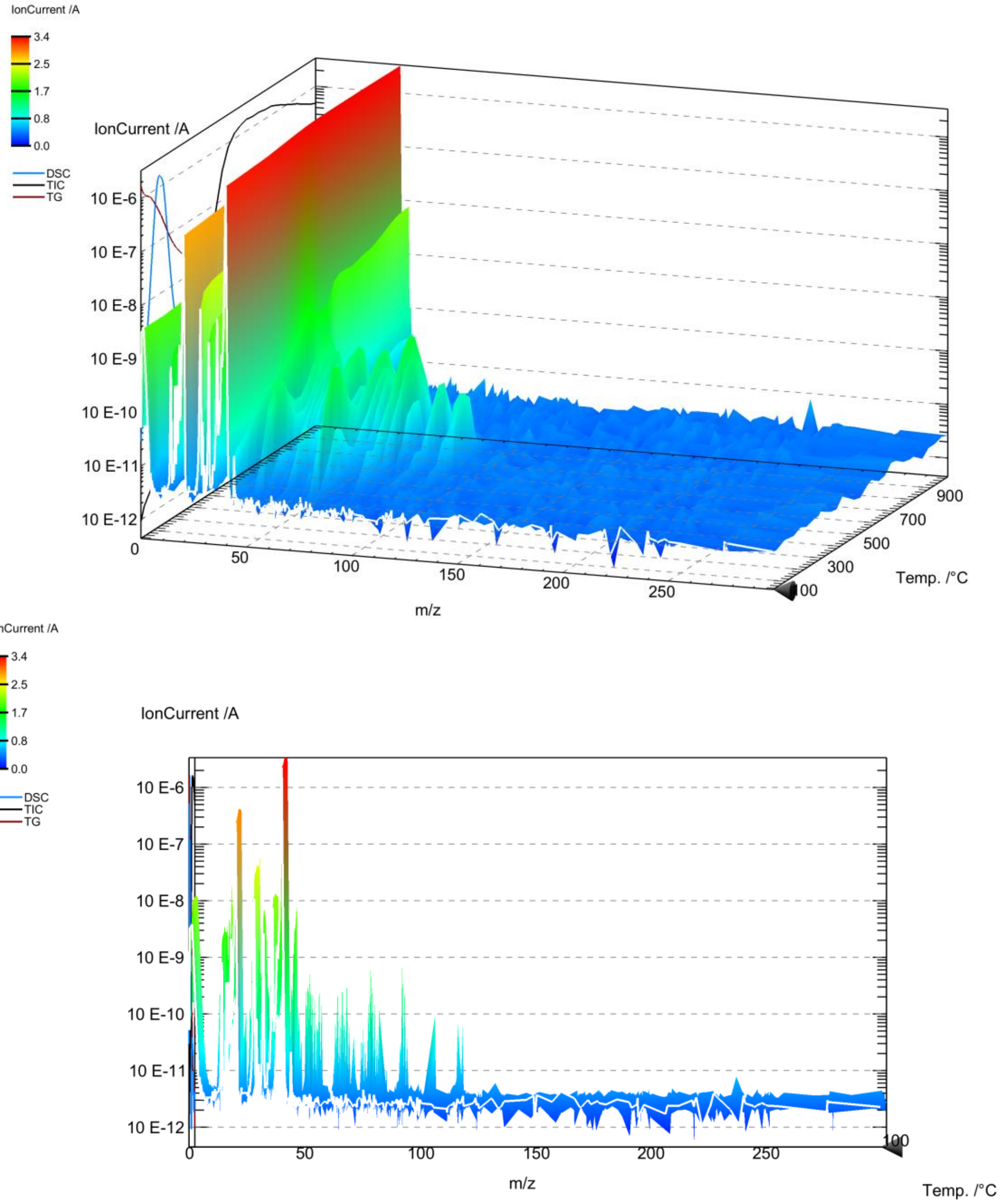

Figure S28: (a) and (b) overview of the mass spectrometer signals during the coupled thermogravimetric analysis of the spent $NbCl_5$ catalyst after the gas-phase cyclotrimerization of acetylene to benzene (C_2H_2/N_2 1:10, 150 °C, 3 bar, WHSV ca. 6 600 $cm^3\ h^{-1}\ g_{cat}^{-1}$).

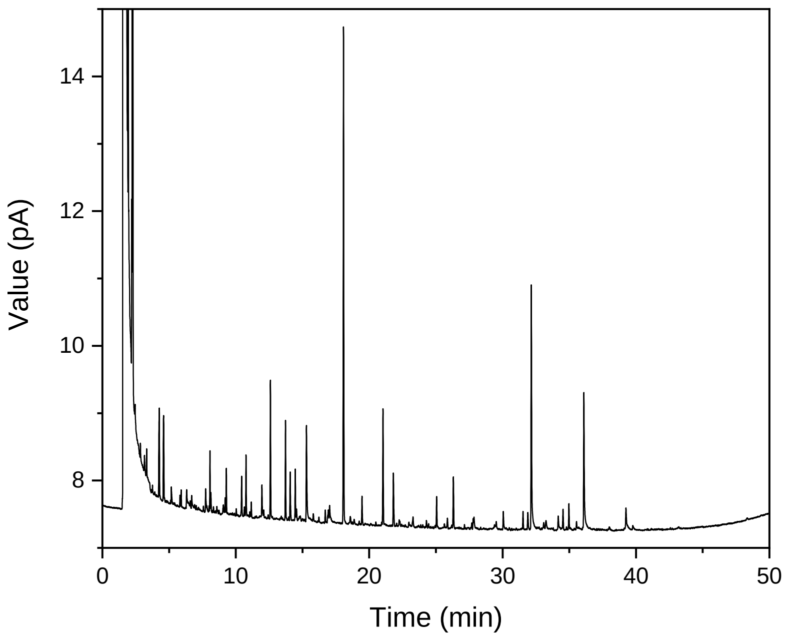

Figure S29: GC measurement coupled with MS of the dissolved species from quenching the spent $NbCl_5$ catalyst after the gas-phase cyclotrimerization of acetylene to benzene (C_2H_2/N_2 1:10, 150 °C, 3 bar, WHSV ca. 6 600 $cm^3\ h^{-1}\ g_{cat}^{-1}$) in MeOH.

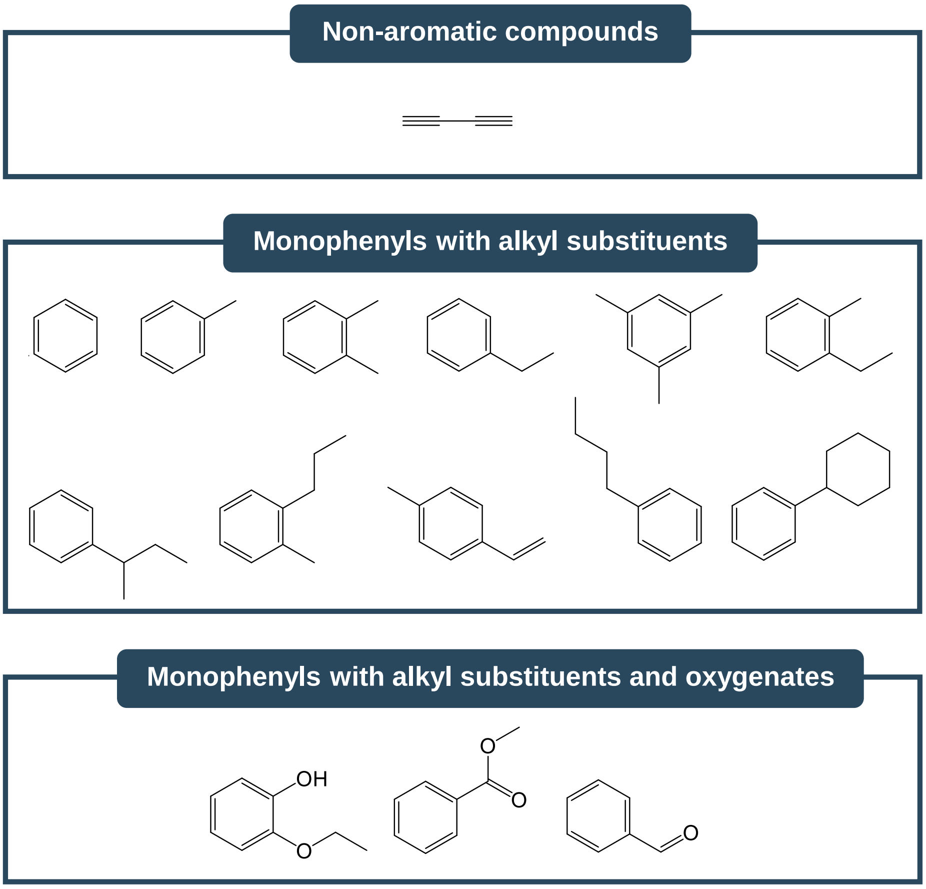

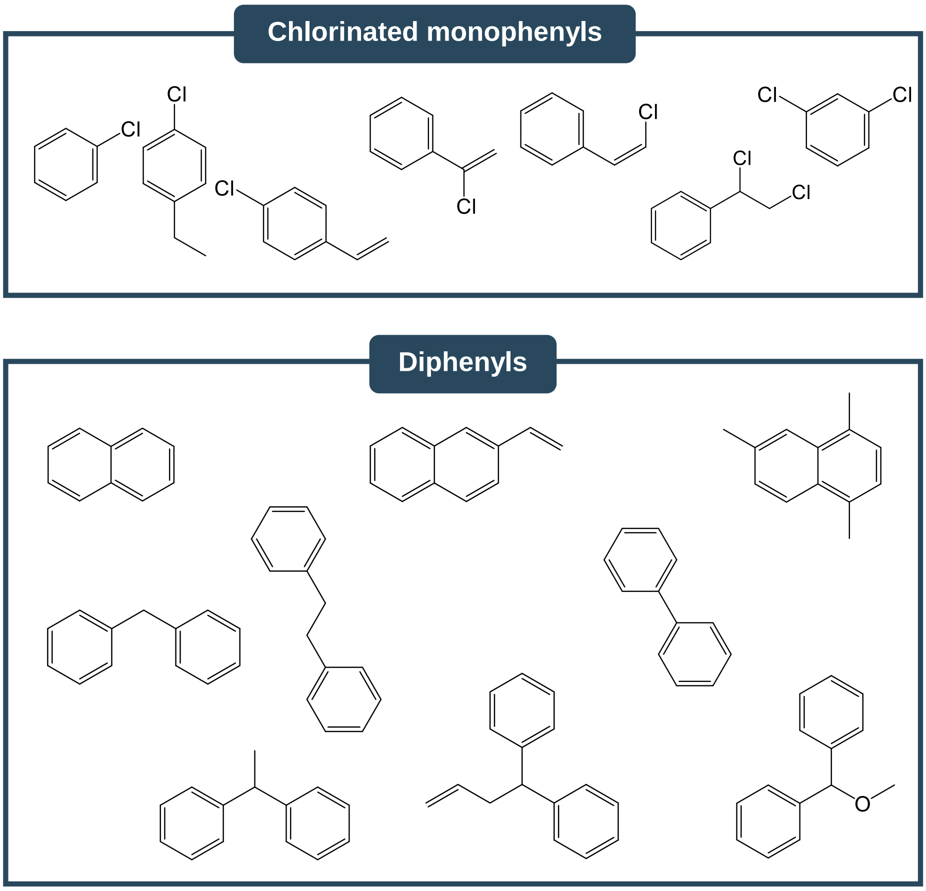

Figure 30: Dissolved species during quenching of the spent $NbCl_5$ catalyst after the gas-phase cyclotrimerization of acetylene to benzene (C_2H_2/N_2 1:10, 150 °C, 3 bar, WHSV ca. 6 600 $cm^3\ h^{-1}\ g_{cat}^{-1}$) in MeOH identified via GC-MS.

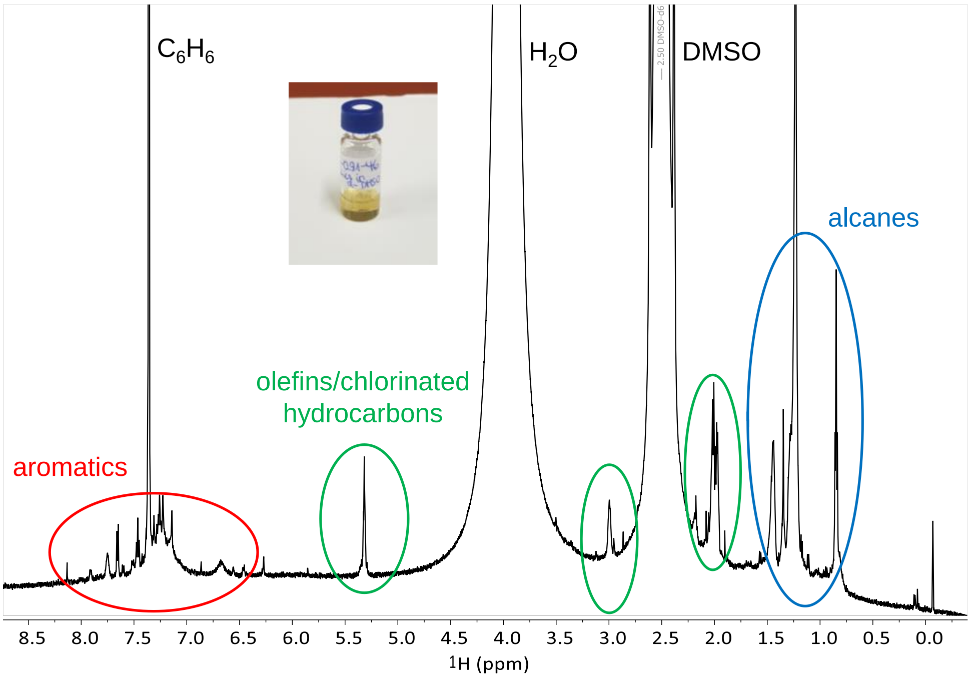

Figure 31: ^{1}H-NMR spectrum of a filtered slurry of the spent $NbCl_5$ catalyst after the gas-phase cyclotrimerization of acetylene to benzene (C_2H_2/N_2 1:10, 150 °C, 3 bar, WHSV ca. 6 600 cm³ h^{-1} g_{cat}^{-1}) in d_6-DMSO demonstrating the presence of various aromatics, olefinic and saturated alkyl species in the carbonaceous deposit after reaction.

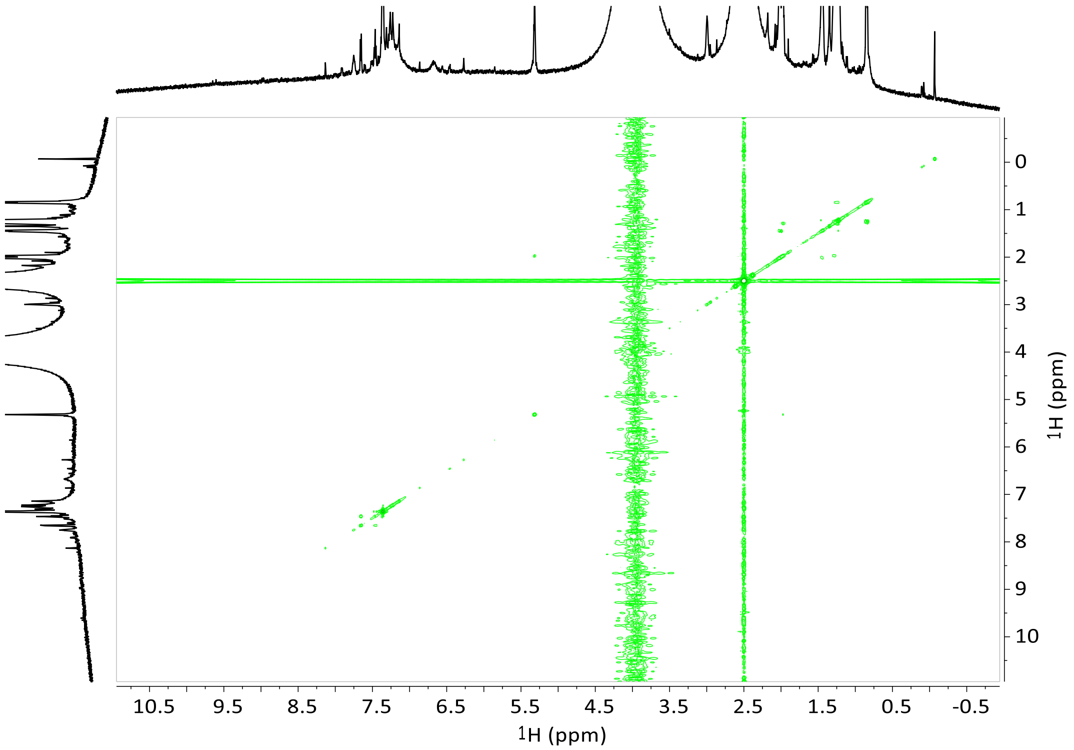

Figure 32: ^{1}H-^{1}H-COSY NMR spectrum of a filtered slurry of the spent $NbCl_5$ catalyst after the gas-phase cyclotrimerization of acetylene to benzene (C_2H_2/N_2 1:10, 150 °C, 3 bar, WHSV ca. 6 600 cm³ h^{-1} g_{cat}^{-1}) in d_6-DMSO demonstrating the presence of various aromatics, olefinic and saturated alkyl species in the carbonaceous deposit after reaction.

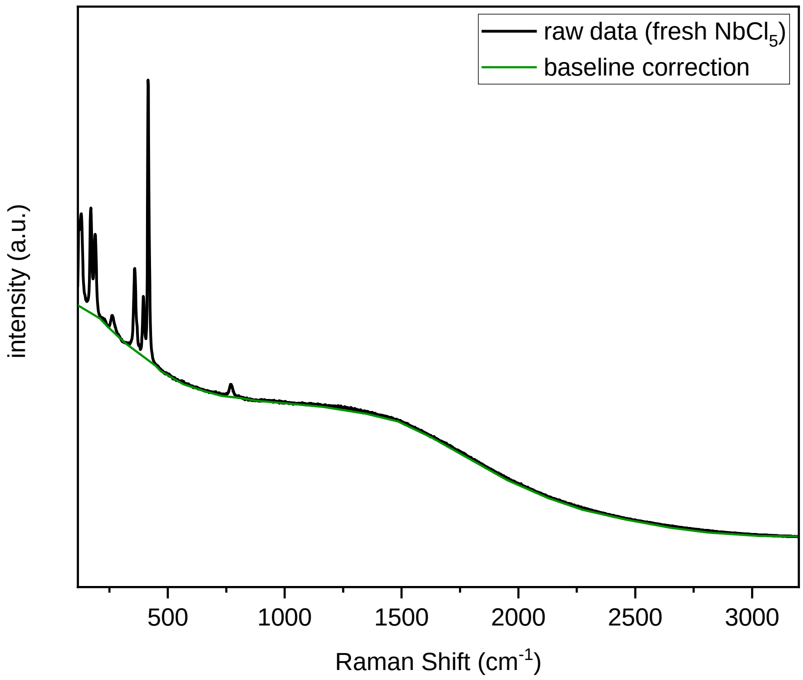

Figure 33: Raman spectrum of the fresh $NbCl_5$ with characteristic bands (data from Figure 1c).

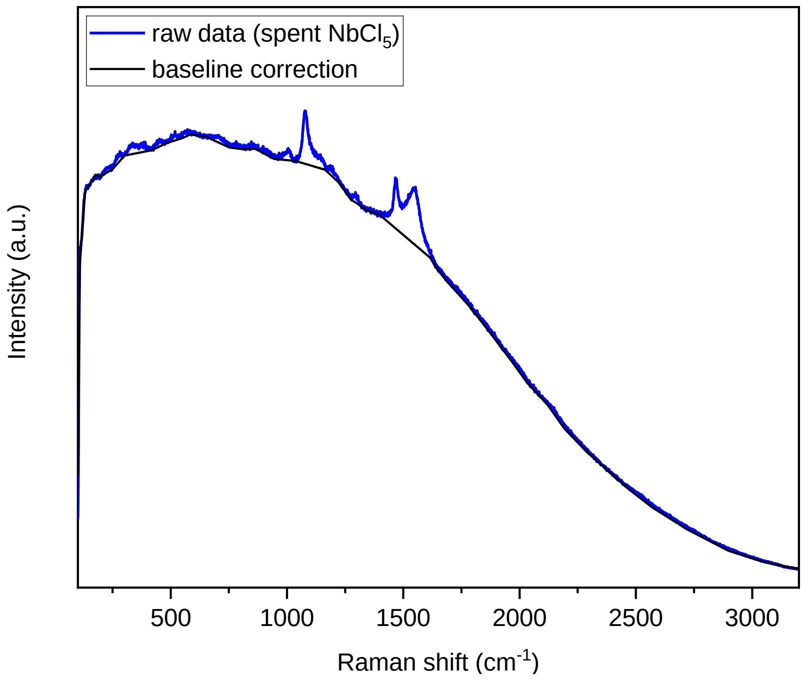

Figure 34: Raman spectrum of the spent $NbCl_5$ catalyst after the gas-phase cyclotrimerization of acetylene (C_2H_2/H_2 1:10, 150 °C, 3 bar, WHSV 6 600 $cm^3\ h^{-1}\ g_{cat}^{-1}$) revealing characteristic bands of *trans*-polyacetylene (data from Figure 1c).

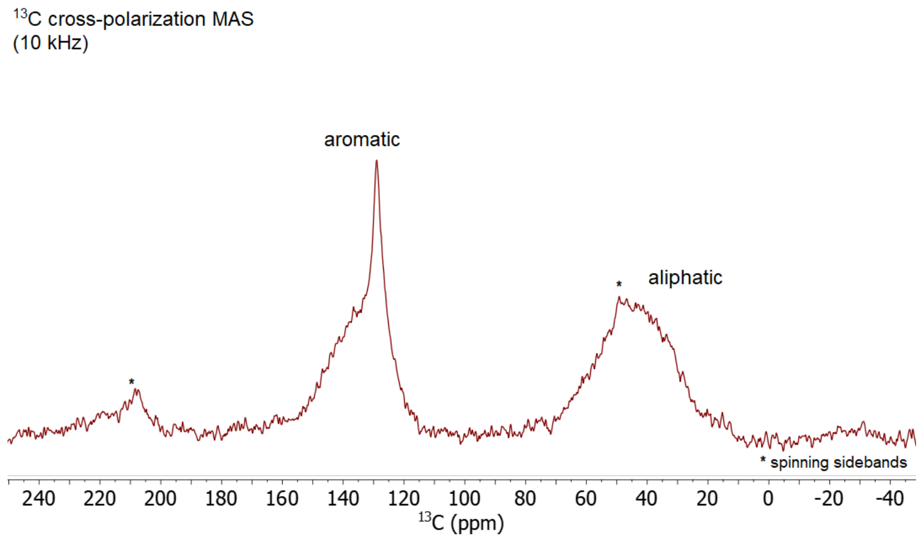

Figure S35: ^{13}C CP-MAS NMR spectra of the spent $NbCl_5$ catalyst after the gas-phase cyclotrimerization of acetylene to benzene (C_2H_2/N_2 1:10, 150 °C, 3 bar, WHSV ca. 6 600 cm^3 h^{-1} g_{cat}^{-1}) revealing the presence of both small aromatic molecules with a sharp signal at 129 ppm, as well as defect-rich *trans*-polyacetylene with a broad shoulder peak around 137 ppm. The signal for aliphatic species most likely result from substituents of the small aromatic molecules or the sp^3-hydridized terminal groups of *trans*-polyacetylene.

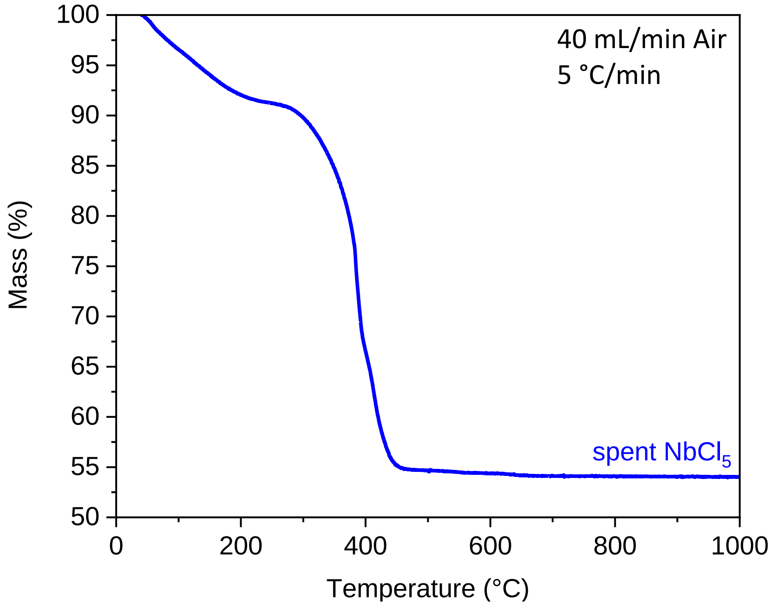

Figure S36: Thermogravimetric analysis of the spent $NbCl_5$ catalyst under air flow after the gas-phase cyclotrimerization of acetylene to benzene (C_2H_2/N_2 1:10, 150 °C, 3 bar, WHSV ca. 6 600 cm^3 h^{-1} g_{cat}^{-1}) revealing the evaporation of volatile species until 250 °C (-9 wt.%) followed by the oxidative decomposition of encapsulating *trans*-polyacetylene between 300 and 450 °C (-36 wt.%) as reported by Luo and coworkers.[11]

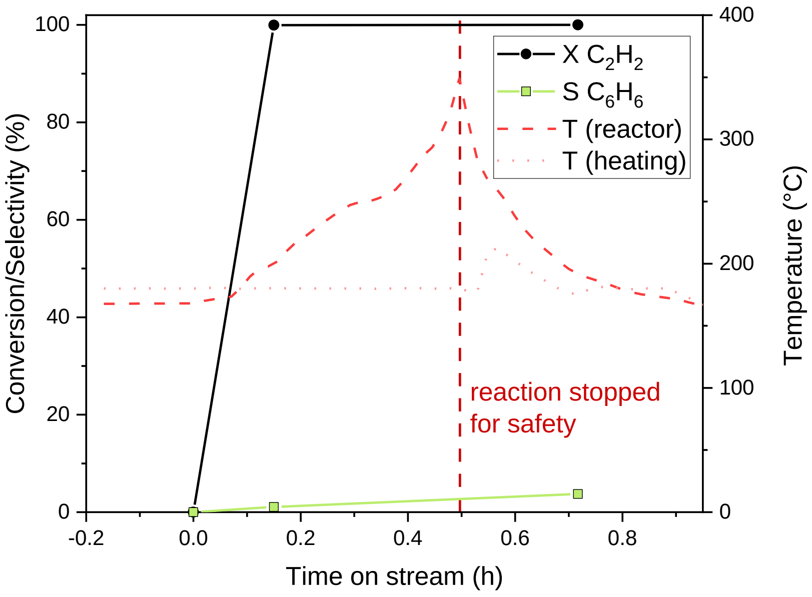

Figure S37: Selectivity to benzene, acetylene conversion and temperature profiles for $NbCl_5$ in the gas-phase cyclotrimerization of acetylene to benzene (pure C_2H_2, 180 °C, 1.5 bar, WHSV 4 800 cm^3 h^{-1} g_{cat}^{-1}). The high exothermicity of the reaction and formation of carbonaceous deposits lead to a catalyst bed temperature of up to 350 °C and a fast reactor blocking.

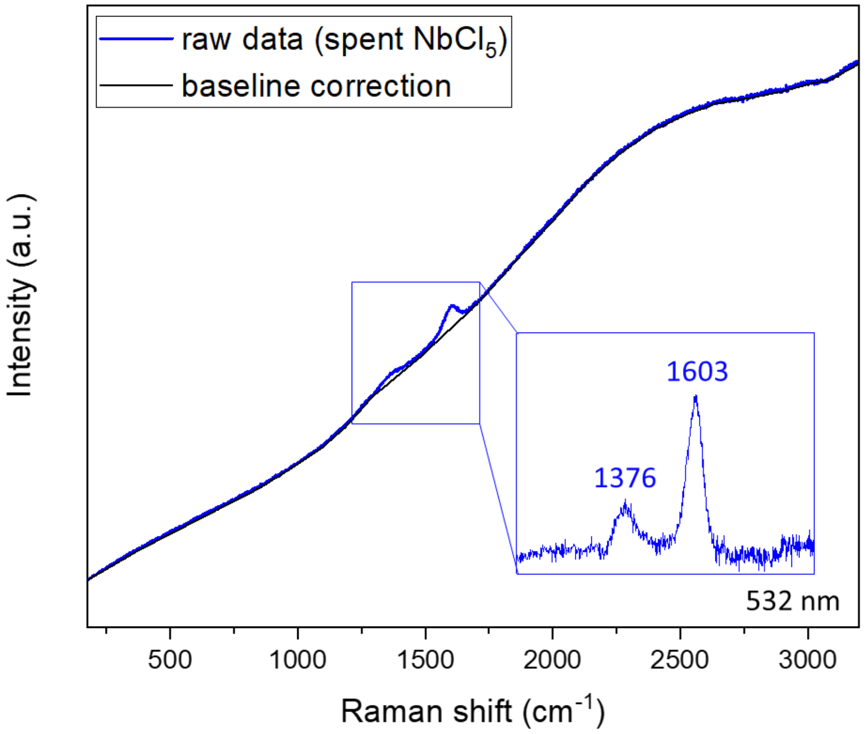

Figure 38: Raman spectrum of the spent $NbCl_5$ catalyst after the gas-phase cyclotrimerization with undiluted acetylene (180 °C, 1.5 bar, WHSV 4800 cm^3 h^{-1} g_{cat}^{-1}, Figure S37) revealing the typical bands for graphitic carbons.

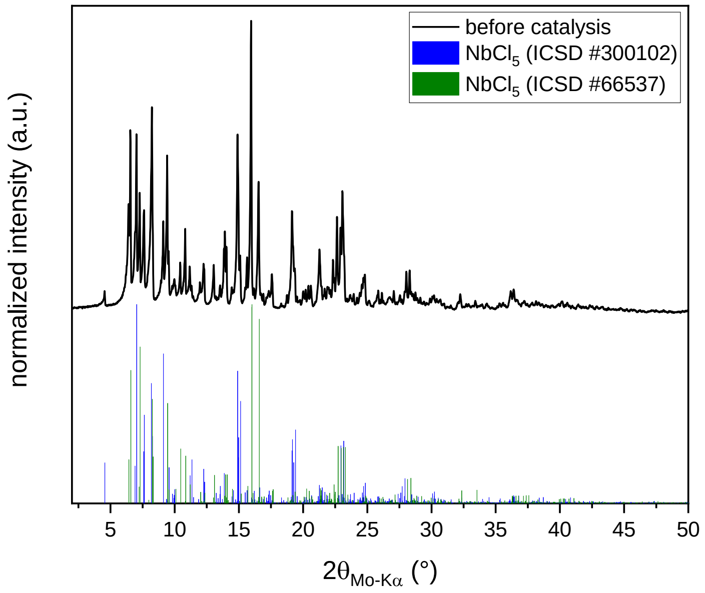

Figure S39: Powder X-ray diffraction (PXRD) pattern of pristine $NbCl_5$ revealing the presence of two $NbCl_5$ polymorphs upon comparison with references from ICSD crystal data base.

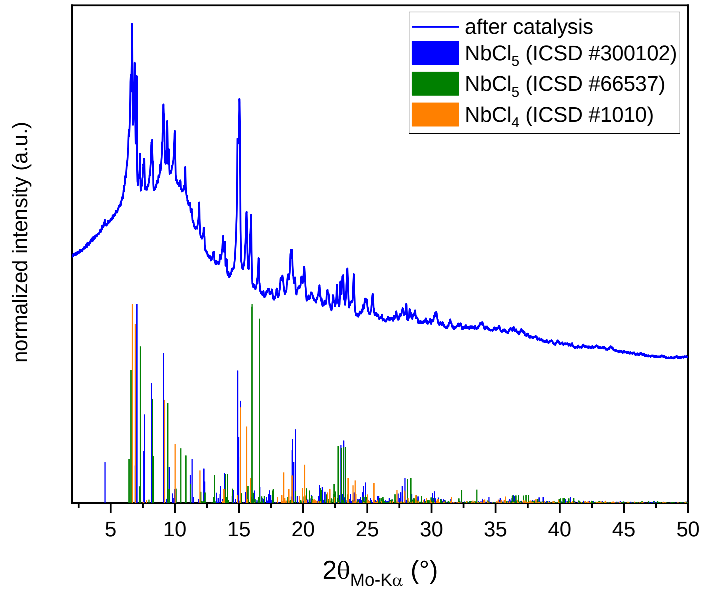

Figure S40: Powder X-ray diffraction (PXRD) pattern of spent $NbCl_5$ catalyst after the gas-phase cyclotrimerization of acetylene to benzene (C_2H_2/N_2 1:10, 150 °C, 3 bar, WHSV ca. 6 600 $cm^3\ h^{-1}\ g_{cat}^{-1}$) revealing the presence of additional reflections attributable to a $NbCl_4$ phase formed after reaction upon comparison with references from ICSD crystal data base.

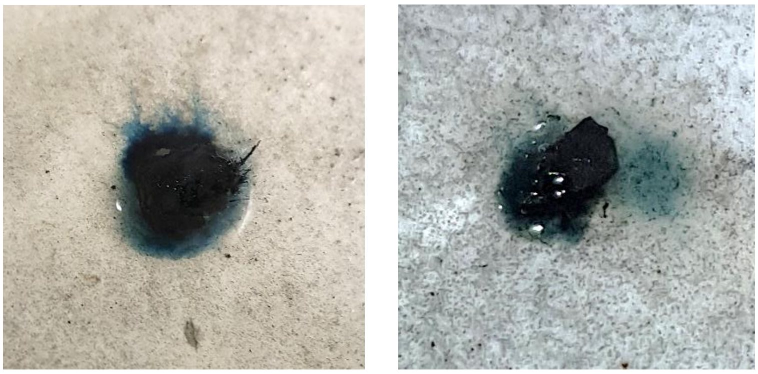

Figure S41: Characteristic blue colored aqua complexes of $NbCl_4$ in the spent $NbCl_5$ catalyst upon contact with humidity/water during cleaning of reactor.

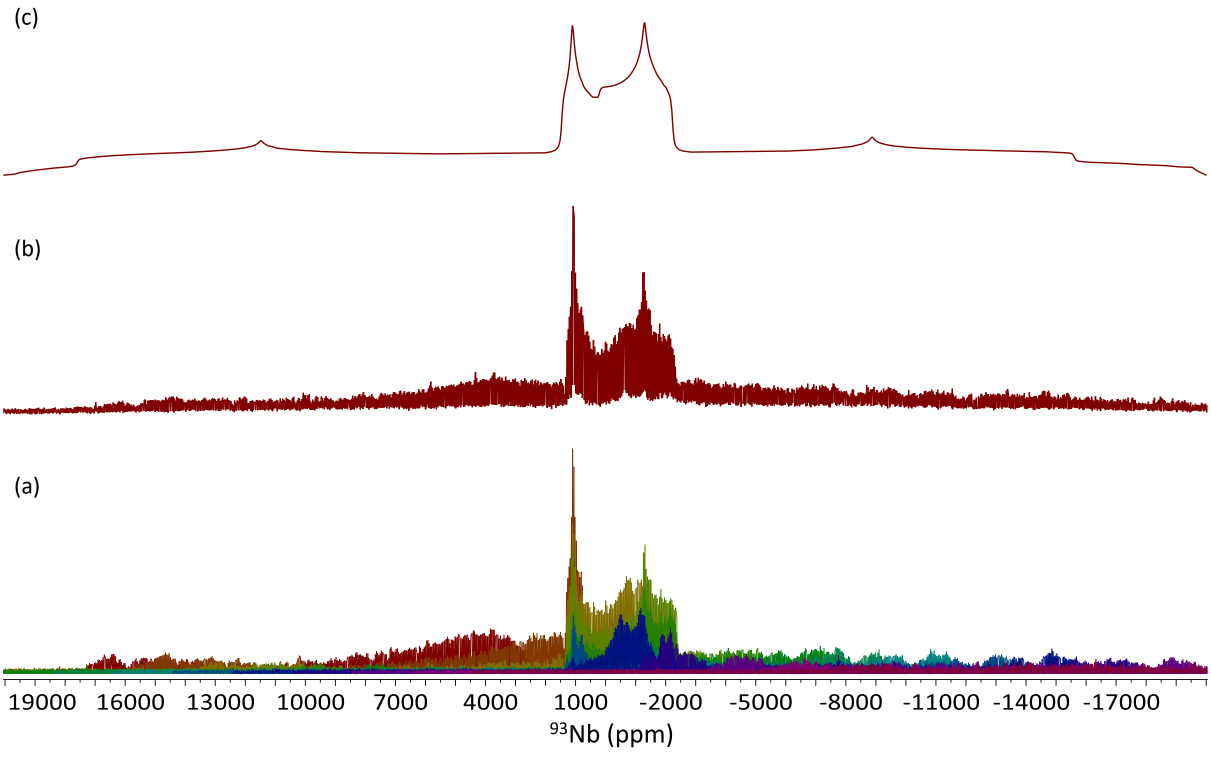

Figure S42: ^{93}Nb *solid-state* NMR spectrum of pristine $NbCl_5$ (a) Overlay of 14 spectral fragments (differently colored) via a WURST-QCPMG sequence with carrier frequency incremented by 2000 ppm from +20 000 to -20 000 ppm. (b) Full spectrum obtained by co-addition of the 14 spectral fragments from (a). (c) Simulated static powder spectrum of ^{93}Nb with C_q = 80 MHz, η = 0.25 and δ_{iso} = +180 ppm (sola/topspin 3.6).

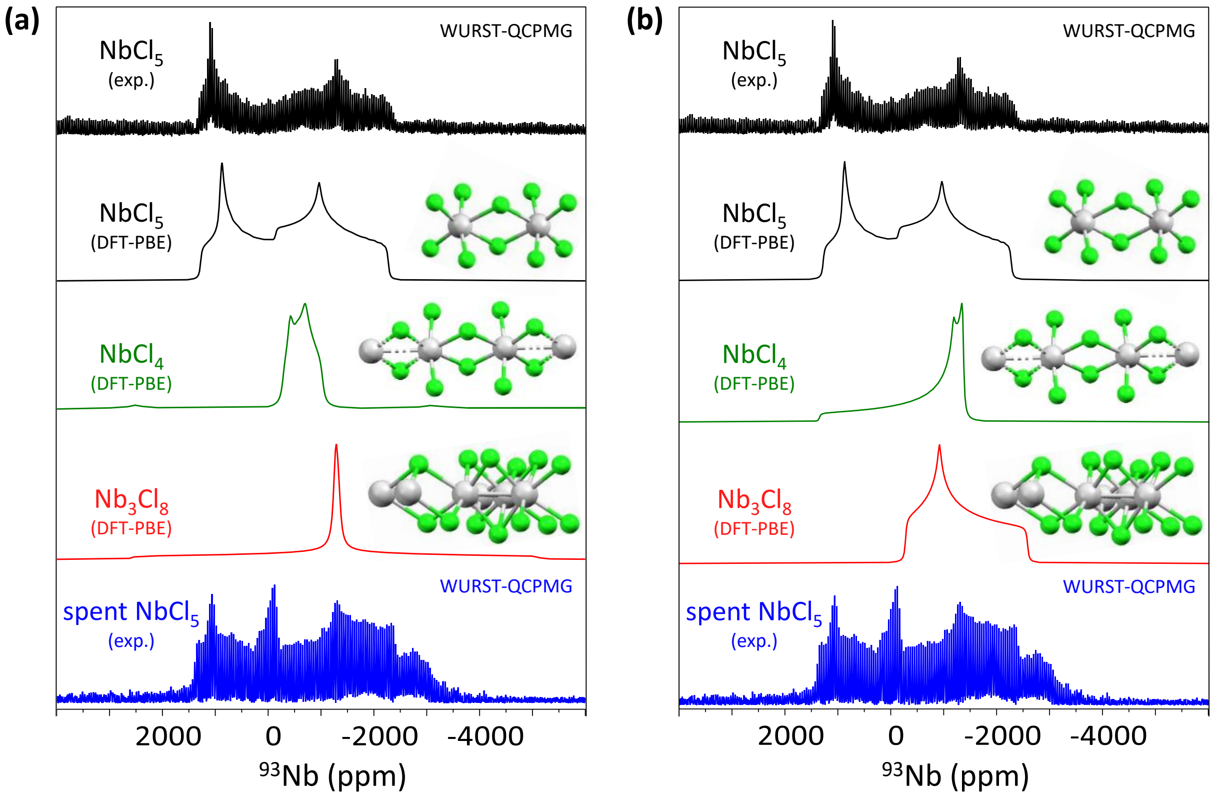

Figure S43: ^{93}Nb *solid-state* NMR spectra of the central +1/2 ↔ -1/2 transition (m_z) of fresh $NbCl_5$ (black) and spent $NbCl_5$ (blue) extracted from a co-addition of 14 single spectral fragments via a WURST-QCPMG pulse sequence. DFT-PBE calculated ^{93}Nb *solid-state* NMR spectra of the central transition for $NbCl_5$ (black), $NbCl_4$ (green) and Nb_3Cl_8 (red) with corresponding coordination sphere of niobium for comparison without (a) and with (b) taking the calculated chemical shift anistropy (CSA) into account.

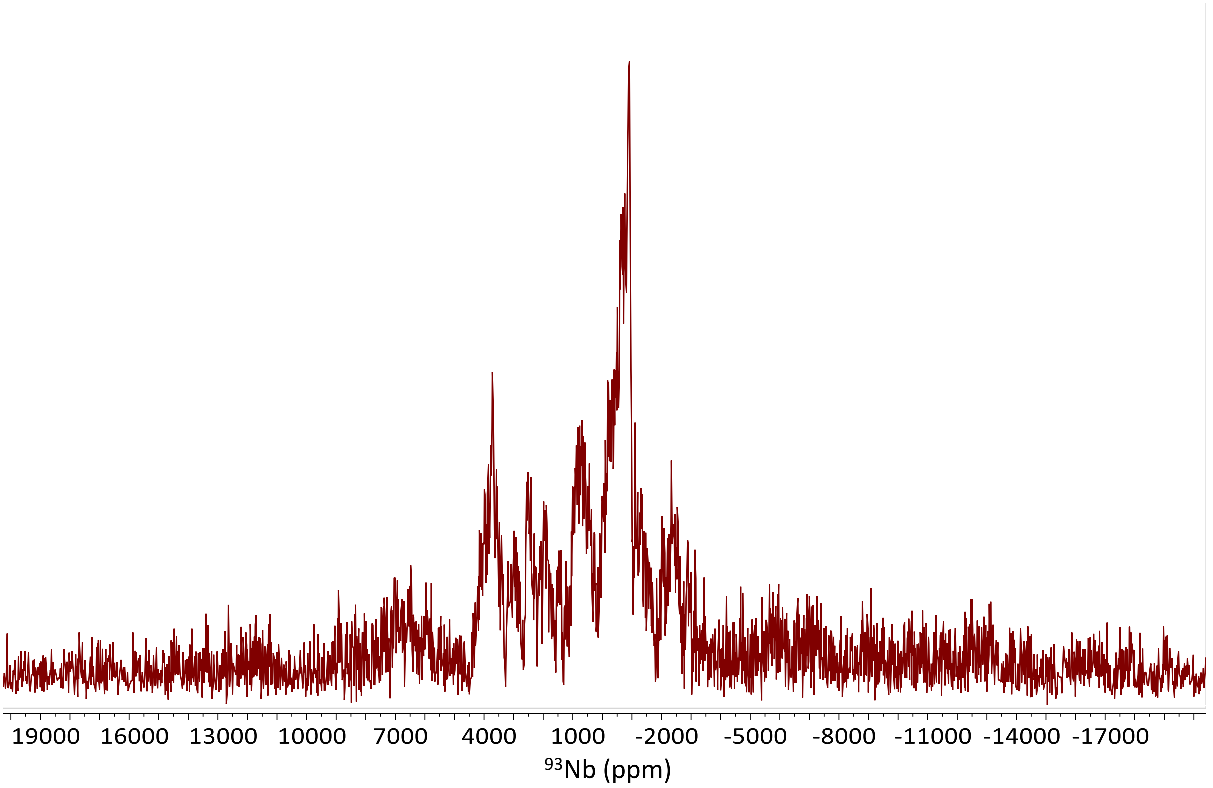

Figure S44: ^{93}Nb *solid-state* NMR spectrum of $NbCl_4(THF)_2$ reference. Since relaxation effects impeded the measurement of echos via the WURST-QCPMG method, ^{93}Nb was measured as a simple one-pulse excitation at a MAS rotation frequency of 12 000 Hz. The central line has an approximate width of 95 MHz corresponding roughly to a C_q of 30-40 MHz. The spectrum is presented in magnitude mode.

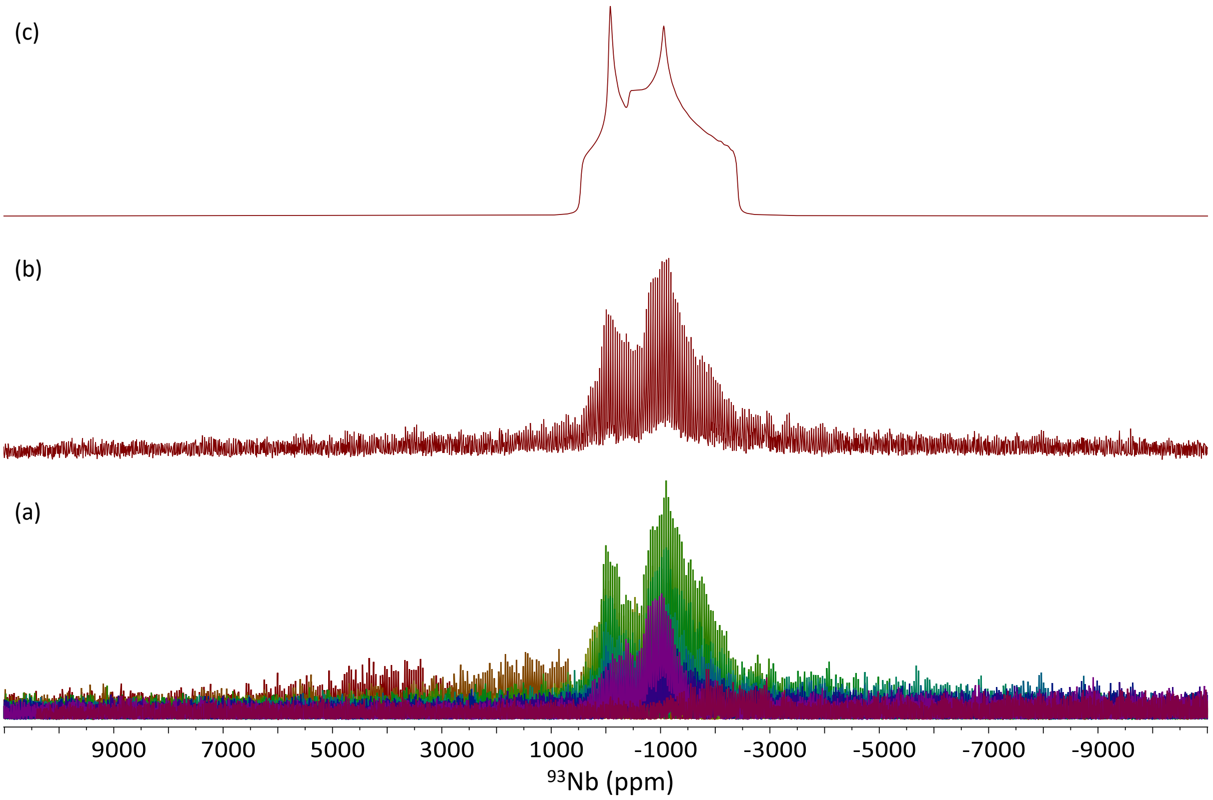

Figure S45: ^{93}Nb *solid-state* NMR spectrum of $NbCl_3$(DME). (a) Overlay of 11 spectral fragments (differently colored) via a WURST-QCPMG sequence with carrier frequency incremented by 2000 ppm from +10 000 to -10 000 ppm. (b) Full spectrum obtained by co-addition of the 11 spectral fragments from (a). (c) Simulated static powder spectrum of ^{93}Nb with C_q = 64 MHz, η = 0.55 and δ_{iso} = -500 ppm (sola/topspin 3.6).

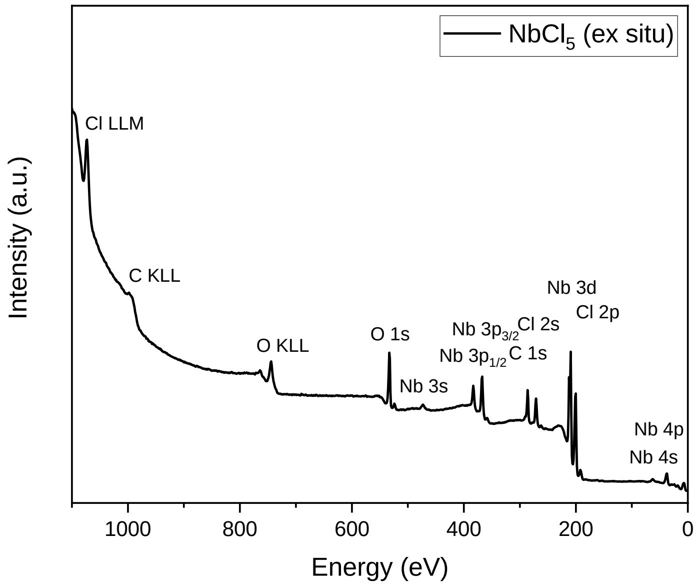

Figure S46: XPS survey spectrum of inert handled pristine $NbCl_5$ (referenced to Nb $3d_{5/2}$ = 207.9 eV for $NbCl_5$).

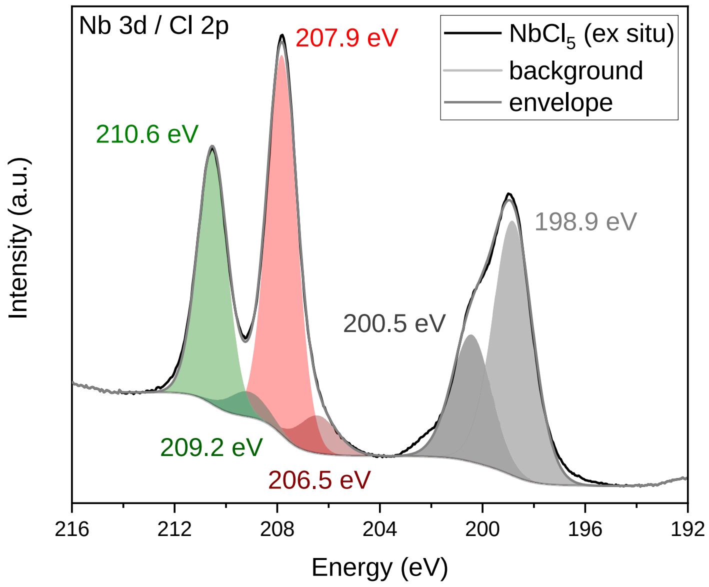

Figure S47: XPS spectrum of Nb 3d ($3d_{3/2}$ and $3d_{5/2}$) and Cl 2p ($2p_{1/2}$ and $2p_{3/2}$) region (referenced to Nb $3d_{5/2}$ = 207.9 eV for $NbCl_5$) of inert handled pristine $NbCl_5$ exhibiting two different Nb species. Specie at higher binding energies most likely belongs to Nb^{5+} bound in $NbCl_5$ while the one at lower energies might originate from Nb^{5+} bound to oxygen from surface impurities and one chlorine specie.

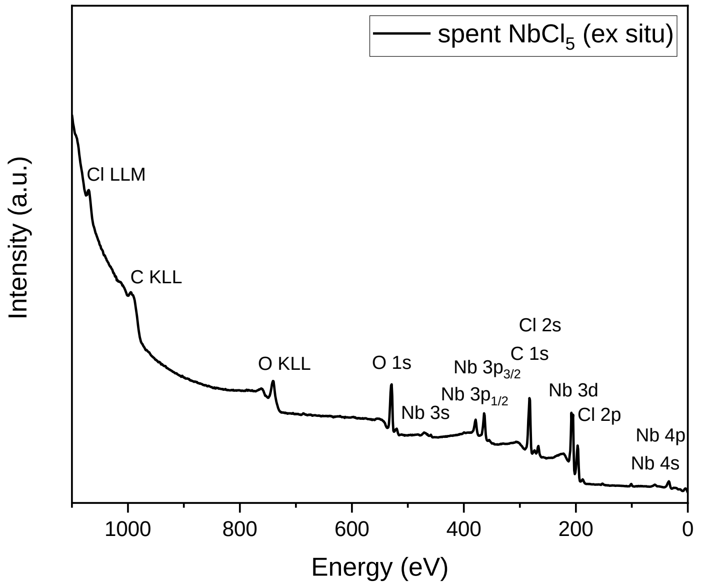

Figure S48: XPS survey spectrum of inert handled spent $NbCl_5$ (referenced to Nb $3d_{5/2}$ = 207.9 eV for $NbCl_5$) after gas-phase cyclotrimerization of acetylene to benzene (C_2H_2/N_2 1:10, 120 °C, 3 bar, WHSV 6 600 $cm^3\ h^{-1}\ g_{cat}{}^{-1}$).

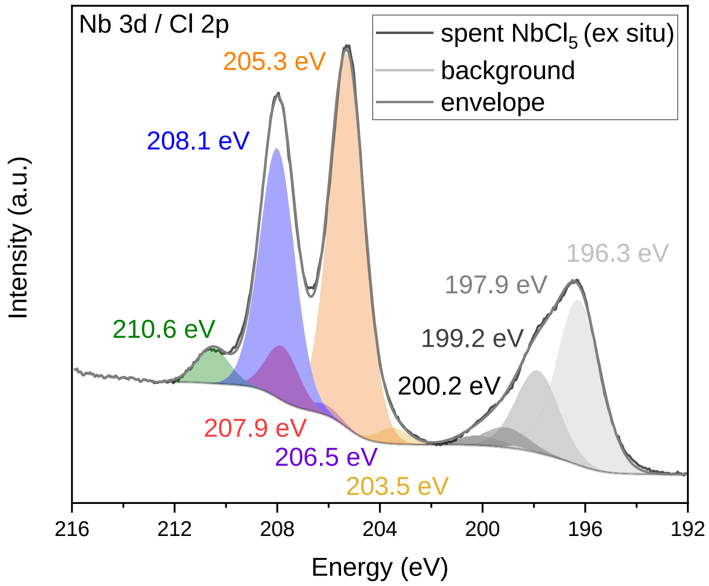

Figure S49: XPS spectrum of Nb 3d ($3d_{3/2}$ and $3d_{5/2}$) and Cl 2p ($2p_{1/2}$ and $2p_{3/2}$) region (referenced to Nb $3d_{5/2}$ = 207.9 eV for $NbCl_5$) of inert handled spent $NbCl_5$ after gas-phase cyclotrimerization of acetylene to benzene (C_2H_2/N_2 1:10, 120 °C, 3 bar, WHSV 6 600 $cm^3\ h^{-1}\ g_{cat}{}^{-1}$) exhibiting three different Nb species most likely belonging to Nb^{5+}, Nb^{4+} and Nb^{3+} with descending binding energy and two chlorine species.

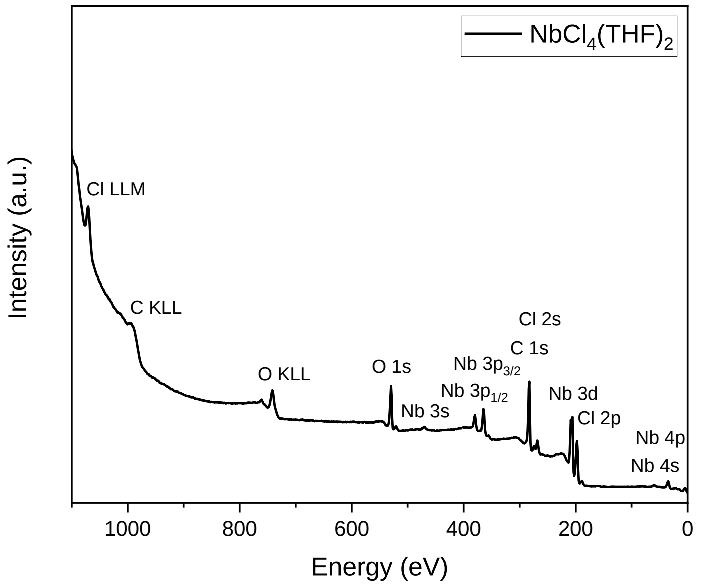

Figure S50: XPS survey spectrum of inert handled pristine $NbCl_4(THF)_2$ (referenced to Nb $3d_{5/2}$ = 207.9 eV for $NbCl_5$).

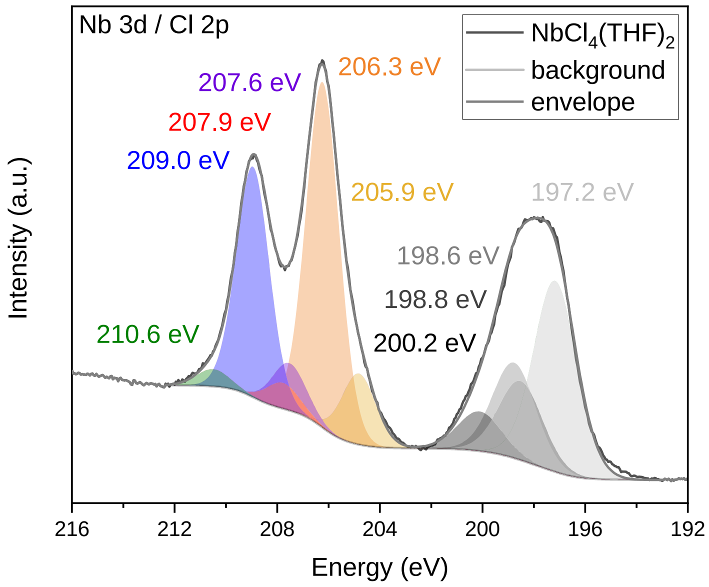

Figure S51: XPS spectrum of Nb 3d ($3d_{3/2}$ and $3d_{5/2}$) and Cl 2p ($2p_{1/2}$ and $2p_{3/2}$) region (referenced to Nb $3d_{5/2}$ = 207.9 eV for $NbCl_5$) of inert handled pristine $NbCl_4(THF)_2$ exhibiting three different Nb species most likely belonging to Nb^{5+}, Nb^{4+} and Nb^{3+} with descending binding energy and two different chlorine species.

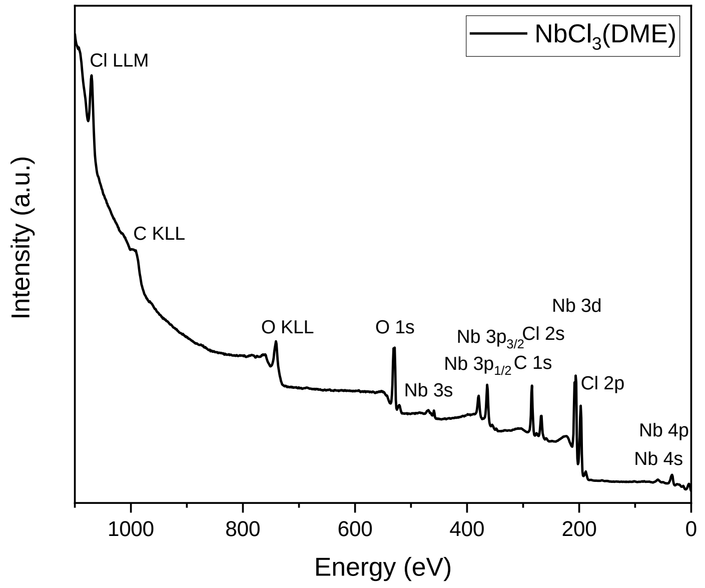

Figure S52: XPS survey spectrum of inert handled pristine NbCl$_3$(DME) (referenced to Nb 3d$_{5/2}$ = 207.9 eV for NbCl$_5$).

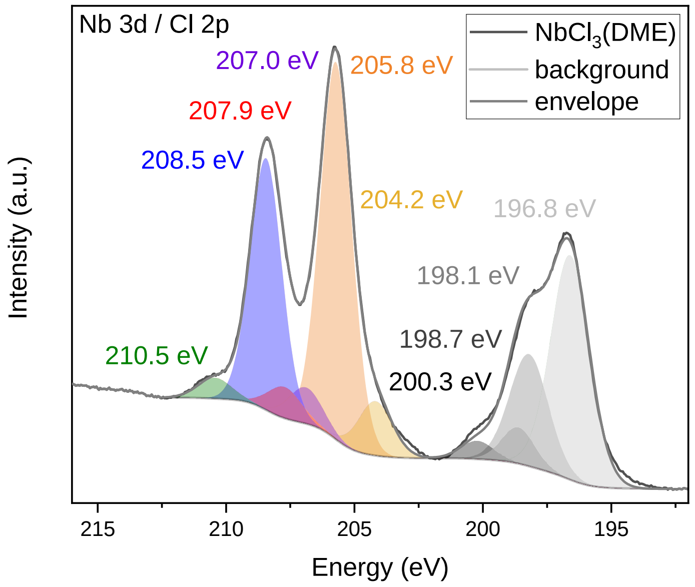

Figure S53: XPS spectrum of Nb 3d (3d$_{3/2}$ and 3d$_{5/2}$) and Cl 2p (2p$_{1/2}$ and 2p$_{3/2}$) region (referenced to Nb 3d$_{5/2}$ = 207.9 eV for NbCl$_5$) of inert handled pristine NbCl$_3$(DME) exhibiting three different Nb species most likely belonging to Nb^{5+}, Nb^{4+} and Nb^{3+} with descending binding energy and two different chlorine species.

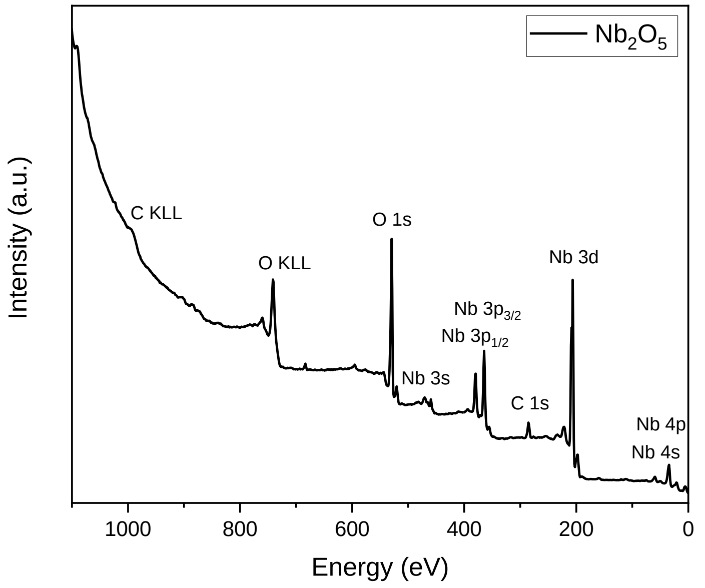

Figure S54: XPS survey spectrum of Nb_2O_5 (referenced to C 1s = 284.8 eV).

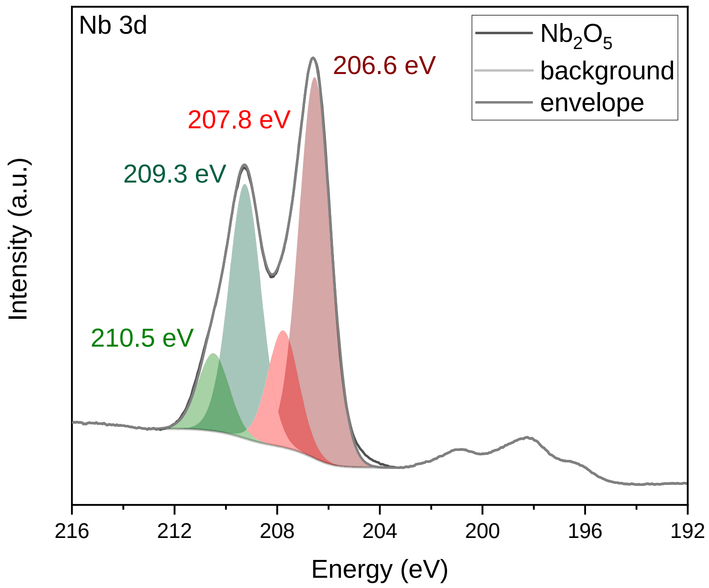

Figure S55: XPS spectrum of Nb 3d ($3d_{3/2}$ and $3d_{5/2}$) region (referenced to C 1s = 284.8 eV) of Nb_2O_5 exhibiting two different Nb species most likely belonging to Nb^{5+} bound in different proximities to O or some residual precursor material (e.g. $NbCl_5$).

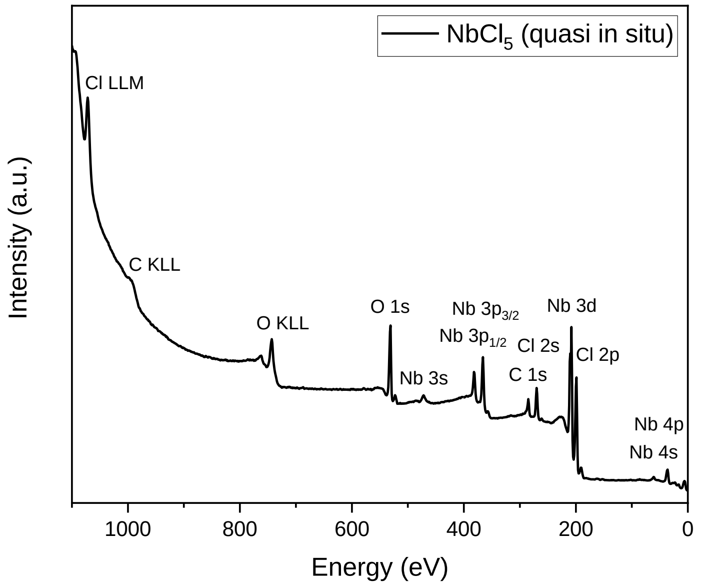

Figure S56: XPS survey spectrum of inert handled pristine $NbCl_5$ (referenced to C 1s = 284.8 eV) before the quasi in situ measurement.

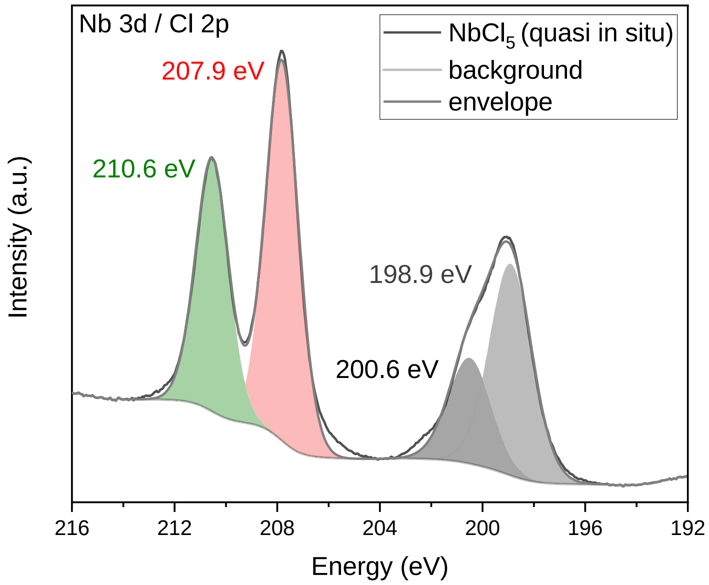

Figure S57: XPS spectrum of Nb 3d ($3d_{3/2}$ and $3d_{5/2}$) and Cl 2p ($2p_{1/2}$ and $2p_{3/2}$) region (referenced to C 1s = 284.8 eV) of inert handled pristine $NbCl_5$ before the quasi in situ measurement exhibiting one Nb specie most likely belonging to Nb^{5+} and one chlorine specie.

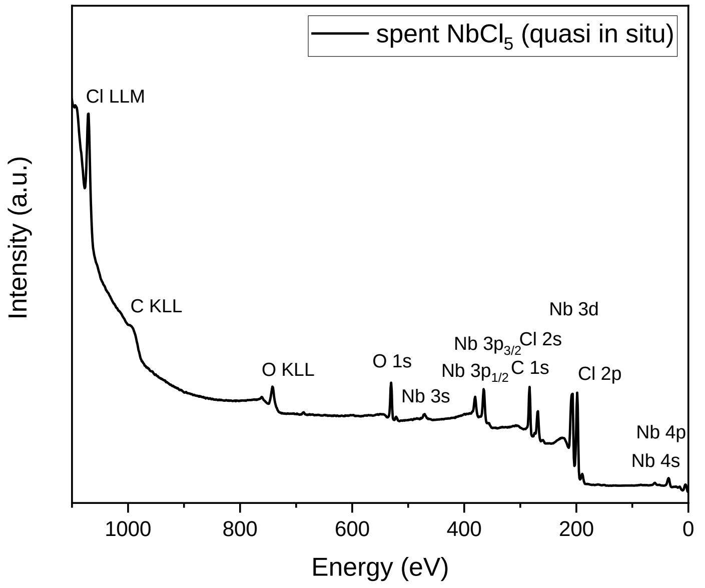

Figure S58: XPS survey spectrum of inert handled spent $NbCl_5$ (referenced to Nb $3d_{5/2}$ = 207.9 eV for $NbCl_5$) in a quasi in situ measurement (after ca. 1.5 h cool-down under N_2 flow and evacuation) after gas-phase cyclotrimerization of acetylene to benzene (C_2H_2/N_2 1:49, 120 °C, 1 atm, 1 h, WHSV ca. 30 000 cm^3 h^{-1} g_{cat} $^{-1}$).

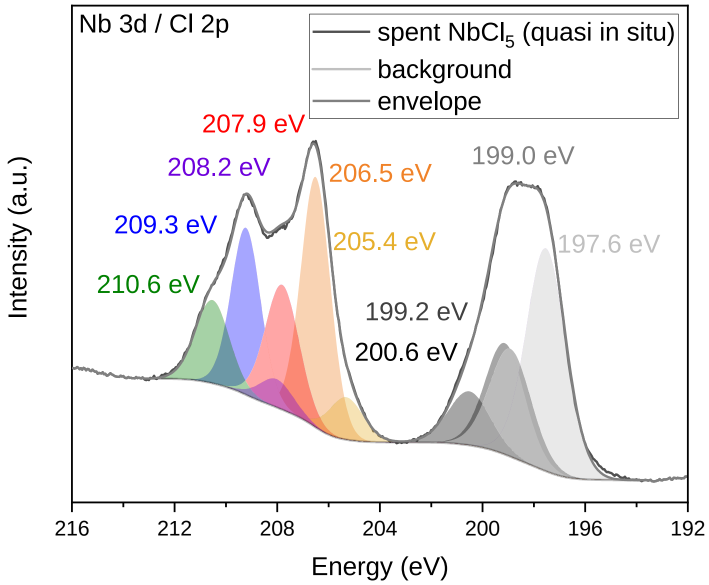

Figure S59: XPS spectrum of Nb 3d ($3d_{3/2}$ and $3d_{5/2}$) and Cl 2p ($2p_{1/2}$ and $2p_{3/2}$) region (referenced to Nb $3d_{5/2}$ = 207.9 eV for $NbCl_5$) of inert handled spent $NbCl_5$ in a quasi in situ measurement (after ca. 1.5 h cool-down under N_2 flow and evacuation) after gas-phase cyclotrimerization of acetylene to benzene (C_2H_2/N_2 1:49, 120 °C, 1 atm, 1 h, WHSV ca. 30 000 cm^3 h^{-1} g_{cat} $^{-1}$) exhibiting three different Nb species most likely belonging to Nb^{5+}, Nb^{4+} and Nb^{3+} with descending binding energy and two different chlorine species.

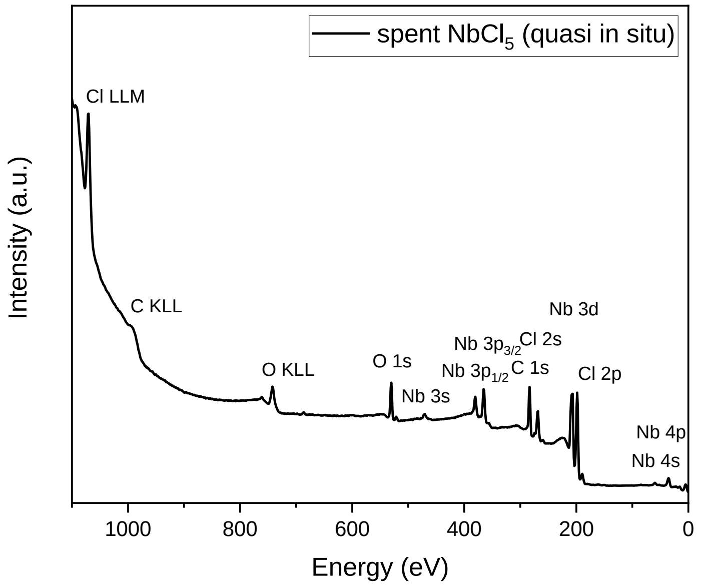

Figure S60: XPS survey spectrum of inert handled spent $NbCl_5$ (referenced to Nb $3d_{5/2}$ = 207.9 eV for $NbCl_5$) in a quasi in situ measurement (after ca. 1.5 h cool-down under N_2 flow and evacuation, stored in vacuum for 96 h) after gas-phase cyclotrimerization of acetylene to benzene (C_2H_2/N_2 1:49, 120 °C, 1 atm, 1 h, WHSV ca. 30 000 cm³ h⁻¹ g_{cat} ⁻¹).

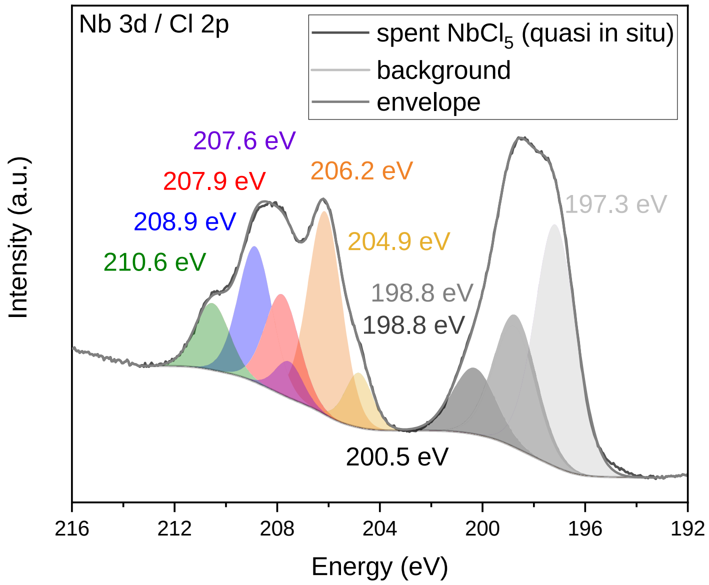

Figure S61: XPS spectrum of Nb 3d ($3d_{3/2}$ and $3d_{5/2}$) and Cl 2p ($2p_{1/2}$ and $2p_{3/2}$) region (referenced to Nb $3d_{5/2}$ = 207.9 eV for $NbCl_5$) of inert handled spent $NbCl_5$ in a quasi in situ measurement (after ca. 1.5 h cool-down under N_2 flow and evacuation, stored in vacuum for 96 h) after gas-phase cyclotrimerization of acetylene to benzene (C_2H_2/N_2 1:49, 120 °C, 1 atm, 1 h, WHSV ca. 30 000 cm³ h⁻¹ g_{cat} ⁻¹) exhibiting three different Nb species most likely belonging to Nb^{5+}, Nb^{4+} and Nb^{3+} with descending binding energy and two different chlorine species.

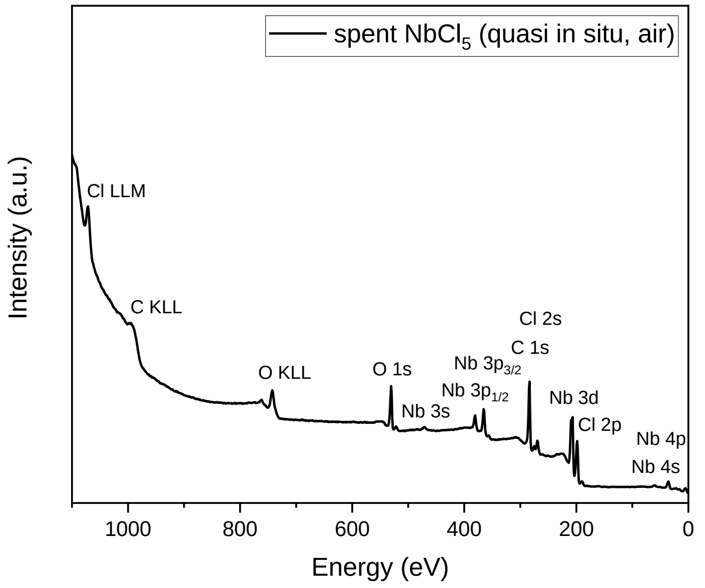

Figure S62: XPS survey spectrum of inert handled spent $NbCl_5$ (referenced to Nb $3d_{5/2}$ = 207.9 eV for $NbCl_5$) in a quasi in situ measurement (after ca. 1.5 h cool-down under N_2 flow and evacuation, stored in vacuum for 96 h) after gas-phase cyclotrimerization of acetylene to benzene (C_2H_2/N_2 1:49, 120 °C, 1 atm, 1 h, WHSV ca. 30 000 $cm^3\ h^{-1}\ g_{cat}^{-1}$) and contact to air (ca. 5 min).

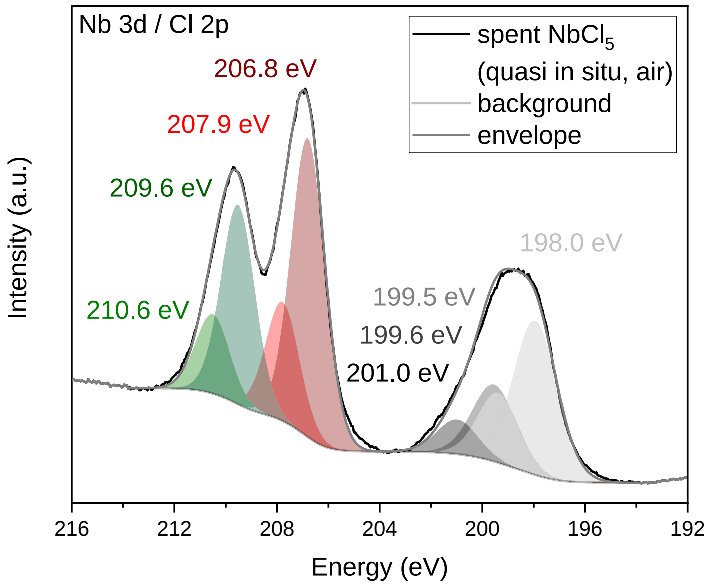

Figure S63: XPS spectrum of Nb 3d ($3d_{3/2}$ and $3d_{5/2}$) and Cl 2p ($2p_{1/2}$ and $2p_{3/2}$) region (referenced to Nb $3d_{5/2}$ = 207.9 eV for $NbCl_5$) of inert handled spent $NbCl_5$ in a quasi in situ measurement (after ca. 1.5 h cool-down under N_2 flow and evacuation, stored in vacuum for 96 h) after gas-phase cyclotrimerization of acetylene to benzene (C_2H_2/N_2 1:49, 120 °C, 1 atm, 1 h, WHSV ca. 30 000 $cm^3\ h^{-1}\ g_{cat}^{-1}$) and contact to air (ca. 5 min) exhibiting two different Nb species. Specie at higher binding energies most likely belongs to Nb^{5+} bound in $NbCl_5$ while the one at lower energies might originate from Nb^{5+} bound to oxygen from contact to air and two chlorine species.

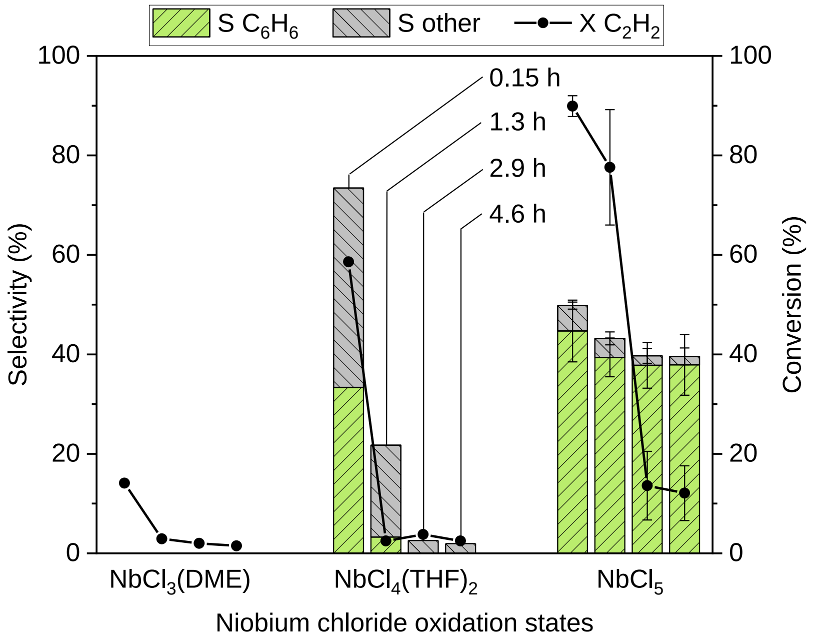

Figure S64: Benzene selectivity, selectivity to other volatiles ("others") and acetylene conversion at different times on stream for niobium chlorides in different oxidation states in the gas-phase cyclotrimerization of acetylene to benzene (C_2H_2/N_2 1:10, 150 °C, 3 bar, WHSV 6 600 cm³ h⁻¹ g_{cat} ⁻¹).

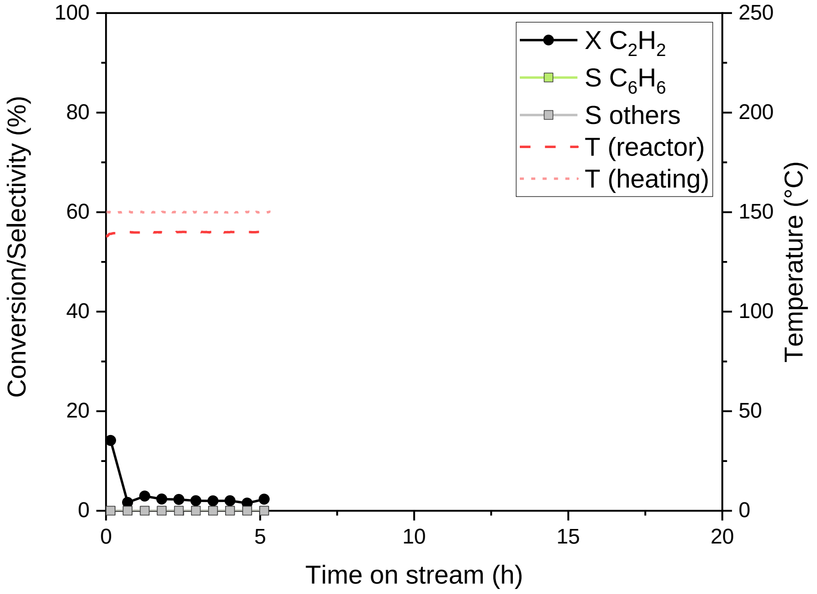

Figure S65: Selectivity to benzene and other volatiles ("others"), acetylene conversion and temperature profiles for $NbCl_3$(DME) in the gas-phase cyclotrimerization of acetylene to benzene (C_2H_2/N_2 1:10, 150 °C, 3 bar, WHSV 6 600 cm³ h⁻¹ g_{cat} ⁻¹).

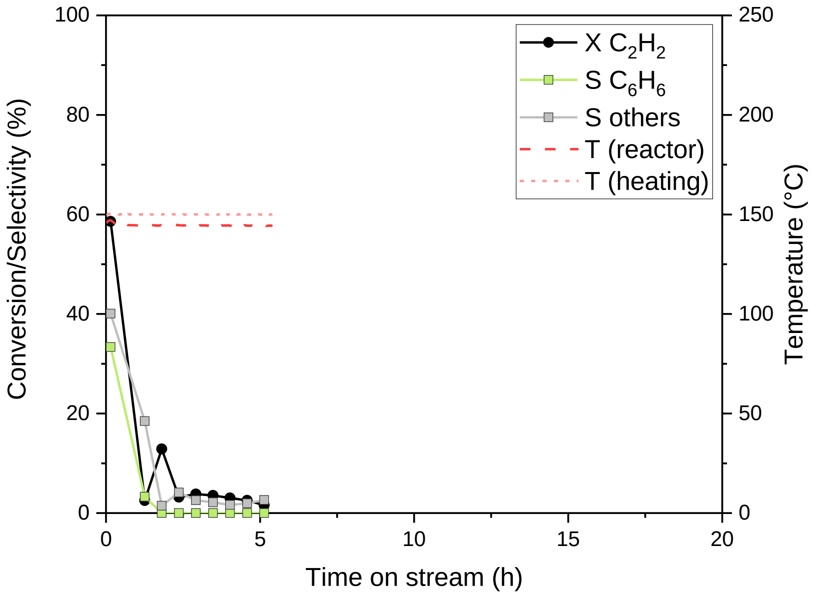

Figure S66: Selectivity to benzene and other volatiles ("others"), acetylene conversion and temperature profiles for $NbCl_4(THF)_2$ in the gas-phase cyclotrimerization of acetylene to benzene (C_2H_2/N_2 1:10, 150 °C, 3 bar, WHSV 6 600 cm³ h⁻¹ g_{cat} ⁻¹).

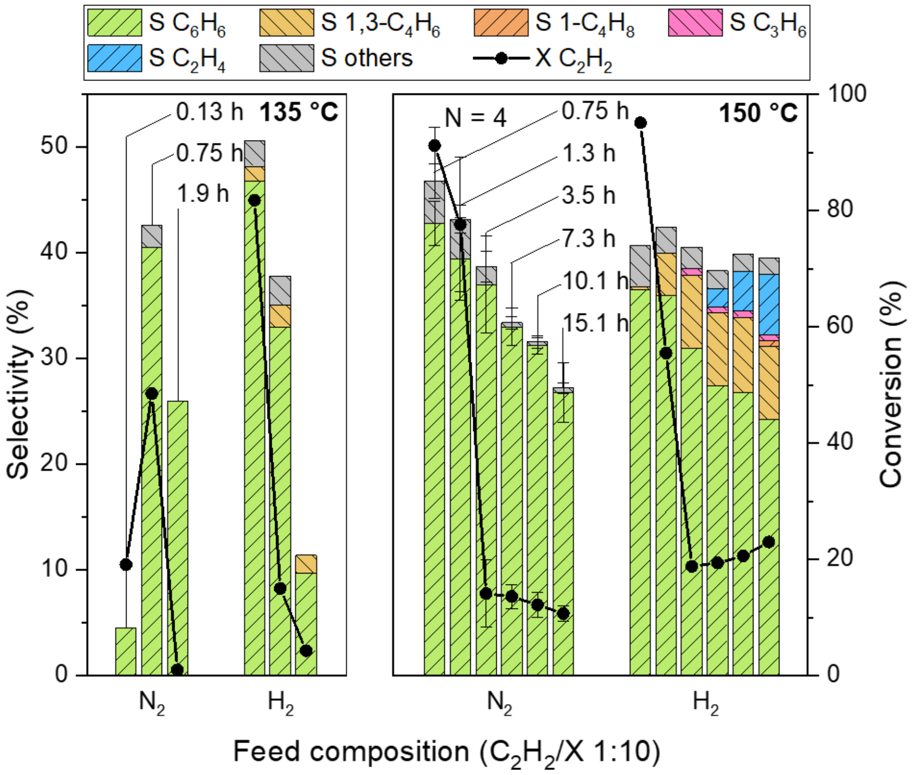

Figure S67: Benzene selectivity, selectivity to 1,3-butadiene, 1-butene, propene, ethylene and other volatiles ("others") and acetylene conversion at different times on stream for $NbCl_5$ in the gas-phase cyclotrimerization of acetylene to benzene with either having nitrogen or hydrogen in the feed at 135 °C (left) and 150 °C (right) (C_2H_2/N_2or H_2 1:10, 3 bar, WHSV 6 600 cm³ h⁻¹ g_{cat} ⁻¹).

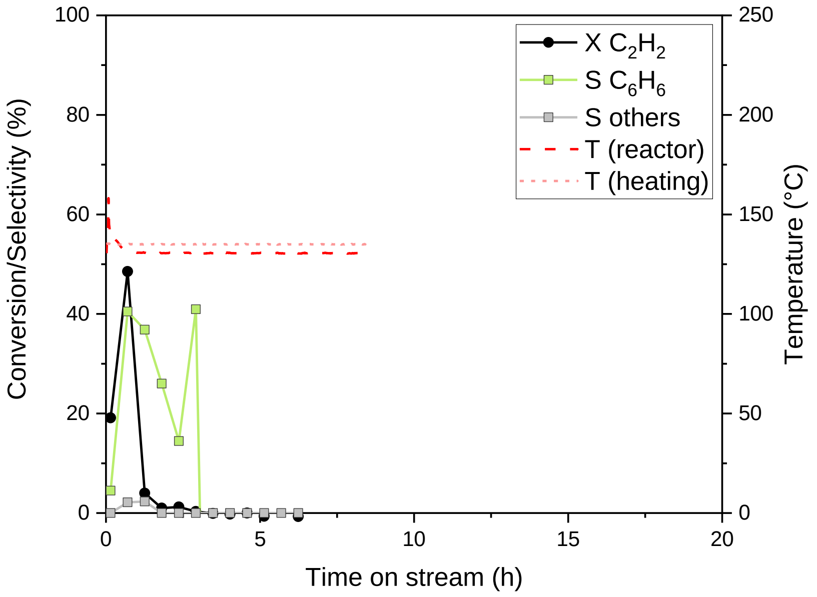

Figure S68: Selectivity to benzene and other volatiles ("others"), acetylene conversion and temperature profiles for $NbCl_5$ in the gas-phase cyclotrimerization of acetylene to benzene (C_2H_2/N_2 1:10, 135 °C, 3 bar, WHSV 6 600 cm³ h⁻¹ g_{cat} ⁻¹).

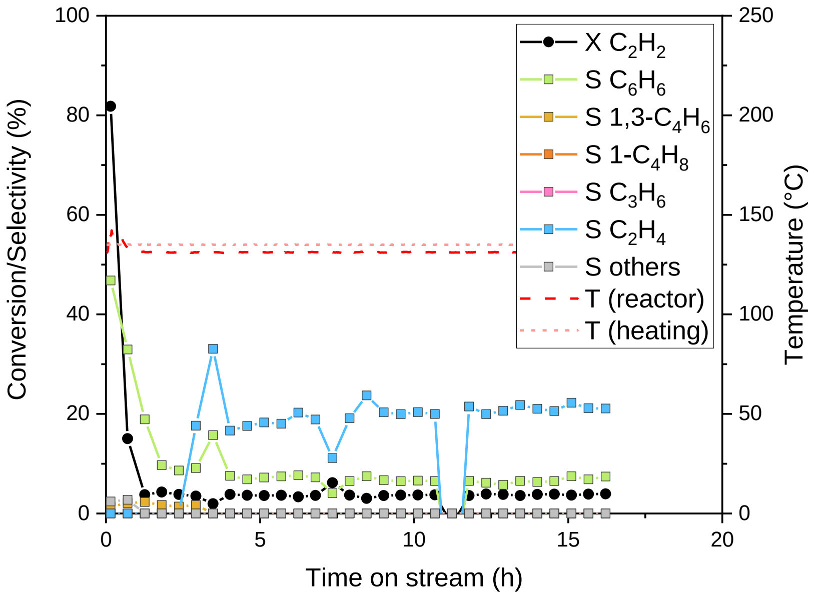

Figure S69: Selectivity to benzene and other volatiles ("others"), acetylene conversion and temperature profiles for $NbCl_5$ in the gas-phase cyclotrimerization of acetylene to benzene replacing nitrogen by hydrogen in the feed (C_2H_2/H_2 1:10, 135 °C, 3 bar, WHSV 6 600 cm^3 h^{-1} g_{cat}^{-1}).

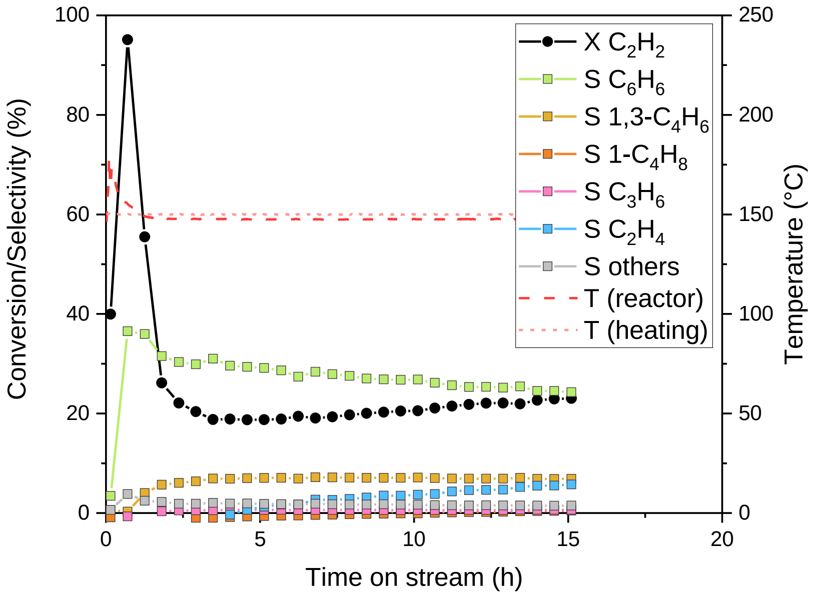

Figure S70: Selectivity to benzene and other volatiles ("others"), acetylene conversion and temperature profiles for $NbCl_5$ in the gas-phase cyclotrimerization of acetylene to benzene replacing nitrogen by hydrogen in the feed (C_2H_2/H_2 1:10, 150 °C, 3 bar, WHSV 6 600 $cm^3\ h^{-1}\ g_{cat}^{-1}$).

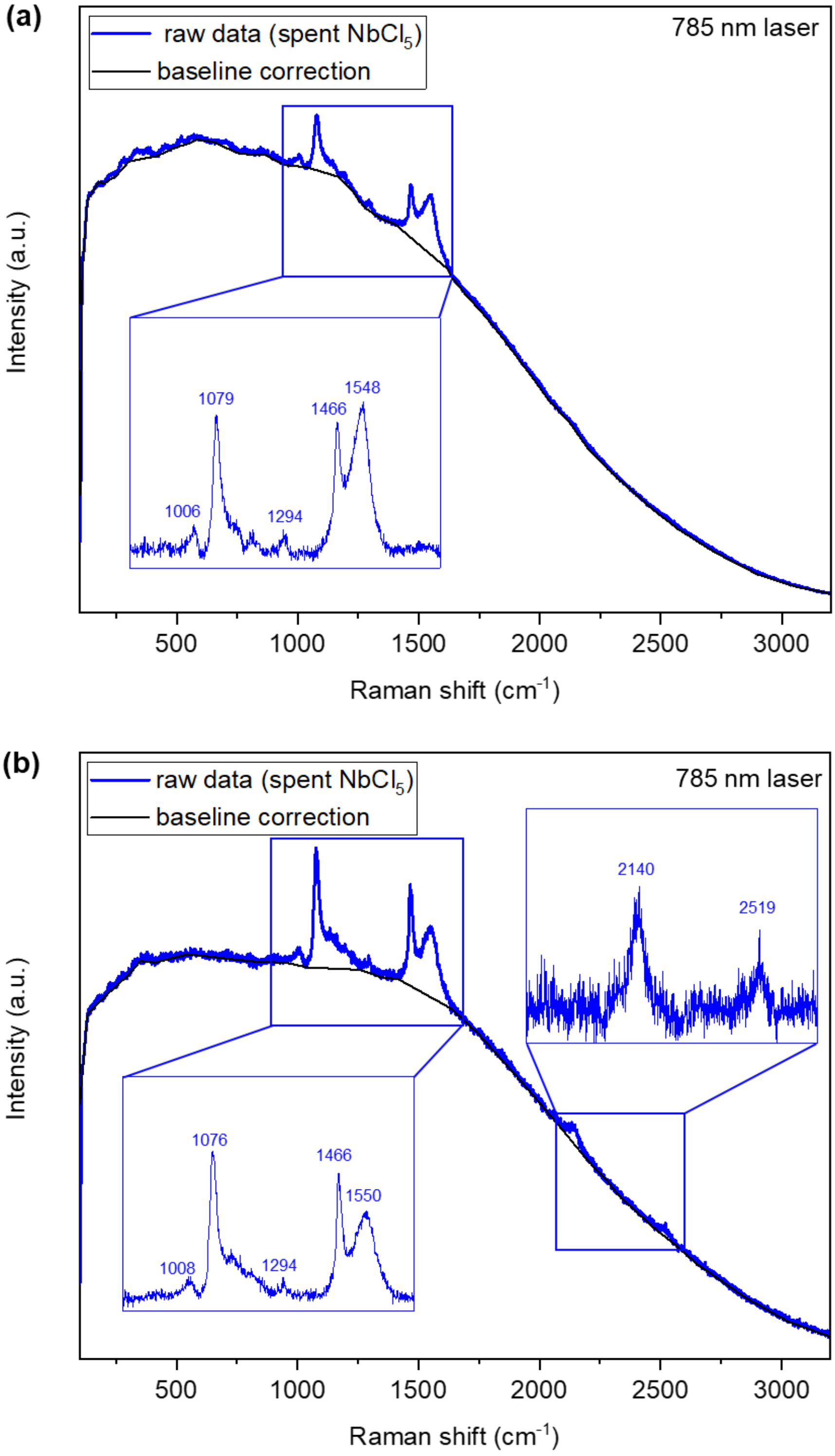

Figure S71: Raman spectrum of the spent $NbCl_5$ catalyst (a) without co-feeding hydrogen (at 150 °C) and (b) after co-feeding hydrogen instead of nitrogen during reaction (C_2H_2/H_2 1:10, 135 °C, 3 bar, WHSV 6 600 cm^3 h^{-1} g_{cat}^{-1}) revealing no substantial effect on polyacetylene formation.

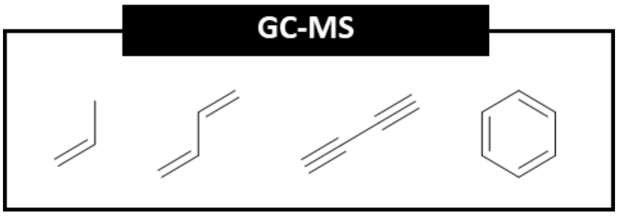

Figure S72: Species detected via GC-MS from product gas stream during gas-phase cyclotrimerization of acetylene to benzene when co-feeding hydrogen ($C_2H_2/C_2H_4/N_2$ 1:7:3, 150 °C, 3 bar, WHSV 6 600 cm³ h⁻¹ g_{cat} ⁻¹) using sampling gasbags or a cold trap in acetone/dry ice at -50 °C at the reactor outlet.

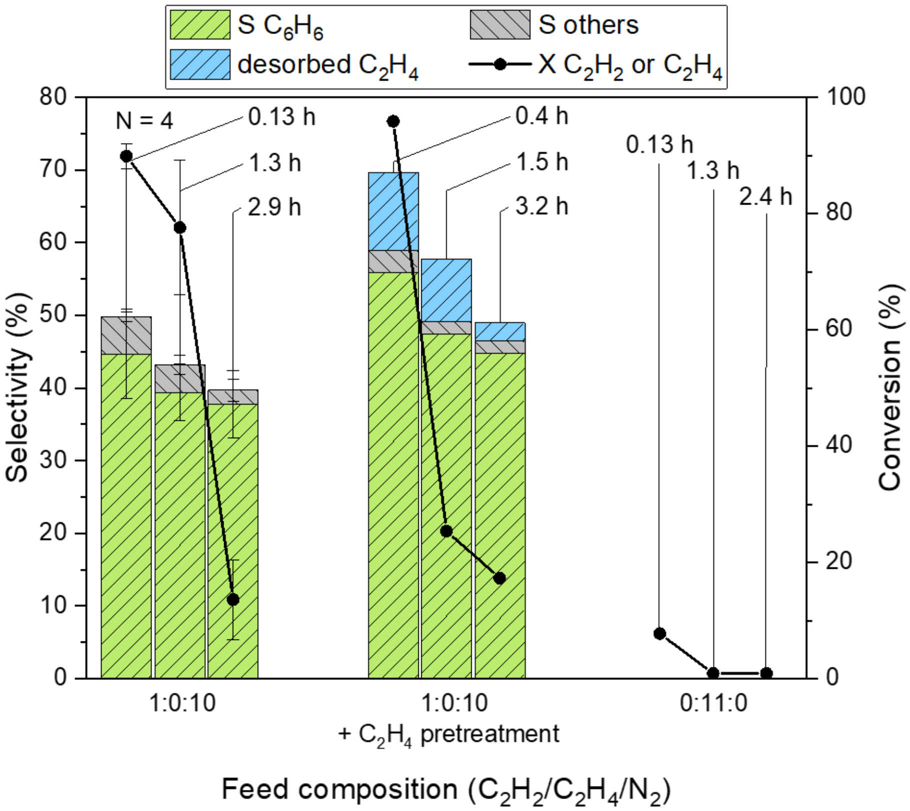

Figure S73: Benzene selectivity, selectivity to other volatiles ("others") and acetylene conversion at different times on stream in the gas-phase cyclotrimerization of acetylene to benzene for non-pretreated $NbCl_5$ (left), for 2.5 h at reaction temperature with pure ethylene (55 mL/min) pretreated $NbCl_5$ (middle) and for non-pretreated $NbCl_5$ only reacted with pure ethylene (right) (C_2H_2/N_2 1:10 or only C_2H_4, 150 °C, 3 bar, WHSV 6 600 cm³ h⁻¹ g_{cat} ⁻¹).

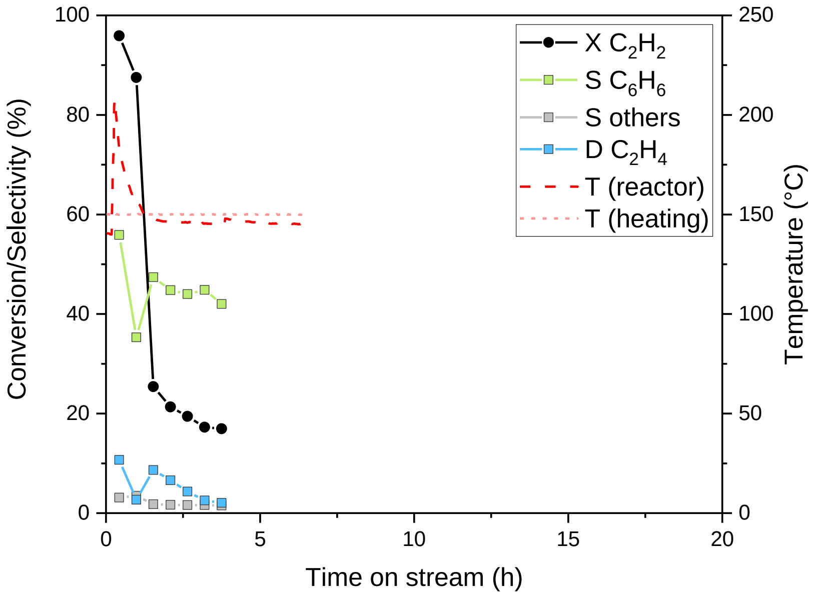

Figure S74: Selectivity to benzene and other volatiles ("others"), desorption of adsorbed ethylene and acetylene conversion and temperature profiles for $NbCl_5$ after a 2.5 h pretreatment with pure ethylene at reaction temperature in the gas-phase cyclotrimerization of acetylene to benzene (C_2H_2/N_2 1:10, 150 °C, 3 bar, WHSV 6 600 $cm^3\ h^{-1}\ g_{cat}{}^{-1}$).

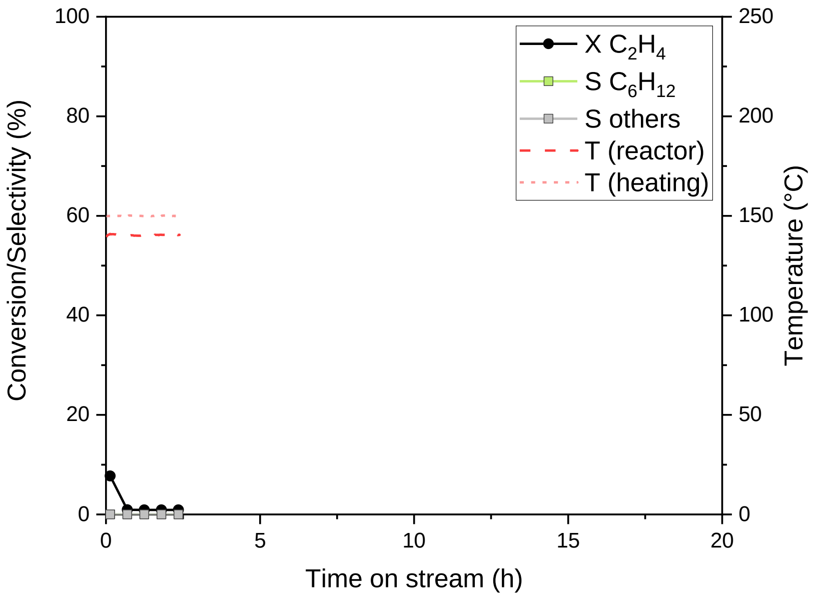

Figure S75: Selectivity to cyclohexane and other volatiles ("others"), ethylene conversion and temperature profiles for $NbCl_5$ in the gas-phase cyclotrimerization of ethylene to cyclohexane (C_2H_2/N_2 1:10, 150 °C, 3 bar, WHSV 6 600 $cm^3\ h^{-1}\ g_{cat}{}^{-1}$).

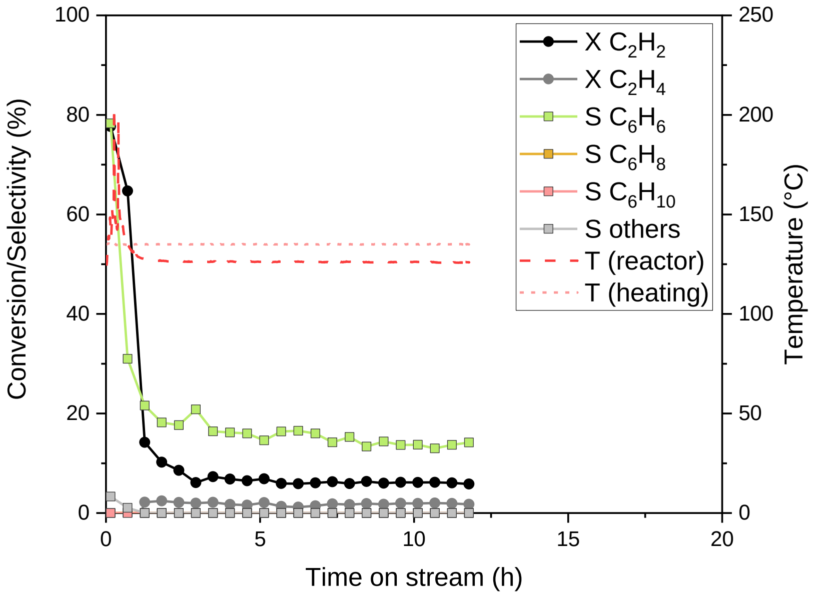

Figure S76: Benzene selectivity, selectivity to 1,3-cyclohexadiene, cyclohexene, other volatiles ("others"), acetylene conversion, ethylene conversion and temperature profiles for $NbCl_5$ in the gas-phase cyclotrimerization of acetylene to benzene upon C_2H_4 co-feed ($C_2H_2/C_2H_4/N_2$ 1:1:9, 135 °C, 3 bar, WHSV 6 600 $cm^3\ h^{-1}\ g_{cat}^{-1}$). The selectivity to 1,3-cyclohexadiene and cyclohexene was calculated based on the amounts of acetylene molecules involved during cocyclization being 2 and 1, respectively.

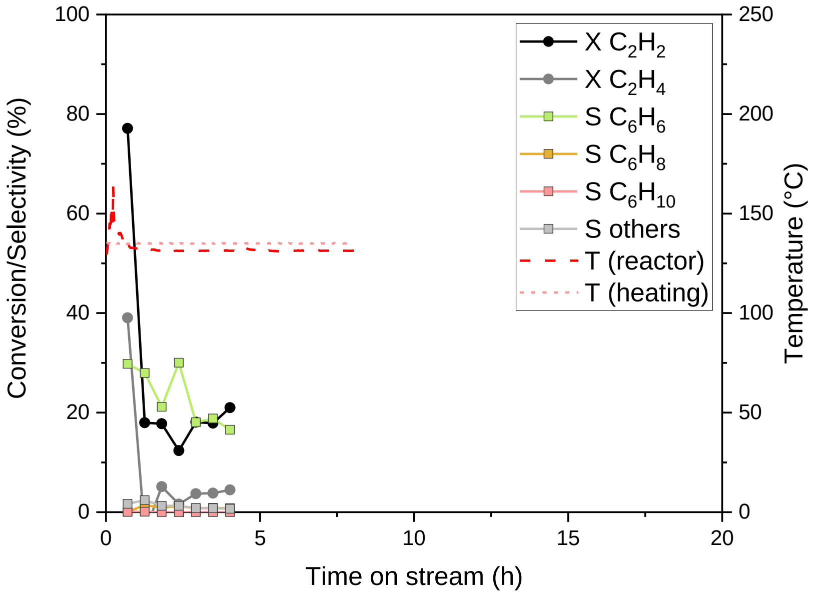

Figure S77: Benzene selectivity, selectivity to 1,3-cyclohexadiene, cyclohexene, other volatiles ("others"), acetylene conversion, ethylene conversion and temperature profiles for $NbCl_5$ in the gas-phase cyclotrimerization of acetylene to benzene upon C_2H_4 co-feed ($C_2H_2/C_2H_4/N_2$ 1:3:7, 135 °C, 3 bar, WHSV 6 600 $cm^3\ h^{-1}\ g_{cat}^{-1}$). Starting from 3 h on stream the pressure started to slowly increase due to reactor blocking to 4 bar after 4 h on stream and the reaction was stopped. The selectivity to 1,3-cyclohexadiene and cyclohexene was calculated based on the amounts of acetylene molecules involved during cocyclization being 2 and 1, respectively.

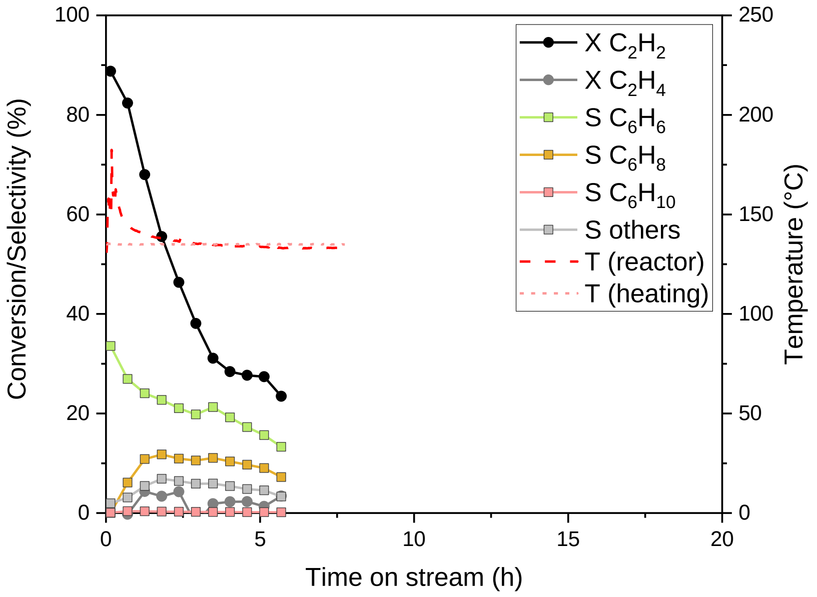

Figure S78: Benzene selectivity, selectivity to 1,3-cyclohexadiene, cyclohexene, other volatiles ("others"), acetylene conversion, ethylene conversion and temperature profiles for $NbCl_5$ in the gas-phase cyclotrimerization of acetylene to benzene upon C_2H_4 co-feed ($C_2H_2/C_2H_4/N_2$ 1:5:5, 135 °C, 3 bar, WHSV 6 600 $cm^3\ h^{-1}\ g_{cat}{}^{-1}$). The selectivity to 1,3-cyclohexadiene and cyclohexene was calculated based on the amounts of acetylene molecules involved during cocyclization being 2 and 1, respectively.

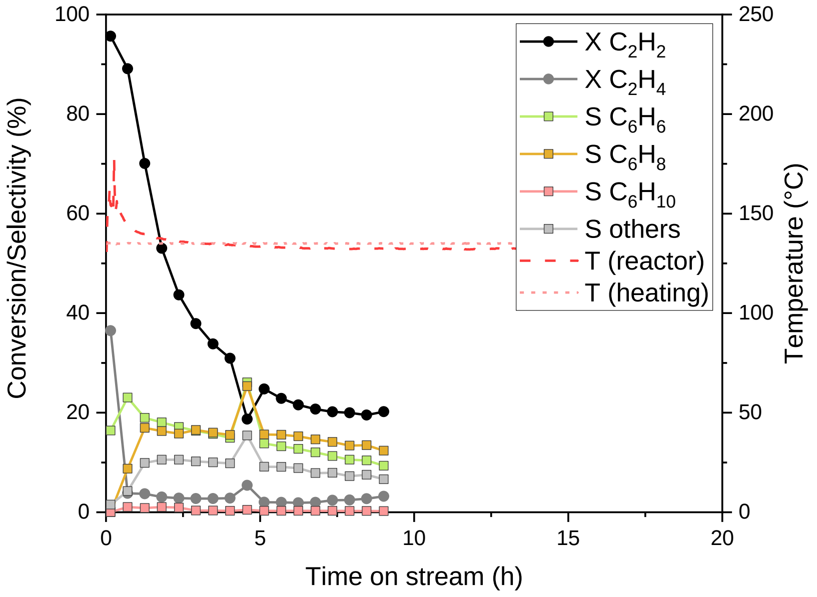

Figure S79: Benzene selectivity, selectivity to 1,3-cyclohexadiene, cyclohexene, other volatiles ("others"), acetylene conversion, ethylene conversion and temperature profiles for $NbCl_5$ in the gas-phase cyclotrimerization of acetylene to benzene upon C_2H_4 co-feed ($C_2H_2/C_2H_4/N_2$ 1:10:0, 135 °C, 3 bar, WHSV 6 600 $cm^3\ h^{-1}\ g_{cat}{}^{-1}$). The selectivity to 1,3-cyclohexadiene and cyclohexene was calculated based on the amounts of acetylene molecules involved during cocyclization being 2 and 1, respectively.

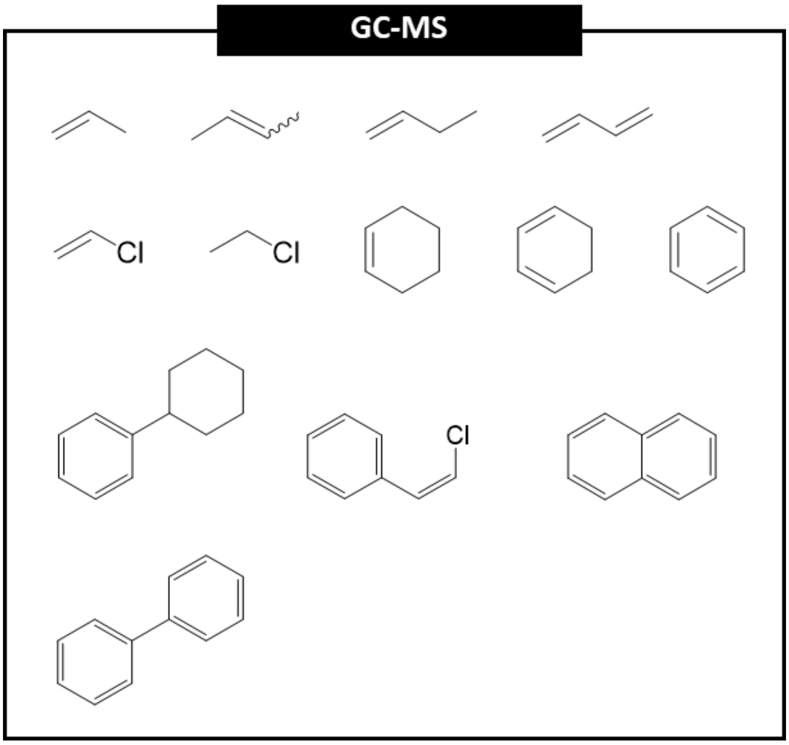

Figure S80: Species detected via GC-MS from product gas stream during gas-phase cyclotrimerization of acetylene to benzene co-feeding ethylene ($C_2H_2/C_2H_4/N_2$ 1:3-5:7-5, 135 °C, 3 bar, WHSV 6 600 $cm^3\ h^{-1}\ g_{cat}^{-1}$) using sampling gasbags or a cold trap in acetone/dry ice at -50 °C at the reactor outlet.

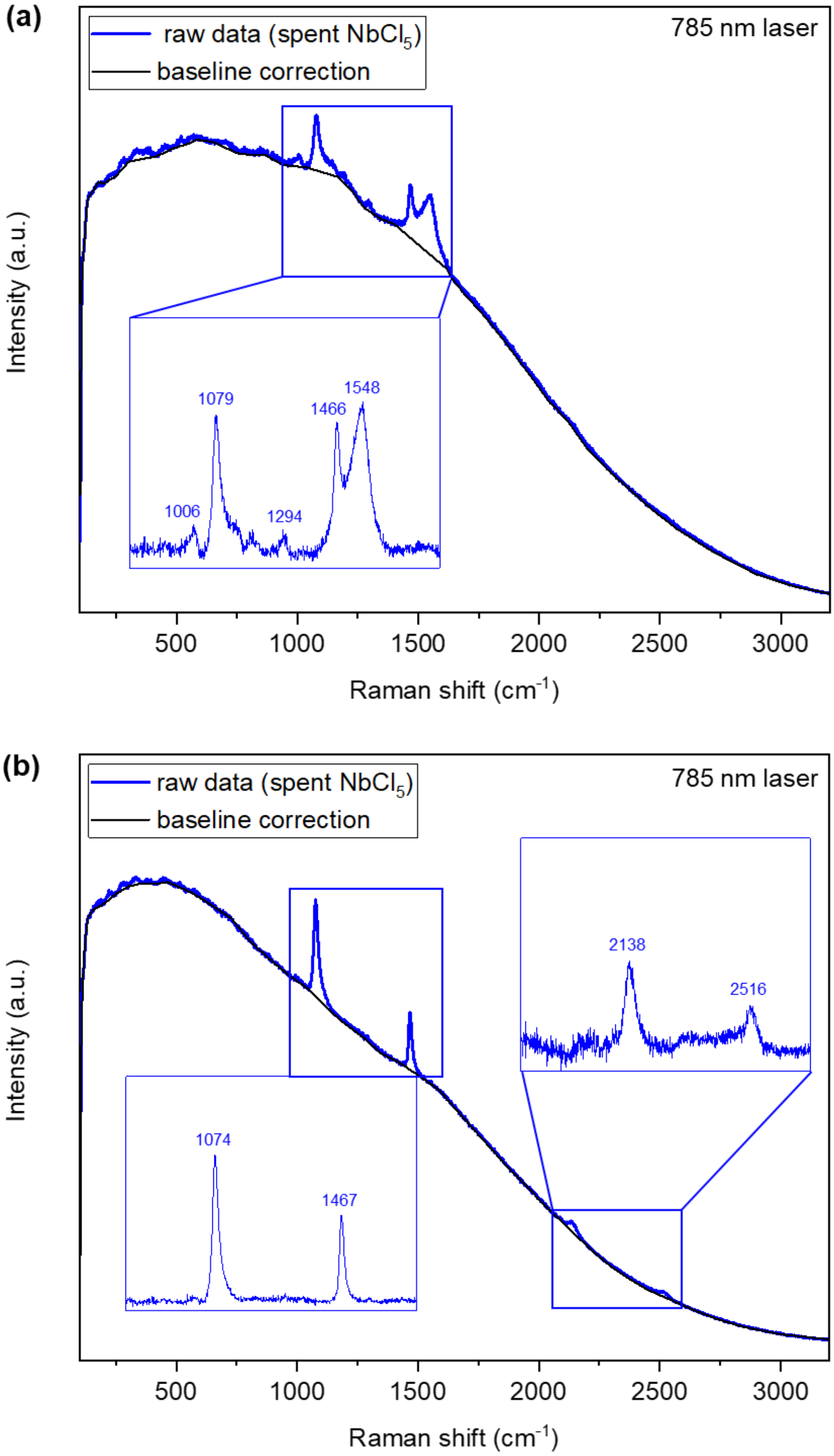

Figure S81: Raman spectrum of the spent $NbCl_5$ catalyst (a) without co-feeding ethylene (at 150 °C) and (b) after co-feeding ethylene instead of nitrogen during reaction (C_2H_2/C_2H_4 1:10, 135 °C, 3 bar, WHSV 6 600 $cm^3\ h^{-1}\ g_{cat}^{-1}$) revealing more defect-free polyacetylene formation with sharper Raman bands and even visible overtones.

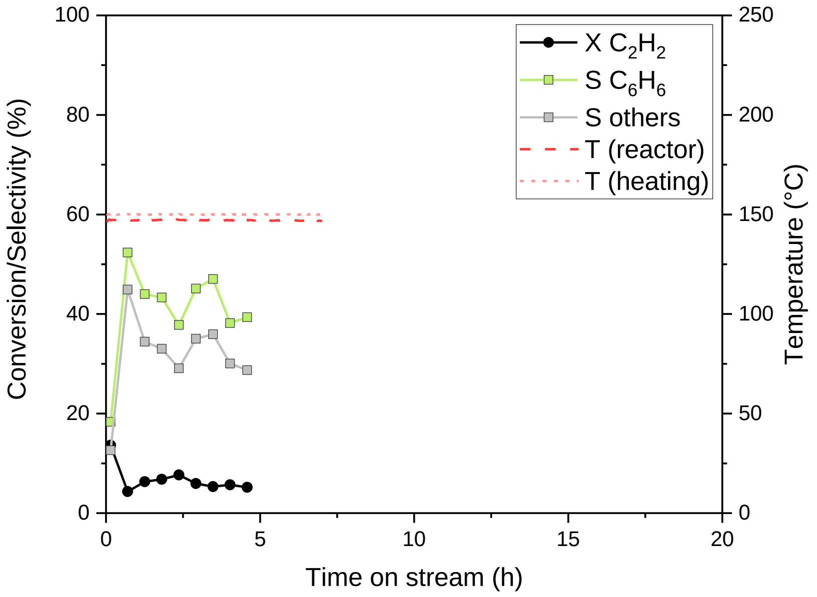

Figure S82: Selectivity to benzene and other volatiles ("others"), acetylene conversion and temperature profiles for NbI_5 in the gas-phase cyclotrimerization of acetylene to benzene (C_2H_2/N_2 1:10, 150 °C, 3 bar, WHSV 6 600 cm³ h⁻¹ g_{cat}^{-1}).

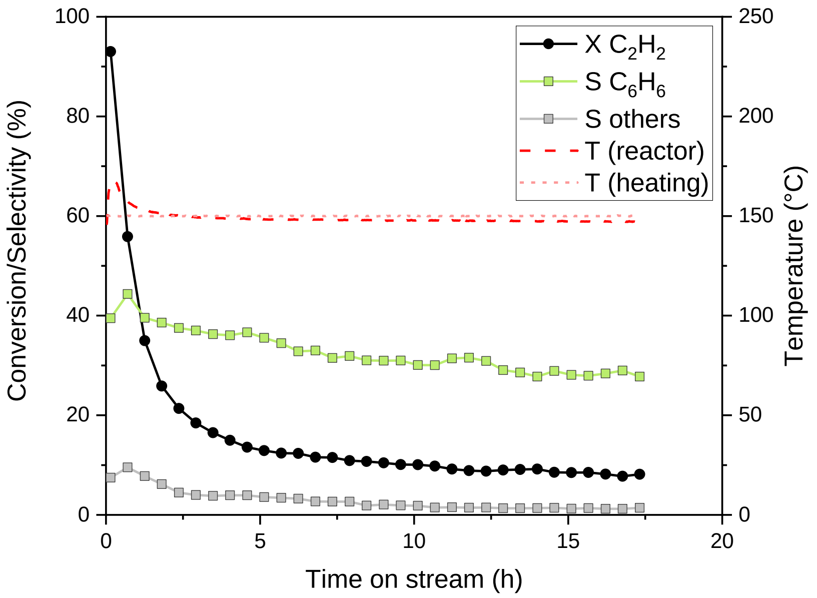

Figure S83: Selectivity to benzene and other volatiles ("others"), acetylene conversion and temperature profiles for $NbBr_5$ in the gas-phase cyclotrimerization of acetylene to benzene (C_2H_2/N_2 1:10, 150 °C, 3 bar, WHSV 6 600 cm³ h⁻¹ g_{cat}^{-1}).

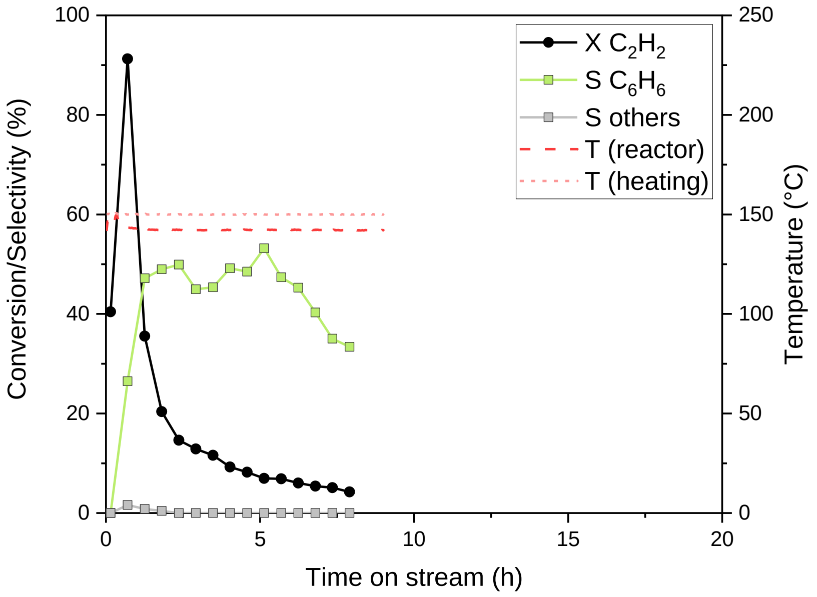

Figure S84: Selectivity to benzene and other volatiles ("others"), acetylene conversion and temperature profiles for NbF_5 in the gas-phase cyclotrimerization of acetylene to benzene (C_2H_2/N_2 1:10, 150 °C, 3 bar, WHSV 6 600 $cm^3\ h^{-1}\ g_{cat}^{-1}$).

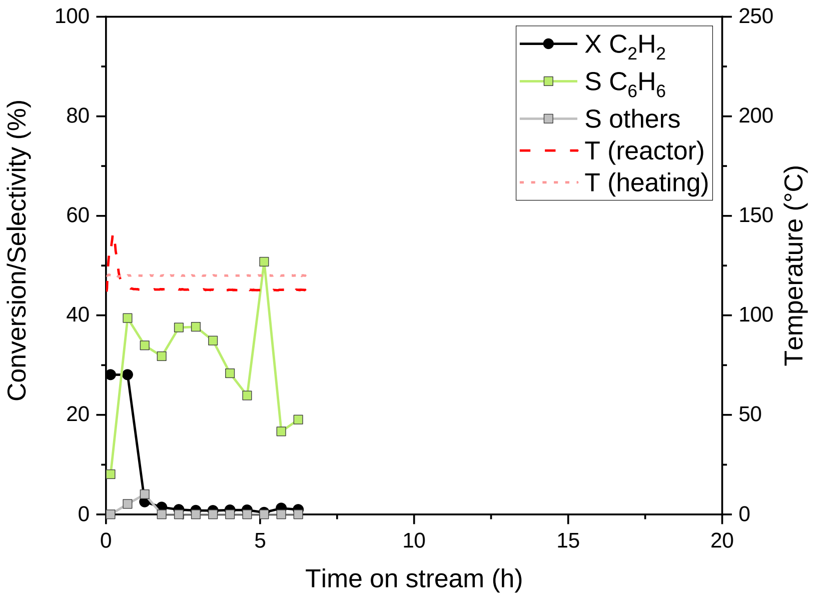

Figure S85: Selectivity to benzene and other volatiles ("others"), acetylene conversion and temperature profiles for $NbCl_5$ in the gas-phase cyclotrimerization of acetylene to benzene (C_2H_2/N_2 1:10, 120 °C, 3 bar, WHSV 6 600 $cm^3\ h^{-1}\ g_{cat}^{-1}$).

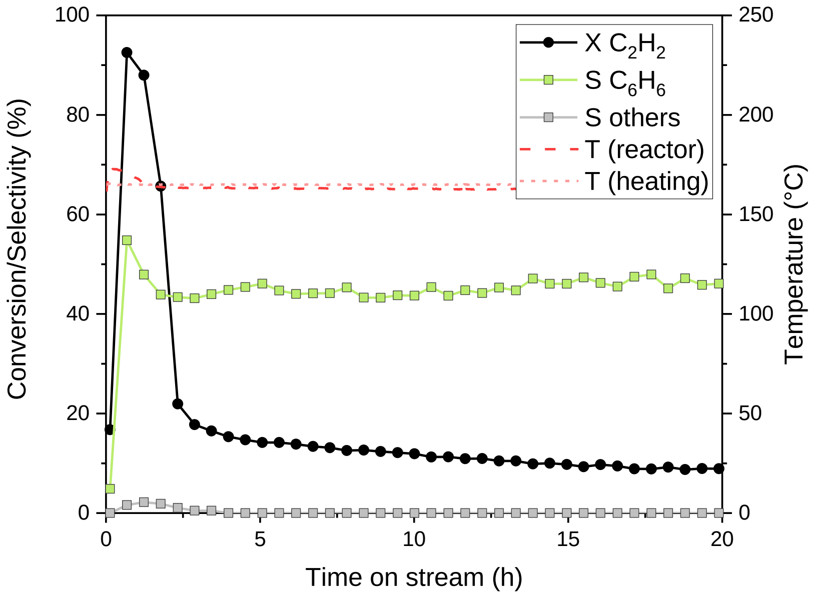

Figure S86: Selectivity to benzene and other volatiles ("others"), acetylene conversion and temperature profiles for $NbCl_5$ in the gas-phase cyclotrimerization of acetylene to benzene (C_2H_2/N_2 1:10, 165 °C, 3 bar, WHSV 6 600 $cm^3\ h^{-1}\ g_{cat}^{\ -1}$).

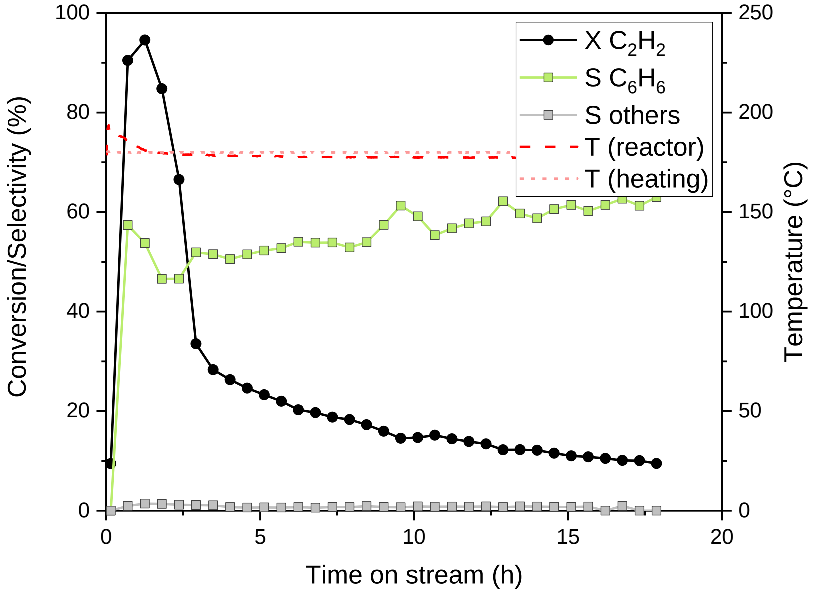

Figure S87: Selectivity to benzene and other volatiles ("others"), acetylene conversion and temperature profiles for $NbCl_5$ in the gas-phase cyclotrimerization of acetylene to benzene (C_2H_2/N_2 1:10, 180 °C, 3 bar, WHSV 6 600 $cm^3\ h^{-1}\ g_{cat}^{\ -1}$).

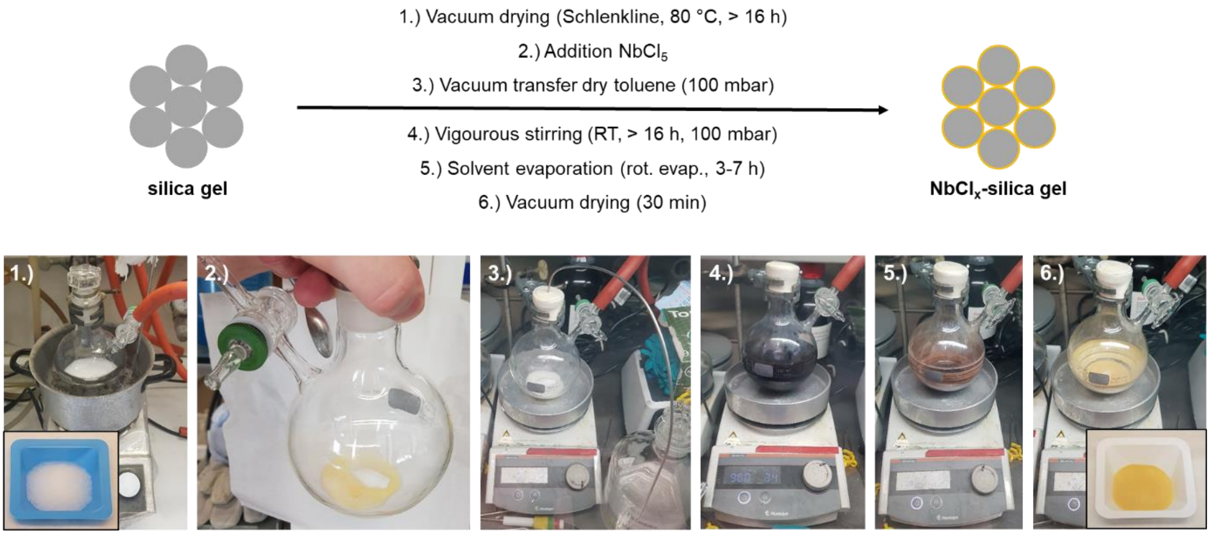

Figure S88: Scheme of the synthesis procedure for the chemical grafting of $NbCl_x$ species to a mesoporous silica gel support via the reaction of $NbCl_5$ dissolved in toluene with the surface hydroxyl groups of the silica gel material at room temperature. The $NbCl_x$-functionalized silica gel materials were obtained after solvent removal and careful drying.

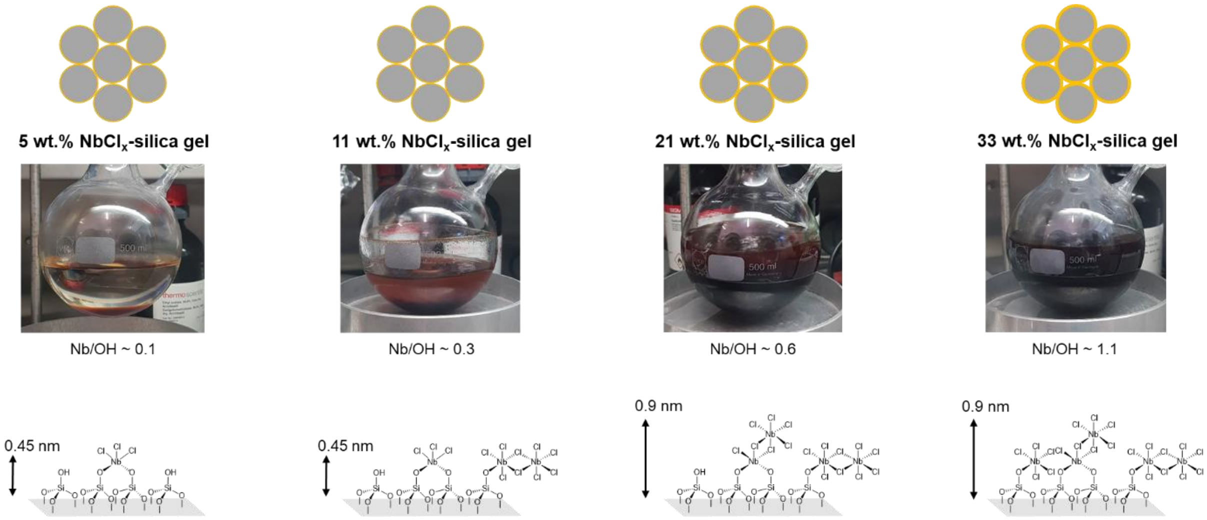

Figure S89: Synthesis procedure for the chemical grafting of $NbCl_x$ species to a mesoporous silica gel support via the reaction of $NbCl_5$ dissolved in toluene with the surface hydroxyl groups of the silica gel material performed for different molar niobium to surface hydroxyl group ratios, achieving $NbCl_x$ loadings between 5 and 33 wt.%. In case of the lowest loading the reaction solution completely discolored, demonstrating a quantitative surface reaction to mono- and/or bipodal Nb(-O-)$_{1-2}$-Si species as reported in literature.[12, 13]

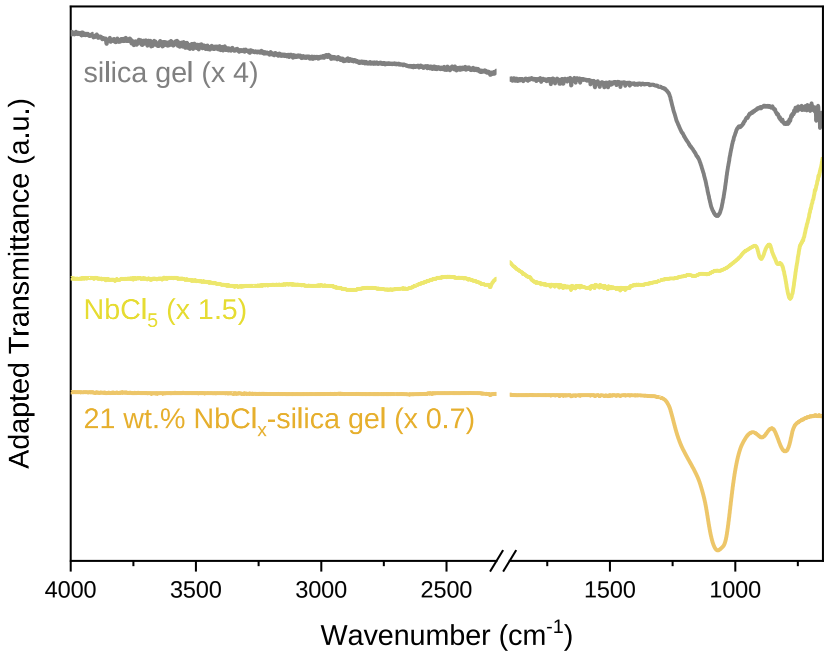

Figure S90: FTIR spectroscopy of the pristine silica gel, bulk $NbCl_5$ and the 21 wt.% $NbCl_x$-silica gel material after synthesis with band assignments according to literature[13, 14].

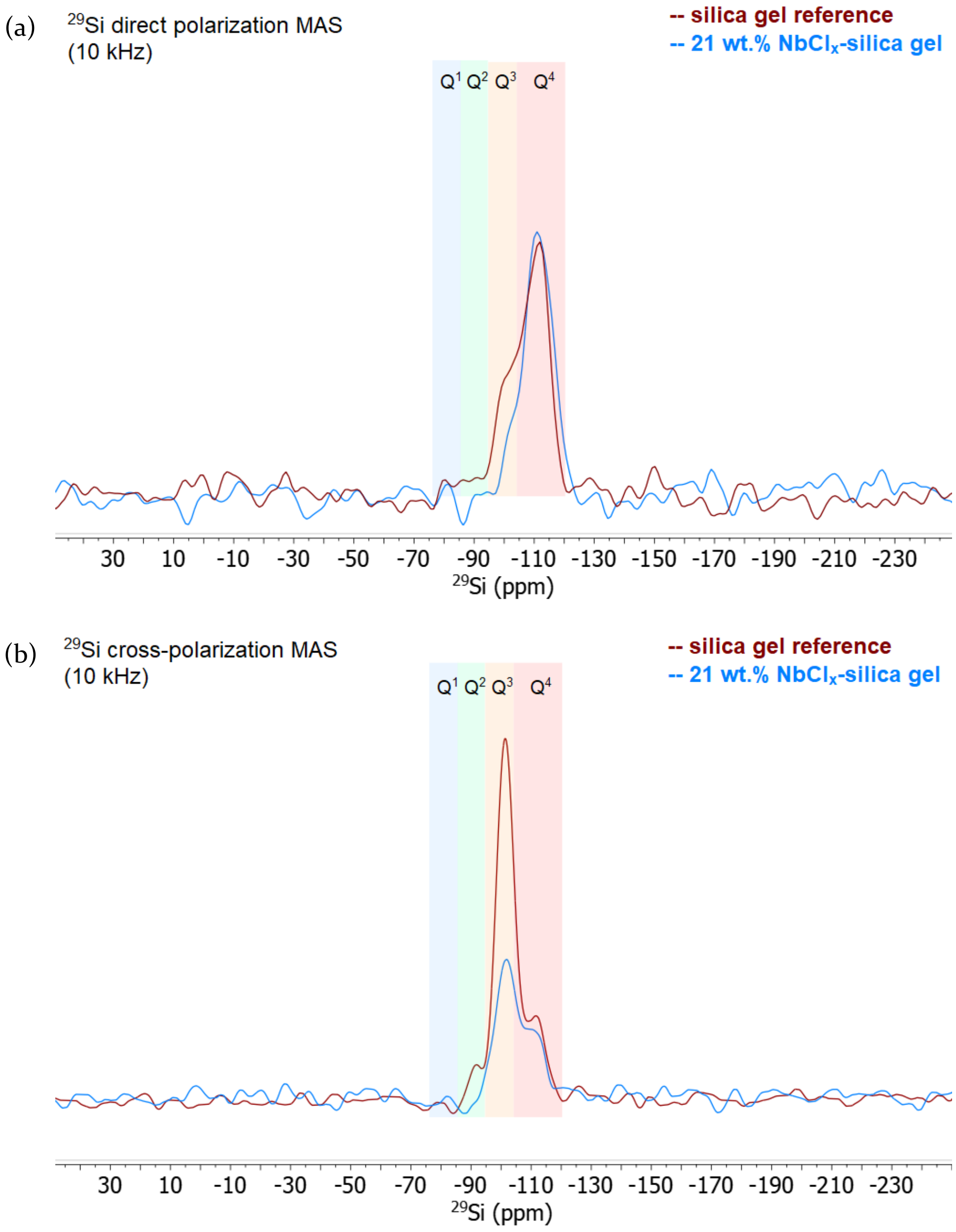

Figure S91: ^{29}Si *ss*NMR spectra of the 21 wt.% $NbCl_x$-silica gel (blue) and the pristine silica gel (red) measured by (a) direct polarization and by (b) $^{1}H \rightarrow {}^{29}Si$ cross-polarization under MAS condition at 10kHz. The chemical shift ranges for the different silicon Q^n species (with n = number of bridging oxygen atoms) are indicated. A marked reduction of the region between 96-103 ppm corresponding to the Q^3 silanol species is observed, indicating a decreased share of single silanol functionalities on the silica gel surface after functionalization.

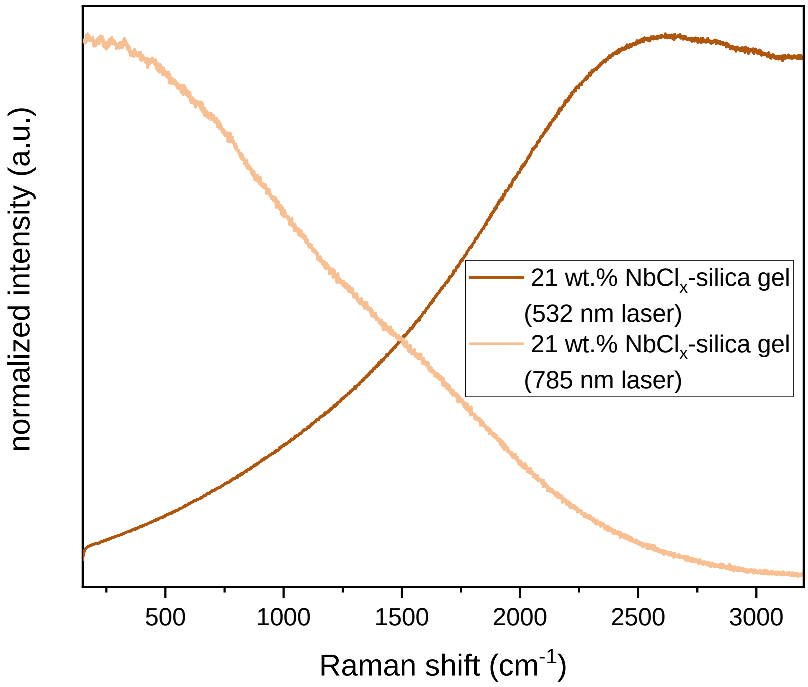

Figure S92: Raman spectroscopy of the 21 wt.% NbCl$_x$-silica gel material using different laser wavelengths. The signal-to-fluorescence ratio is so low that independent of the laser wavelength no bands are observable.

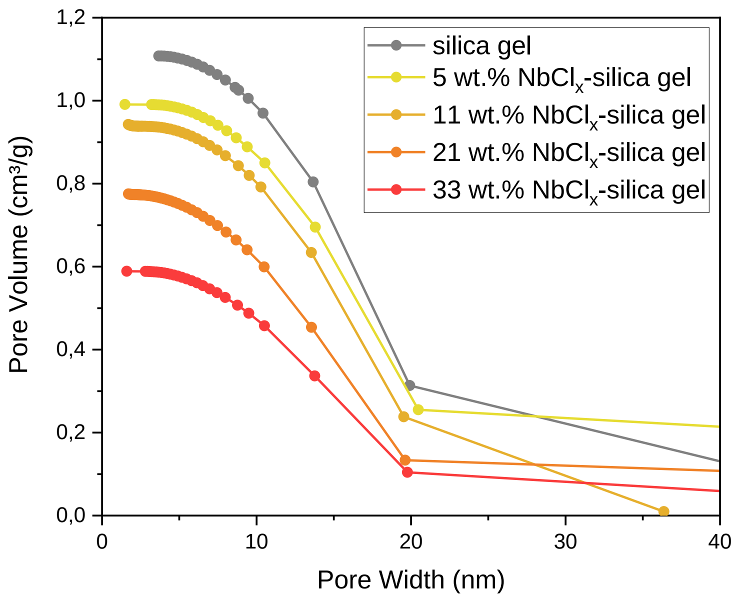

Figure S93: Cumulative pore volume vs. pore size plot derived from the N_2 physisorption data using BJH theory for the pristine silica gel and the NbCl$_x$-functionalized silica gels.

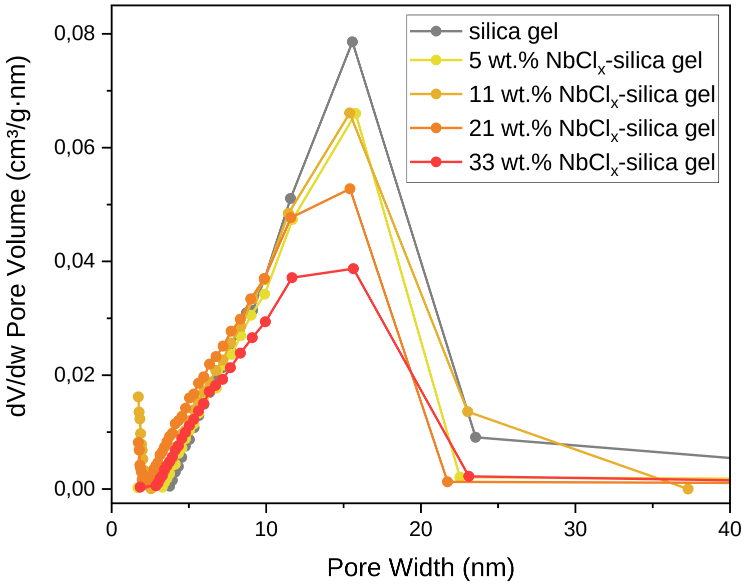

Figure S94: Pore size distribution for the pristine silica gel and the $NbCl_x$-functionalized silica gel materials derived from N_2 physisorption data using BJH theory.

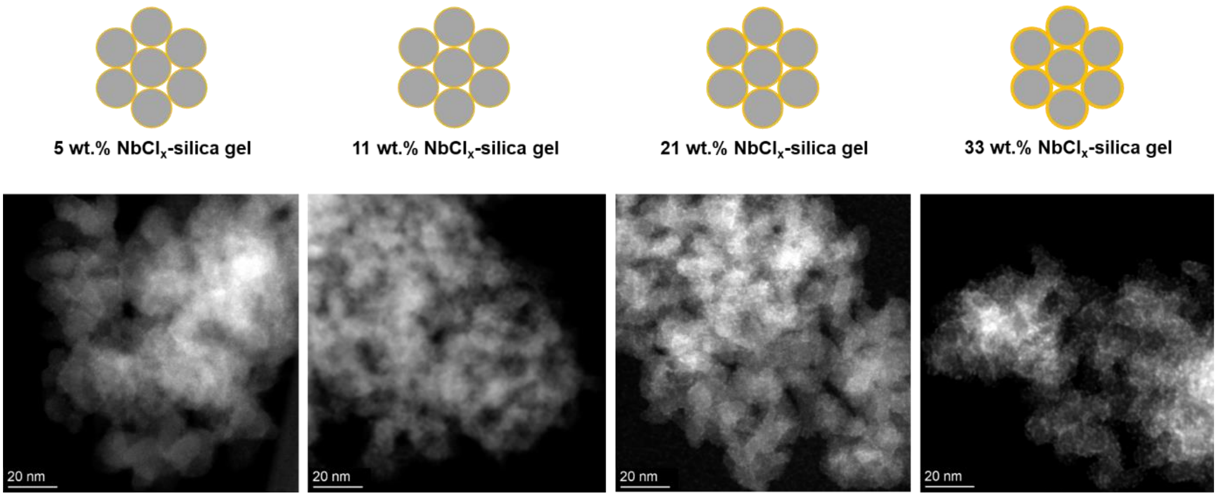

Figure S95: TEM images of the $NbCl_x$ functionalized silica gel materials comparing different loadings. Only in the case of the highest loading ca. 1 nm thin coatings of the $NbCl_x$ moieties on the surface of the silica particles are visible after short irradiation times with the electron beam.

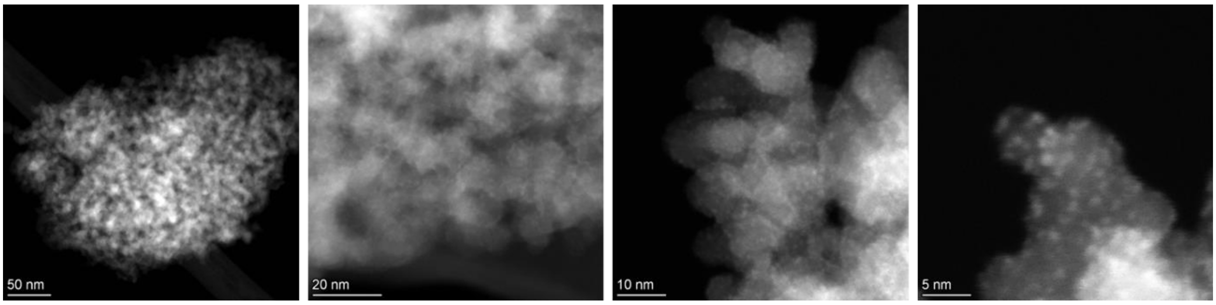

Figure S96: TEM images of the 5 wt.% $NbCl_x$-silica gel material with increasing magnification. Upon longer irradiation under the electron beam small nanoagglomerates of $NbCl_x$ become visible.

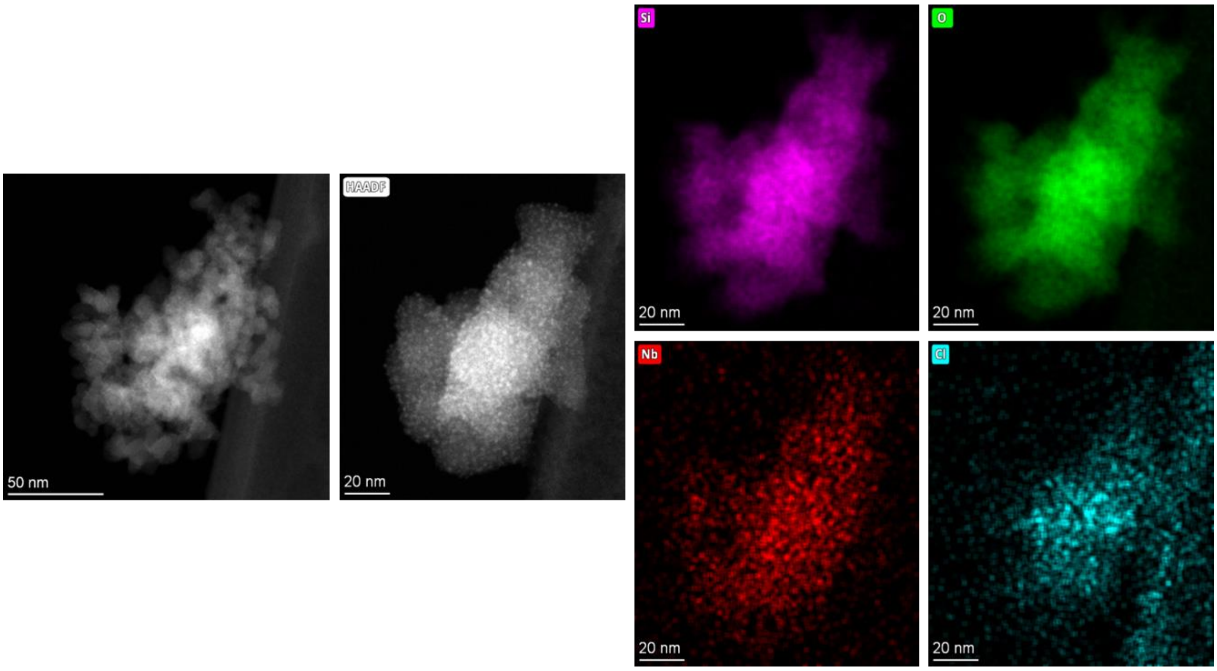

Figure S97: TEM images with elemental mapping of the 5 wt.% $NbCl_x$-silica gel material revealing the fine dispersion of $NbCl_x$ species over the silica gel support. Upon longer irradiation under the electron beam during measurement small nanoagglomerates of $NbCl_x$ become visible.

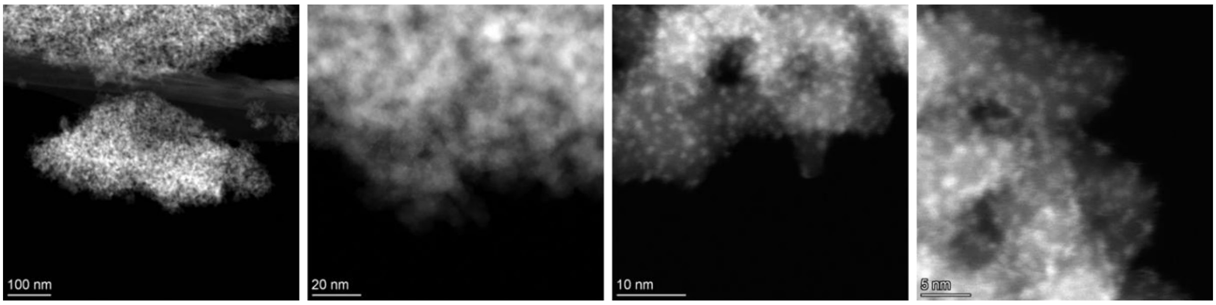

Figure S98: TEM images of the 11 wt.% $NbCl_x$-silica gel material with increasing magnification. Upon longer irradiation under the electron beam small nanoagglomerates of $NbCl_x$ become visible.

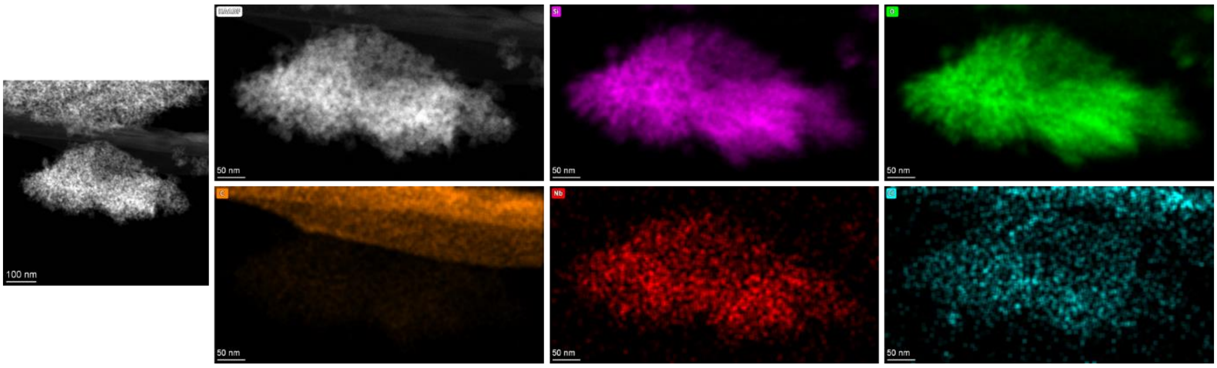

Figure S99: TEM images with elemental mapping of the 11 wt.% $NbCl_x$-silica gel material revealing the fine dispersion of $NbCl_x$ species over the silica gel support.

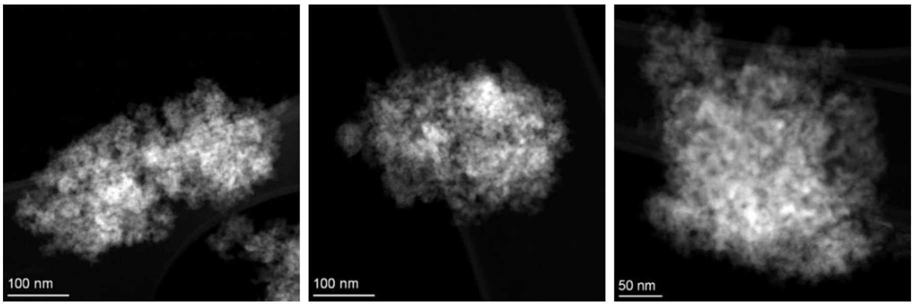

Figure S100: TEM images of the 21 wt.% $NbCl_x$-silica gel material with increasing magnification.

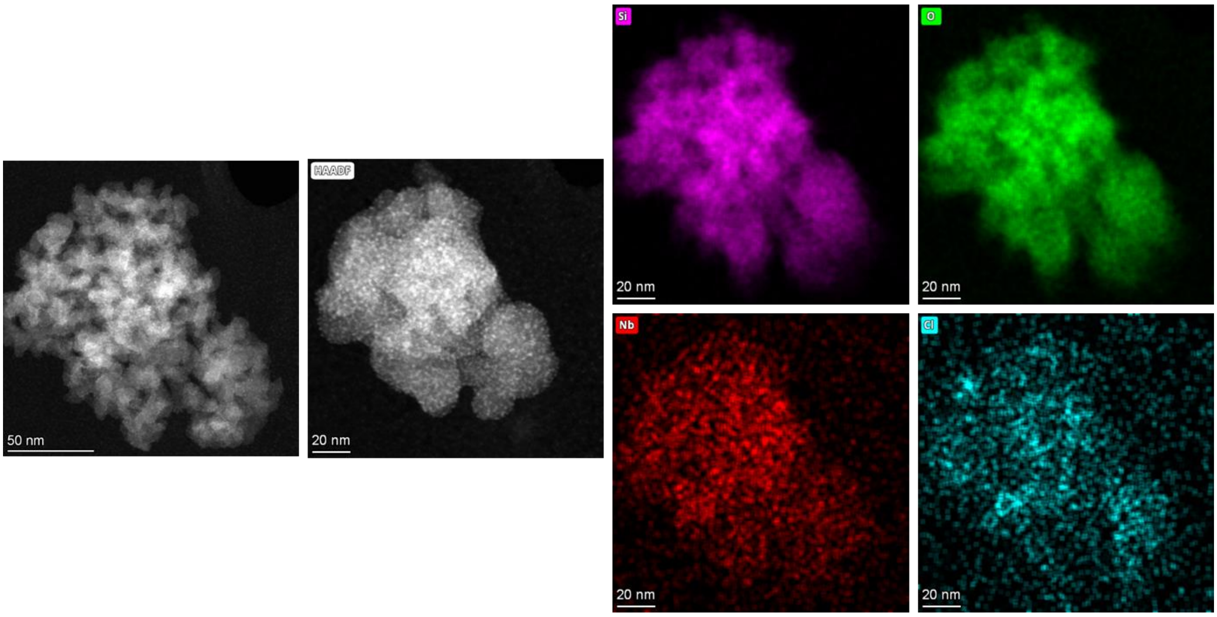

Figure S101: TEM images with elemental mapping of the 21 wt.% $NbCl_x$-silica gel material revealing the fine dispersion of $NbCl_x$ species over the silica gel support. Upon longer irradiation under the electron beam small nanoagglomerates of $NbCl_x$ become visible.

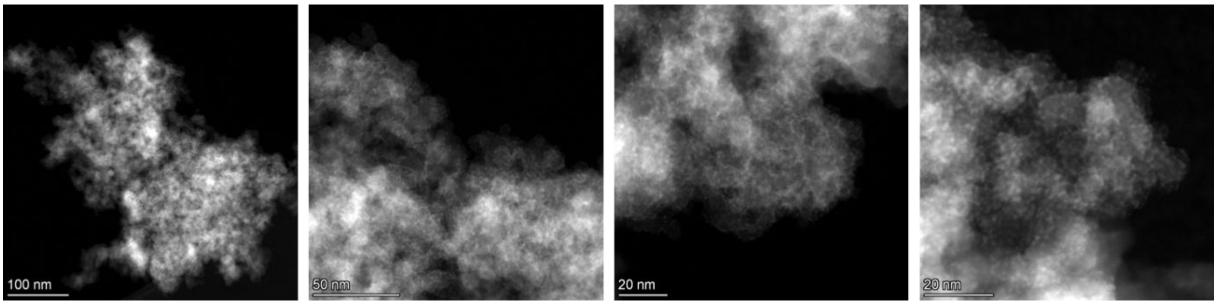

Figure S102: TEM images of the 33 wt.% $NbCl_x$-silica gel material with increasing magnification. Surface coating of the silica gel support is visible as a ca. 1 nm thick layer. Partially also much larger agglomerates are visible.

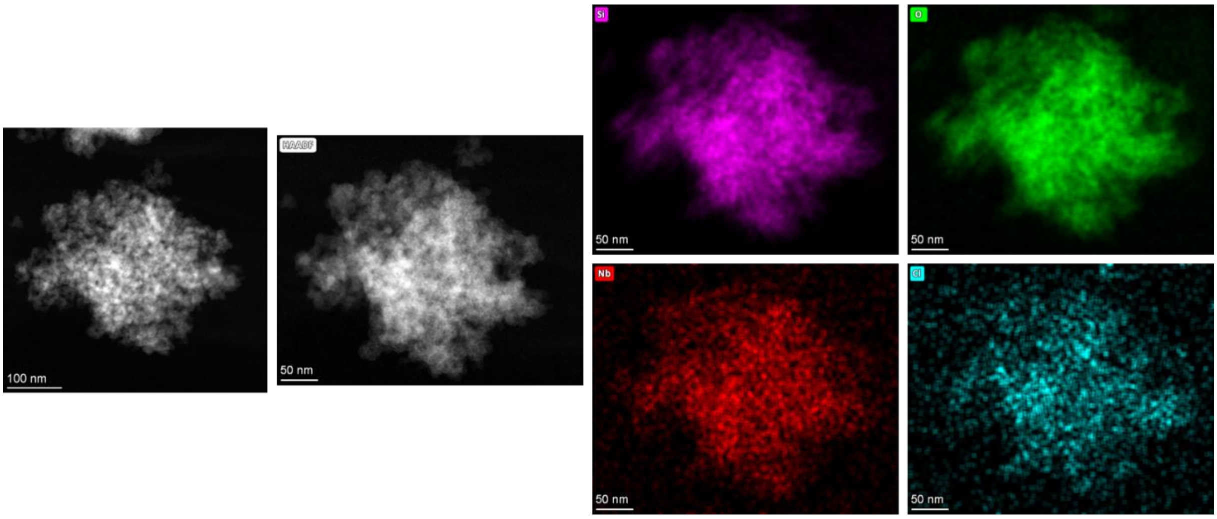

Figure S103: TEM images with elemental mapping of the 33 wt.% $NbCl_x$-silica gel material revealing the fine dispersion of $NbCl_x$ species over the silica gel support. Partially also larger agglomerates are visible.

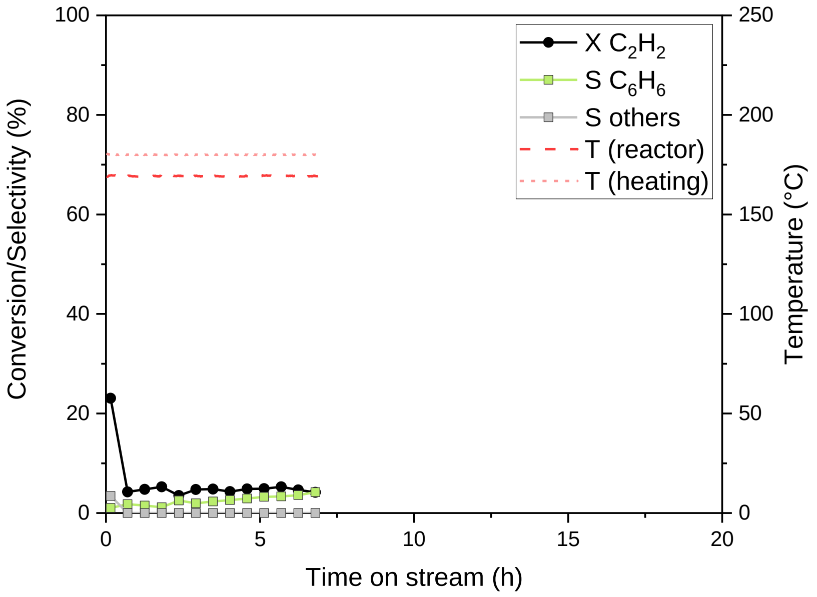

Figure S104: Selectivity to benzene and other volatiles ("others"), acetylene conversion and temperature profiles for 5 wt.% $NbCl_x$-silica gel in the gas-phase cyclotrimerization of acetylene to benzene (C_2H_2/N_2 1:10, 180 °C, 3 bar, WHSV 80 200 $cm^3 h^{-1} g_{Nb}^{-1}$).

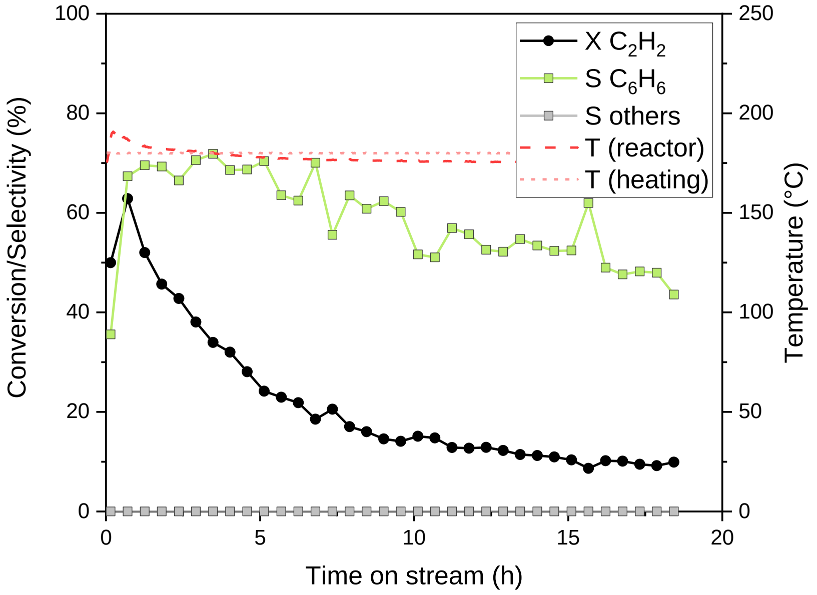

Figure S105: Selectivity to benzene and other volatiles ("others"), acetylene conversion and temperature profiles for 11 wt.% $NbCl_x$-silica gel in the gas-phase cyclotrimerization of acetylene to benzene (C_2H_2/N_2 1:10, 180 °C, 3 bar, WHSV 78 200 $cm^3 h^{-1} g_{Nb}^{-1}$).

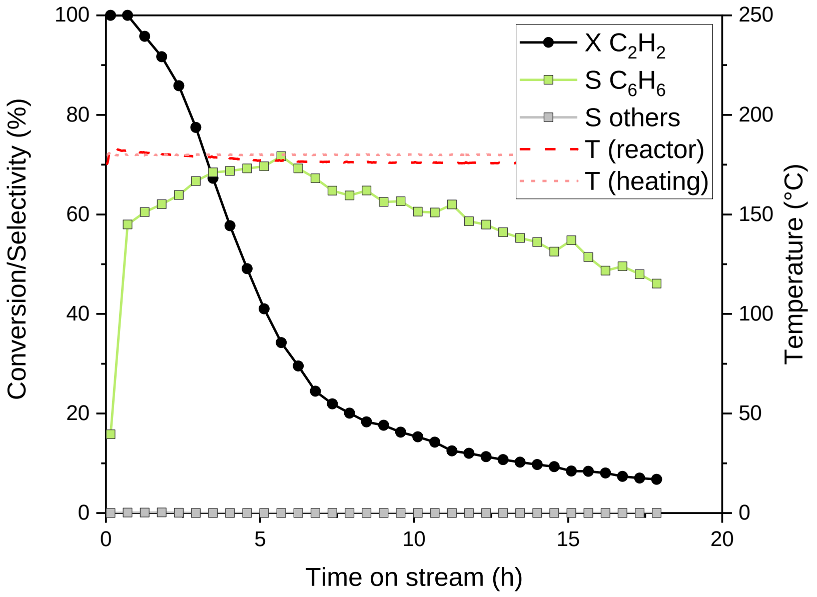

Figure S106: Selectivity to benzene and other volatiles ("others"), acetylene conversion and temperature profiles for 21 wt.% $NbCl_x$-silica gel in the gas-phase cyclotrimerization of acetylene to benzene (C_2H_2/N_2 1:10, 180 °C, 3 bar, WHSV 66 000 $cm^3\ h^{-1}\ g_{Nb}^{\ -1}$).

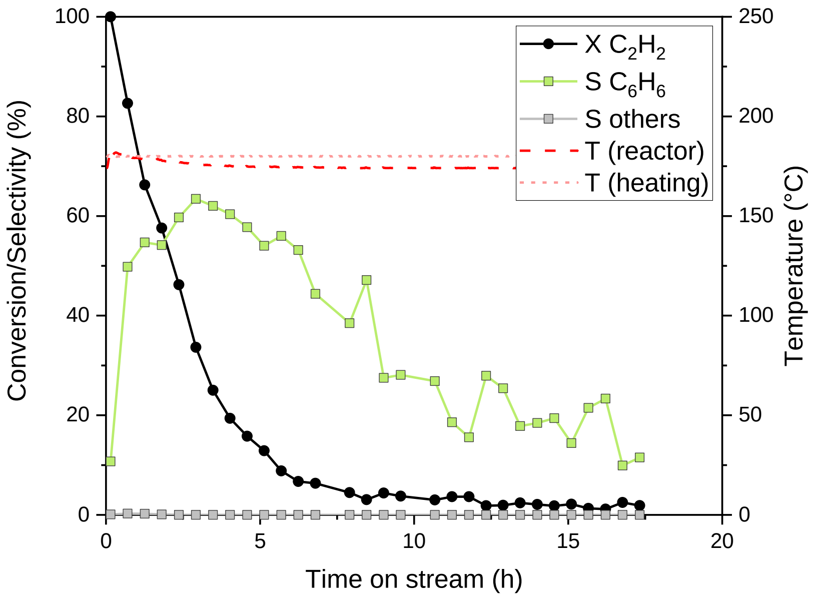

Figure S107: Selectivity to benzene and other volatiles ("others"), acetylene conversion and temperature profiles for 33 wt.% $NbCl_x$-silica gel in the gas-phase cyclotrimerization of acetylene to benzene (C_2H_2/N_2 1:10, 180 °C, 3 bar, WHSV 73 800 $cm^3\ h^{-1}\ g_{Nb}^{\ -1}$).

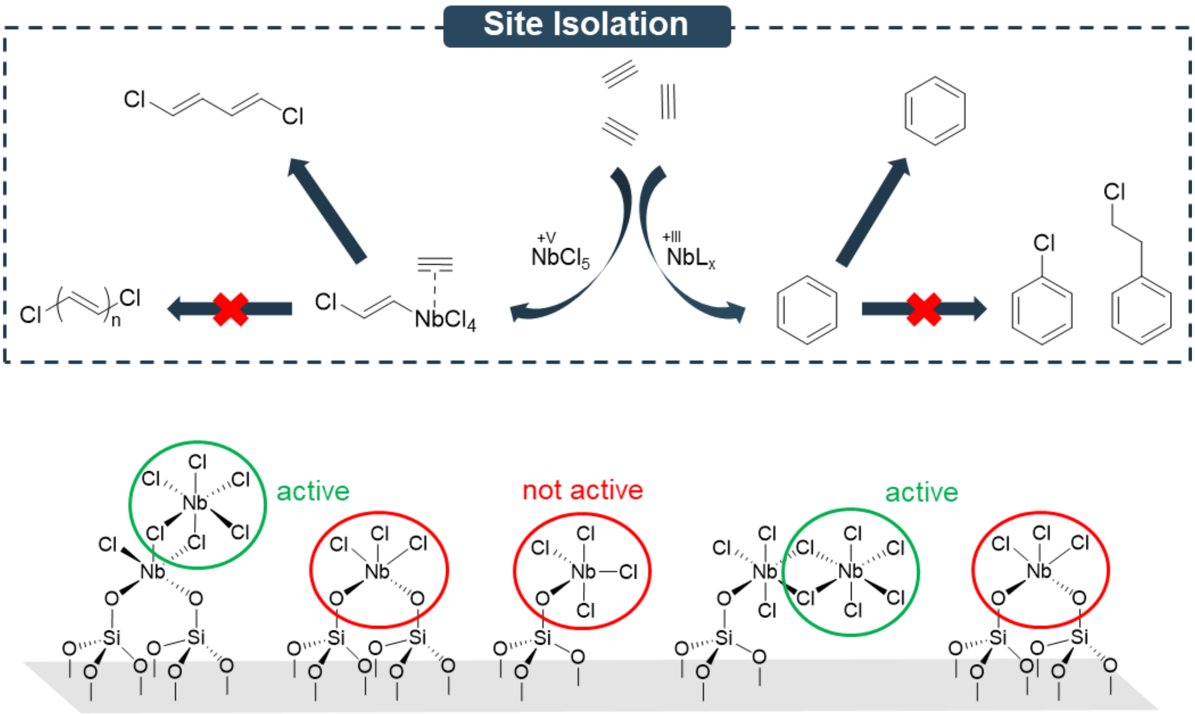

Figure S108: Spatially isolating active dimeric $NbCl_x$ species by inactive monomeric $NbCl_x$ species on the silica gel support surface at lower loadings might allow for a quicker desorption of reaction intermediates (aromatic or polymeric) avoiding sequential reactions (e.g. Friedel-Crafts-Alkylation, chlorination, etc.) to non-volatile compounds.

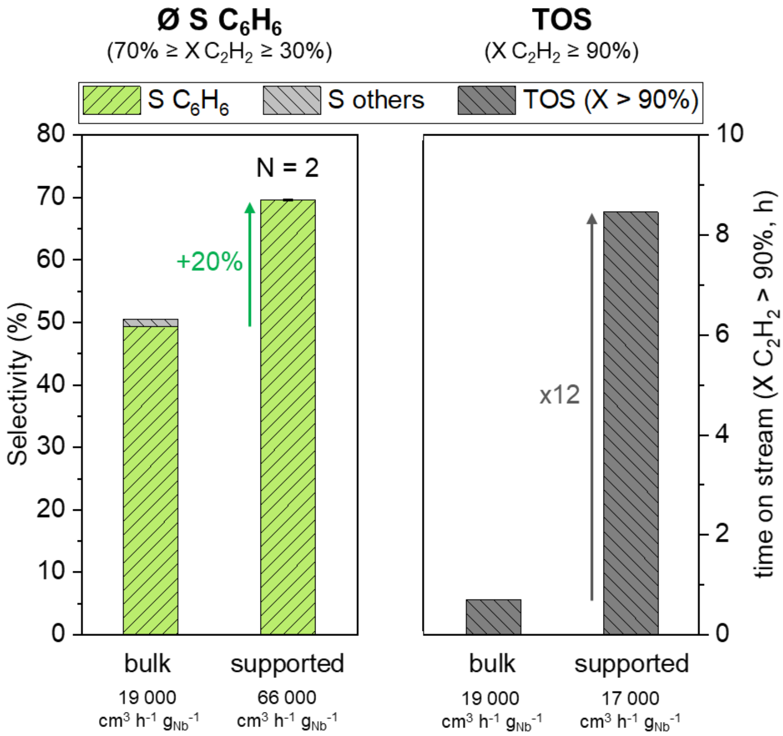

Figure S109: Comparison of the catalytic performance of bulk $NbCl_5$ and 21 wt.% $NbCl_x$-silica gel in the gas-phase cyclotrimerization of acetylene to benzene applying different normalizations. On the left side: Average selectivity to benzene and other volatiles ("others") in an intermediate conversion range of acetylene between 70 and 30% (C_2H_2/N_2 1:10, 180 °C, 3 bar). On the right side: Time on stream (TOS) until initial full acetylene conversion level drops below 90% (C_2H_2/N_2 1:10, 180 °C, 3 bar).

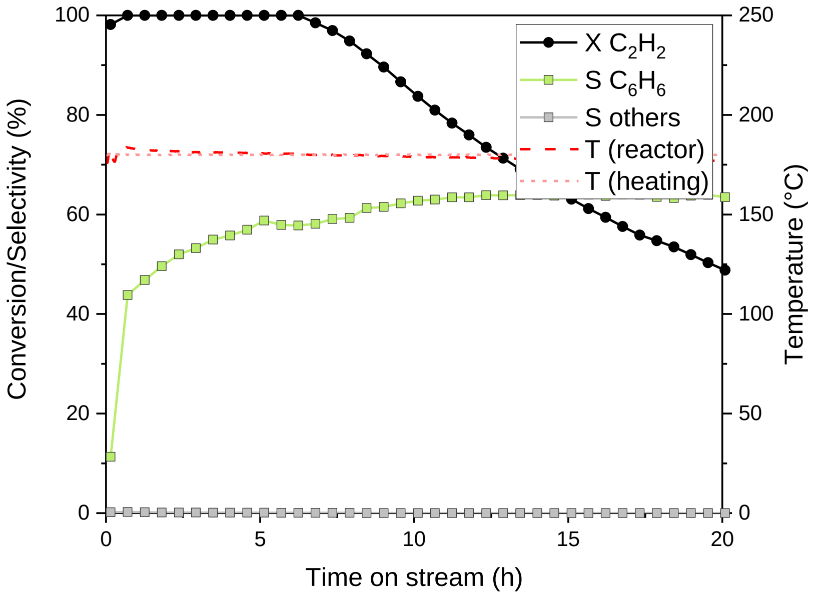

Figure S110: Selectivity to benzene and other volatiles ("others"), acetylene conversion and temperature profiles for 21 wt.% $NbCl_x$-silica gel in the gas-phase cyclotrimerization of acetylene to benzene (C_2H_2/N_2 1:10, 180 °C, 3 bar, WHSV 17 000 $cm^3\ h^{-1}\ g_{Nb}^{-1}$) for the first 20 h on stream.

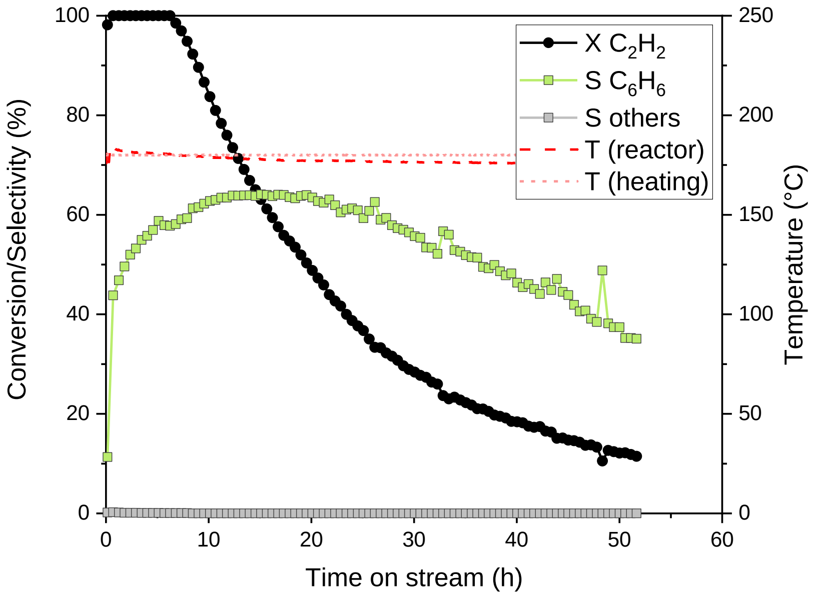

Figure S111: Selectivity to benzene and other volatiles ("others"), acetylene conversion and temperature profiles for 21 wt.% $NbCl_x$-silica gel in the gas-phase cyclotrimerization of acetylene to benzene (C_2H_2/N_2 1:10, 180 °C, 3 bar, WHSV 17 000 $cm^3\ h^{-1}\ g_{Nb}^{-1}$) for over 50 h on stream.

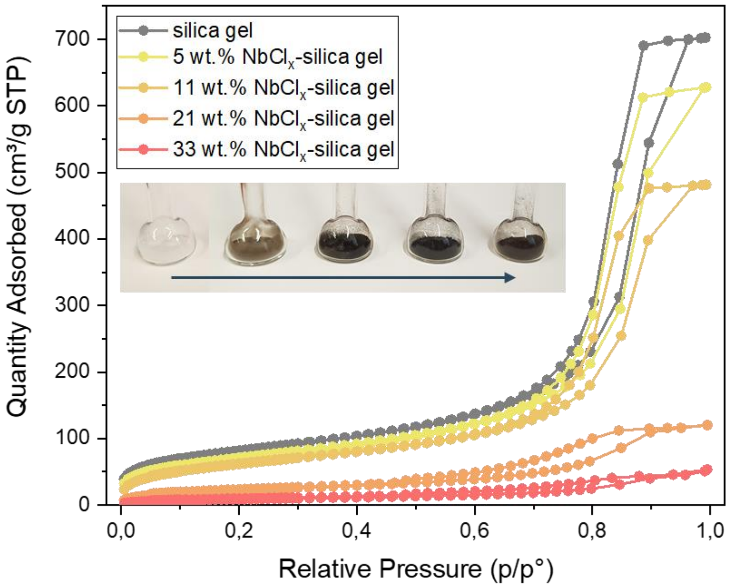

Figure S112: N_2 physisorption data of pristine silica gel and spent $NbCl_x$ functionalized silica gel materials after the gas-phase cyclotrimerization of acetylene to benzene (C_2H_2/N_2 1:10, 180 °C, 3 bar, WHSV ca. 66 000 – 80 000 $cm^3\ h^{-1}\ g_{Nb}^{-1}$). Inlet image shows the respective materials in the order of increasing $NbCl_x$ loading.

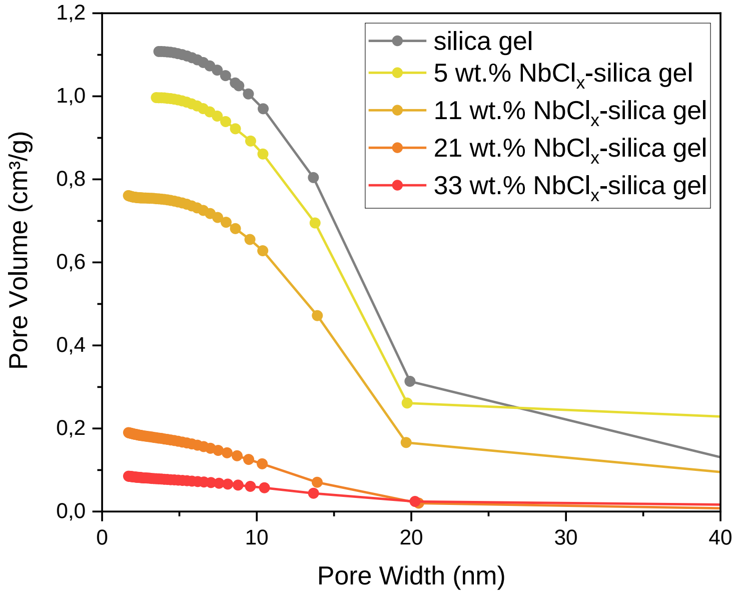

Figure S113: Cumulative pore volume vs. pore size plot derived from the N_2 physisorption data using BJH theory for the pristine silica gel and the spent $NbCl_x$-functionalized silica gels after the gas-phase cyclotrimerization of acetylene to benzene (C_2H_2/N_2 1:10, 180 °C, 3 bar, WHSV ca. 66 000 – 80 000 $cm^3\ h^{-1}\ g_{Nb}^{-1}$).

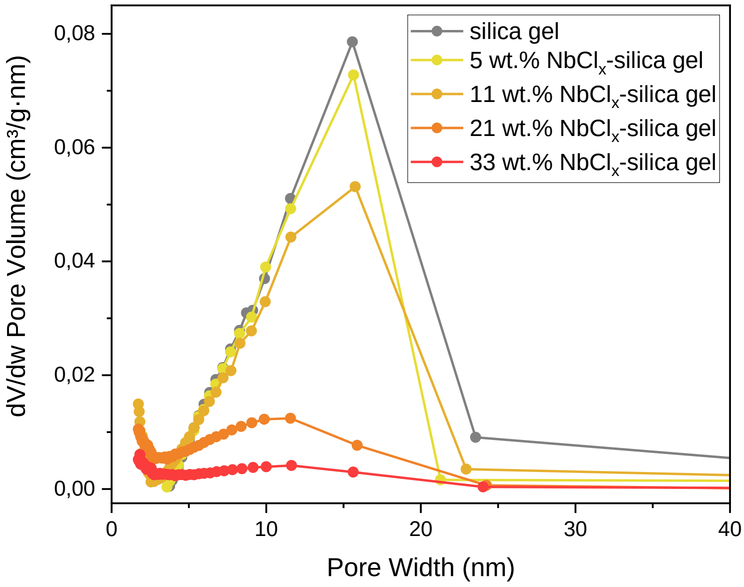

Figure S114: Pore size distribution for the pristine silica gel and the spent NbCl$_x$-functionalized silica gel materials derived from N_2 physisorption data using BJH theory after the gas-phase cyclotrimerization of acetylene to benzene (C_2H_2/N_2 1:10, 180 °C, 3 bar, WHSV ca. 66 000 – 80 000 cm^3 h^{-1} g_{Nb} $^{-1}$).

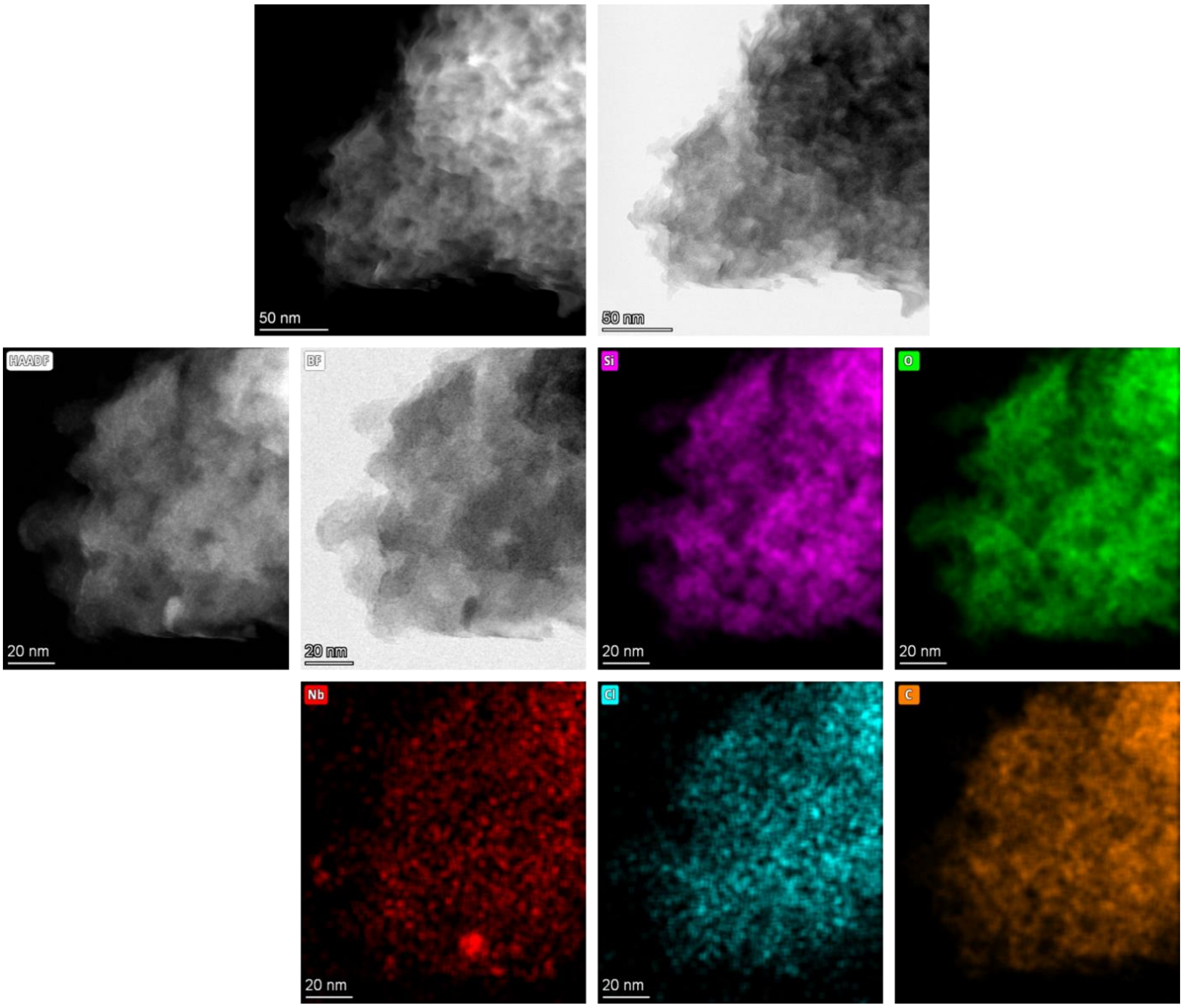

Figure S115: TEM images with elemental mapping of the spent 21 wt.% $NbCl_x$-silica gel material after the gas-phase cyclotrimerization of acetylene to benzene (C_2H_2/N_2 1:10, 150 °C, 3 bar, WHSV ca. 66 000 cm^3 h^{-1} $g_{Nb}{}^{-1}$) revealing the deposition of a carbon overlayer and the partial formation of nanoagglomerates of $NbCl_x$ species.

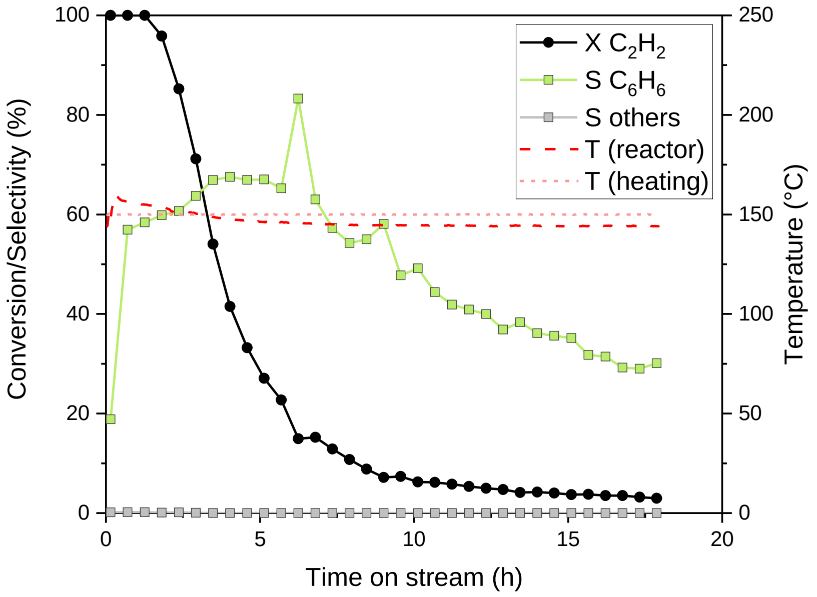

Figure S116: Selectivity to benzene and other volatiles ("others"), acetylene conversion and temperature profiles for 21 wt.% $NbCl_x$-silica gel in the gas-phase cyclotrimerization of acetylene to benzene (C_2H_2/N_2 1:10, 150 °C, 3 bar, WHSV 66 000 $cm^3\ h^{-1}\ g_{Nb}{}^{-1}$).

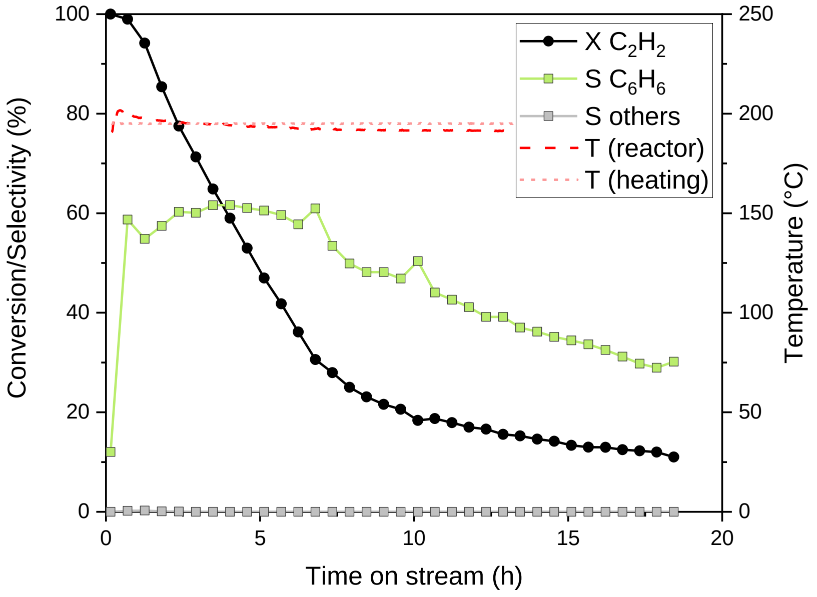

Figure S117: Selectivity to benzene and other volatiles ("others"), acetylene conversion and temperature profiles for 21 wt.% $NbCl_x$-silica gel in the gas-phase cyclotrimerization of acetylene to benzene (C_2H_2/N_2 1:10, 195 °C, 3 bar, WHSV 66 000 $cm^3\ h^{-1}\ g_{Nb}{}^{-1}$).

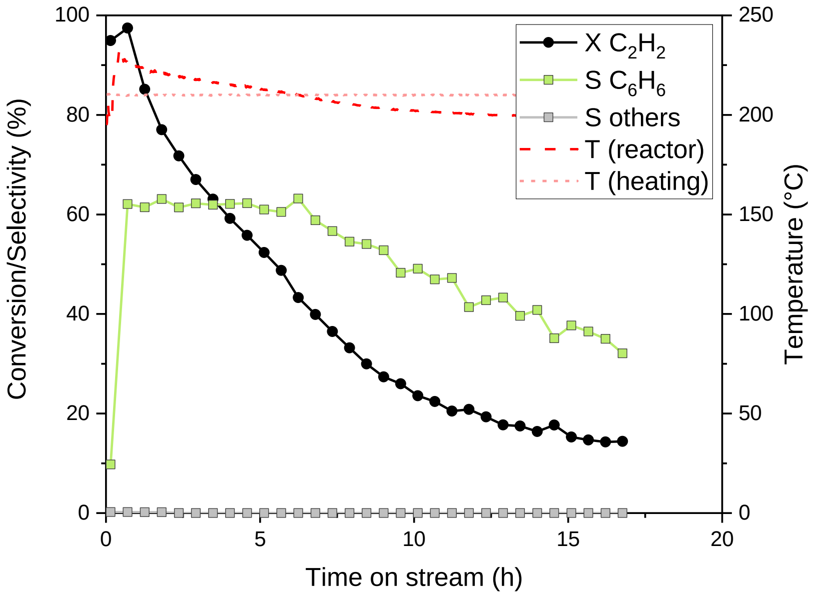

Figure S118: Selectivity to benzene and other volatiles ("others"), acetylene conversion and temperature profiles for 21 wt.% $NbCl_x$-silica gel in the gas-phase cyclotrimerization of acetylene to benzene (C_2H_2/N_2 1:10, 210 °C, 3 bar, WHSV 66 000 $cm^3\ h^{-1}\ g_{Nb}^{-1}$).

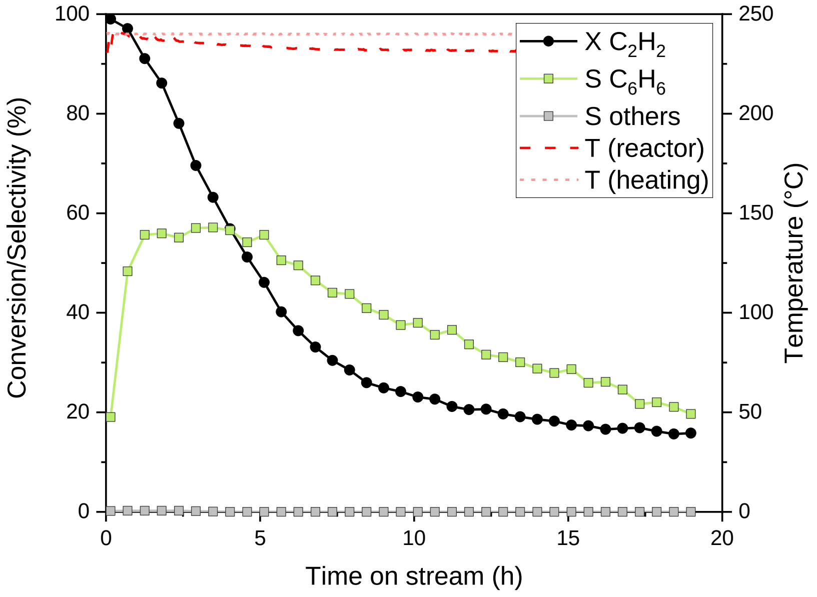

Figure S119: Selectivity to benzene and other volatiles ("others"), acetylene conversion and temperature profiles for 21 wt.% $NbCl_x$-silica gel in the gas-phase cyclotrimerization of acetylene to benzene (C_2H_2/N_2 1:10, 240 °C, 3 bar, WHSV 66 000 $cm^3\ h^{-1}\ g_{Nb}^{-1}$).

REFERENCES

(1) Fairbrother, F.; Nixon, J. F.; Prophet, H. Solubilities of Niobium and Tantalum Pentachlorides in Some Hydrocarbons and Carbon Tetrachloride. *J Less-Common Met* **1965**, *9* (6), 434-+. DOI: Doi 10.1016/0022-5088(65)90127-X.

(2) Fairley, N.; Fernandez, V.; Richard-Plouet, M.; Guillot-Deudon, C.; Walton, J.; Smith, E.; Flahaut, D.; Greiner, M.; Biesinger, M.; Tougaard, S.; et al. Systematic and collaborative approach to problem solving using X-ray photoelectron spectroscopy. *Appl Surf Sci Adv* **2021**, 5. DOI: 10.1016/j.apsadv.2021.100112.

(3) Peredkov, S.; Pereira, N.; Grötzsch, D.; Hendel, S.; Wallacher, D.; DeBeer, S. PINK: a tender X-ray beamline for X-ray emission spectroscopy. *J Synchrotron Radiat* **2024**, *31*, 622-634. DOI: 10.1107/S1600577524002200.

(4) Thompson, A. A., D.; Gullikson, E.; Howells, M.; Kim, K.-J.; Kirz, J.; Kortright, J.; Lindau, I.; Liu, Y.; Pianetta, P.; Robinson, A.; Scofield, J.; Underwood, J.; Williams. G.; Winick, H. *X-Ray Data Booklet*; Lawrence Berkely National Laboratory, University of California, 2009.

(5) Schoonjans, T.; Brunetti, A.; Golosio, B.; del Rio, M. S.; Solé, V. A.; Ferrero, C.; Vincze, L. The xraylib library for X-ray-matter interactions. Recent developments. *Spectrochim Acta B* **2011**, *66* (11-12), 776-784. DOI: 10.1016/j.sab.2011.09.011.

(6) Trotus, I.-T. Catalytic Conversion of Acetylen to Butadien and Butenes. PhD, Ruhr-Universität Bochum, 2016.

(7) Trotus, I. T.; Zimmermann, T.; Duyckaerts, N.; Geboers, J.; Schuth, F. Butadiene from acetylene-ethylene cross-metathesis. *Chem Commun* **2015**, *51* (33), 7124-7127. DOI: 10.1039/c5cc00853k. Kley, K. S.; De Bellis, J.; Schüth, F. Selective hydrogenation of highly concentrated acetylene streams over mechanochemically synthesized PdAg supported catalysts. *Catal. Sci. Technol.* **2022**, 1-13. DOI: 10.1039/d2cy01424f. Agbaba, Ö. T., Ioan-Teodor; Schmidt, Wolfgang; Schüth, Ferdi. Light Olefins from Acetylene under Pressurized Conditions. *Ind Eng Chem Res* **2023**, *62*, 1819-1825. Mauss, J. M.; Schüth, F. On the Role of Anions in Solid Catalysts with Ionic Liquid Layer (SCILL) for the Selective Hydrogenation of Highly Concentrated Acetylene Streams. *Chemsuschem* **2025**, *18* (2), e2024015. DOI: doi.org/10.1002/cssc.202401593. Williams, J. O.; Kitching, E.; Patel, R. S.; Mauss, J. M.; Kley, K. S.; Khobragade, R.; de Bellis, J.; Morgan, D. J.; Slater, T.; Schueth, F.; et al. The Influence of Reaction Conditions on Selective Acetylene Hydrogenation Over Sol Immobilization Prepared $AgPd/Al_2O_3$ Catalysts. *Chemcatchem* **2025**, *17* (8), e202401794. DOI: 10.1002/cctc.202401794. Mauss, J. M.; Kley, K. S.; Khobragade, R.; Tran, N. K.; de Bellis, J.; Schüth, F.; Scheffler, M.; Foppa, L. Modeling Time-On-Stream Catalyst Reactivity in the Selective Hydrogenation of Concentrated Acetylene Streams under Industrial Conditions via Experiments and AI. *Acs Catal* **2025**, *15* (15), 12652-12665. DOI: 10.1021/acscatal.5c02226. Khobragade, R.; Mauss, J. M.; Tran, N. K.; Schüth, F. Exploring milling atmosphere effects in mechanochemical synthesis of Pd-Cu supported catalysts for the semihydrogenation of acetylene in equimolar ethylene mixtures. *Rsc Mechanochem* **2025**, *2*, 923-935. DOI: 10.1039/d5mr00074b.

(8) Dietz, W. A. Response Factors for Gas Chromatographic Analyses. *J Gas Chromatogr* **1967**, *5* (2), 68-&. DOI: DOI 10.1093/chromsci/5.2.68.

(9) Boudjahem, A. G.; Monteverdi, S.; Mercy, M.; Bettahar, M. M. Acetylene cyclotrimerization over Ni/SiO_2 catalysts in hydrogen atmosphere. *Appl Catal a-Gen* **2003**, *250* (1), 49-64. DOI: 10.1016/S0926-860x(03)00221-7.

(10) Duplessis, J. A. K.; Smulders, P.; Dutoit, C. J. The Activation of Tantalum(V) Chloride as Homogeneous Catalyst for the Cyclotrimerization of Acetylene. *J Mol Catal* **1987**, *42* (1), 105-113. DOI: Doi 10.1016/0304-5102(87)85044-7. Dutoit, C. J.; Duplessis, J. A. K.; Lachmann, G. The Cyclotrimerization and Cocyclotrimerization of Phenylacetylene with 1,7-Octadiyne and 1-Hexyne by Niobium(V) Chloride. *J Mol Catal* **1989**, *53* (1), 67-78. DOI: Doi 10.1016/0304-5102(89)85030-8. Lachmann, G.; Duplessis, J. A. K.; Dutoit, C. J. The Role of Niobium(V) Chloride in the Catalytic Cyclotrimerization of Phenylacetylene. *J Mol Catal* **1987**, *42* (2), 151-159. DOI: Doi 10.1016/0304-5102(87)85021-6.

(11) Luo, T. H.; Xu, X. B.; Jiang, M. Q.; Lu, Y. Z.; Meng, H.; Li, C. X. Polyacetylene carbon materials: facile preparation using $AlCl_3$ catalyst and excellent electrochemical performance for supercapacitors. *Rsc Adv* **2019**, *9* (21), 11986-11995. DOI: 10.1039/c9ra01205b.

(12) D'Elia, V.; Dong, H. L.; Rossini, A. J.; Widdifield, C. M.; Vummaleti, S. V. C.; Minenkov, Y.; Poater, A.; Abou-Hamad, E.; Pelletier, J. D. A.; Cavallo, L.; et al. Cooperative Effect of Monopodal Silica-Supported Niobium Complex Pairs Enhancing Catalytic Cyclic Carbonate Production. *J Am Chem Soc* **2015**, *137* (24), 7728-7739. DOI: 10.1021/jacs.5b02872. Zhuravlev, L. T. Concentration of Hydroxyl-Groups on the Surface of Amorphous Silicas. *Langmuir* **1987**, *3* (3), 316-318. DOI: DOI 10.1021/la00075a004. Kessaratikoon, T.; Kaewsai, S.; D'Elia, V. *Realistic Catalysts For The Cycloaddition Of CO_2 To Epoxides Under Ambient Conditions To Generate Cyclic Organic Carbonates: The Case Of Coordination Compounds And Naturally Available Hydrogen Bond Donors*; Societa' Chimica Italiana, 2024. DOI: 10.17374/targets.2024.27.282.

(13) Barbosa, S. L.; Lima, C. D.; Almeida, M. A. R.; Mourao, L. S.; Ottone, M.; Nelson, D. L.; Klein, S. I.; Zanatta, L. D.; Clososki, G. C.; Caires, F. J.; et al. The preparation of benzyl esters using stoichiometric niobium (V) chloride versus niobium grafted SiO_2 catalyst: A comparison study. *Heliyon* **2018**, *4* (3). DOI: 10.1016/j.heliyon.2018.e00571.

(14) Lakshminarayanan, K.; Sivanandhan, M.; Ramasundaram, S.; Oh, T. H.; Shah, K. J.; Saranraj, K.; Parasuraman, A.; Balu, K. $NbCl_5$ Functionalized Perlite: A Potent and Recyclable Catalyst for Synthesis of Pyrans. *Sustainability-Basel* **2023**, *15* (4). DOI: 10.3390/su15043678. Atta, A. A.; Hassanien, A. M.; El-Nahass, M. M.; Shaltout, A. A.; Al-Talhi, Y. A.; Aljoudi, A. M. Influence of argon flow rate on structural and optical properties of transparent Nb_2O_5 thin films. *Opt Quant Electron* **2019**, *51* (10). DOI: 10.1007/s11082-019-2054-y. Umpierres, C. S.; Prola, L. D. T.; Adebayo, M. A.; Lima, E. C.; dos Reis, G. S.; Kunzler, D. D. F.; Dotto, G. L.; Arenas, L. T.; Benvenutti, E. V. Mesoporous Nb_2O_5/SiO_2 material

obtained by sol-gel method and applied as adsorbent of crystal violet dye. *Environ Technol* **2017**, *38* (5), 566-578. DOI: 10.1080/09593330.2016.1202329.
